# Supplementary material for: Effect of PGPR on growth and nutrient utilization of Elymus nutans Griseb at different temperatures
Source: PLoS One. 2025 May 12;20(5):e0323613. doi: 10.1371/journal.pone.0323613 (PMC12068589; doi:10.1371/journal.pone.0323613)

PLOS ONE

Mechanisms of growth promotion and resistance of PGPR to Elymus nutans Griseb at different temperatures

--Manuscript Draft--

**Manuscript Number:**	PONE-D-24-56644
**Article Type:**	Research Article
**Full Title:**	Mechanisms of growth promotion and resistance of PGPR to Elymus nutans Griseb at different temperatures
**Short Title:**	Mechanisms of growth promotion and resistance of PGPR to Elymus nutans Griseb at different temperatures
**Corresponding Author:**	junqiang wang<br>China West Normal University<br>Wuwei, CHINA
**Keywords:**	Plant Growth-Promoting Rhizobacteria (PGPR); Nutrient Uptake; Temperature Effects; Elymus nutans Griseb; Resistance
**Abstract:**	Plant growth-promoting rhizobacteria (PGPR) can promote plant growth, increase nutrient uptake, and resistance. We aimed to examine the effects of temperature on PGPR-mediated effects or PGPR inoculum colonization. The effects of PGPR on Elymus nutans Griseb (EnG) in terms of growth and utilization of nutrients under different temperatures were examined using pot experiments including PGPR in the substrate (T1), no PGPR in the substrate (T2), PGPR and fertilizer in the substrate (T3), and fertilizer in the substrate but no PGPR (T4). Compared with the T2 treatment, the T1 treatment increased plant height by 51.72% (10 °C) to 70.67% (20 °C), and increased dry weight, accounting for 24.99% (15 °C) to 51.25% (10 °C) of EnG. Compared with T2, T1 treatment significantly increased the SS and SP of EnG by 59.37% and 369.66% at 10 °C, by 100.17% and 94.5% at 15 °C, respectively. Compared with T4, T3 significantly decreased the physiological efficiency of N at 15 °C and 20 °C by 40.43% and 72.11%, respectively. Furthermore, the substrate containing both PGPR and fertilizer demonstrated a marked enhancement in the nutrient agronomic efficiency of EnG, suggesting that PGPR strains are capable of optimizing plant nutrient uptake, thereby mitigating the necessity for excessive fertilizer application. The findings of this study offer valuable strain resources and foundational data for the formulation of specialized microbial fertilizers tailored for the alpine meadows of the Qinghai-Tibet Plateau. Moreover, these results provide a theoretical framework for the application of PGPR strains in the sustainable development of alpine meadows on the Qinghai-Tibet Plateau, contributing to the advancement of agricultural practices in this region.
**Order of Authors:**	Linling Ran
	Haoyang Wu
	Fei Xia
	Yunyin Xue
	Wei Wei
	junqiang wang
	Jinglong Wang
	Shanshan Zhao
	Shuang Yan
	Hao Shi
	Shaikun Zheng
	Yu Zhang
	Xiaoqin Qiu

Powered by Editorial Manager® and ProduXion Manager® from Aries Systems Corporation

Additional Information:	
**Question**	**Response**
**Financial Disclosure**<br><br>Enter a financial disclosure statement that describes the sources of funding for the work included in this submission. Review the submission guidelines for detailed requirements. View published research articles from *PLOS ONE* for specific examples.<br><br>This statement is required for submission and **will appear in the published article** if the submission is accepted. Please make sure it is accurate.<br><br>**Funded studies**<br>Enter a statement with the following details:<br>• Initials of the authors who received each award<br>• Grant numbers awarded to each author<br>• The full name of each funder<br>• URL of each funder website<br>• Did the sponsors or funders play any role in the study design, data collection and analysis, decision to publish, or preparation of the manuscript?<br><br>Did you receive funding for this work?	Yes
Please add funding details.<br>as follow-up to "**Financial Disclosure**<br><br>Enter a financial disclosure statement that describes the sources of funding for the work included in this submission. Review the submission guidelines for detailed requirements. View published research articles from *PLOS ONE* for specific examples.<br><br>This statement is required for submission and **will appear in the published article** if the submission is accepted. Please make sure it is accurate.	This work was financially supported by the Science and Technology Program of Tibet Autonomous Region (XZ202201ZY0005N), Sichuan Natural Science Foundation Project (2024NSFSC2074), National Natural Sciences Foundation of China (NO.41867013), China Forestry and Grassland Reform and Development Fund (GZFCG2023-17620), and Innovation Team Funds of China West Normal University (KCXTD2023-5). We also thank the anonymous reviewers for providing critical comments and suggestions that improved the manuscript.

Powered by Editorial Manager® and ProduXion Manager® from Aries Systems Corporation

**Funded studies**<br>Enter a statement with the following details:<br>• Initials of the authors who received each award<br>• Grant numbers awarded to each author<br>• The full name of each funder<br>• URL of each funder website<br>• Did the sponsors or funders play any role in the study design, data collection and analysis, decision to publish, or preparation of the manuscript?<br><br>Did you receive funding for this work?"	
Please select the country of your main research funder (please select carefully as in some cases this is used in fee calculation).<br>as follow-up to "**Financial Disclosure**<br><br>Enter a financial disclosure statement that describes the sources of funding for the work included in this submission. Review the submission guidelines for detailed requirements. View published research articles from *PLOS ONE* for specific examples.<br><br>This statement is required for submission and **will appear in the published article** if the submission is accepted. Please make sure it is accurate.<br><br>**Funded studies**<br>Enter a statement with the following details:<br>• Initials of the authors who received each award<br>• Grant numbers awarded to each author<br>• The full name of each funder<br>• URL of each funder website<br>• Did the sponsors or funders play any role in the study design, data collection and analysis, decision to publish, or preparation of the manuscript?<br><br>Did you receive funding for this work?"	CHINA - CN
**Competing Interests**	The authors declare that the research was conducted in the absence of any

Use the instructions below to enter a competing interest statement for this submission. On behalf of all authors, disclose any competing interests that could be perceived to bias this work—acknowledging all financial support and any other relevant financial or non-financial competing interests.<br><br>This statement is required for submission and **will appear in the published article** if the submission is accepted. Please make sure it is accurate and that any funding sources listed in your Funding Information later in the submission form are also declared in your Financial Disclosure statement.<br><br>View published research articles from *PLOS ONE* for specific examples.<br><br>**NO authors have competing interests**<br><br>Enter: *The authors have declared that no competing interests exist.*<br><br>**Authors with competing interests**<br><br>Enter competing interest details beginning with this statement:<br><br>*I have read the journal's policy and the authors of this manuscript have the following competing interests: [insert competing interests here]*<br><br>* typeset	commercial or financial relationships that could beconstrued as a potential conflict of interest.
**Ethics Statement**<br><br>Enter an ethics statement for this submission. This statement is required if the study involved:<br><br>• Human participants<br>• Human specimens or tissue<br>• Vertebrate animals or cephalopods<br>• Vertebrate embryos or tissues	No.

• Field research<br><br>Write "N/A" if the submission does not require an ethics statement.<br><br>General guidance is provided below. Consult the submission guidelines for detailed instructions. **Make sure that all information entered here is included in the Methods section of the manuscript.**	

**Format for specific study types**<br><br>**Human Subject Research (involving human participants and/or tissue)**<br>• Give the name of the institutional review board or ethics committee that approved the study<br>• Include the approval number and/or a statement indicating approval of this research<br>• Indicate the form of consent obtained (written/oral) or the reason that consent was not obtained (e.g. the data were analyzed anonymously)<br><br>**Animal Research (involving vertebrate animals, embryos or tissues)**<br>• Provide the name of the Institutional Animal Care and Use Committee (IACUC) or other relevant ethics board that reviewed the study protocol, and indicate whether they approved this research or granted a formal waiver of ethical approval<br>• Include an approval number if one was obtained<br>• If the study involved *non-human primates*, add *additional details* about animal welfare and steps taken to ameliorate suffering<br>• If anesthesia, euthanasia, or any kind of animal sacrifice is part of the study, include briefly which substances and/or methods were applied<br><br>**Field Research**<br><br>Include the following details if this study involves the collection of plant, animal, or other materials from a natural setting:<br>• Field permit number<br>• Name of the institution or relevant body that granted permission	
**Data Availability**<br><br>Authors are required to make all data underlying the findings described fully available, without restriction, and from the time of publication. PLOS allows rare exceptions to address legal and ethical concerns. See the PLOS Data Policy and FAQ for detailed information.	Yes - all data are fully available without restriction

A Data Availability Statement describing where the data can be found is required at submission. Your answers to this question constitute the Data Availability Statement and **will be published in the article**, if accepted.<br><br>**Important:** Stating 'data available on request from the author' is not sufficient. If your data are only available upon request, select 'No' for the first question and explain your exceptional situation in the text box.<br><br>Do the authors confirm that all data underlying the findings described in their manuscript are fully available without restriction?	
**Describe where the data may be found in full sentences. If you are copying our sample text, replace any instances of XXX with the appropriate details.**<br><br>• If the data are **held or will be held in a public repository**, include URLs, accession numbers or DOIs. If this information will only be available after acceptance, indicate this by ticking the box below. For example: *All XXX files are available from the XXX database (accession number(s) XXX, XXX.*).<br>• If the data are all contained **within the manuscript and/or Supporting Information files**, enter the following: *All relevant data are within the manuscript and its Supporting Information files.*<br>• If neither of these applies but you are able to provide **details of access elsewhere**, with or without limitations, please do so. For example:<br><br>*Data cannot be shared publicly because of [XXX]. Data are available from the XXX Institutional Data Access / Ethics Committee (contact via XXX) for researchers who meet the criteria for access to confidential data.*<br><br>*The data underlying the results presented in the study are available from (include the name of the third party*	Data used in this study can be found in Figshare (https://doi.org/10.6084/m9.figshare.27094078.v1).

and contact information or URL).<br>• This text is appropriate if the data are owned by a third party and authors do not have permission to share the data.<br><br>* typeset	
Additional data availability information:	

Powered by Editorial Manager® and ProduXion Manager® from Aries Systems Corporation

Click here to access/download;Manuscript;Manuscript File(1).docx

Mechanisms of growth promotion and resistance of PGPR to *Elymus nutans* Griseb at different temperatures

Linling Ran [a,1], Haoyang Wu [a], Fei Xia [b,c,1], Yunyin Xue [d], Wei Wei [b,c], Junqiang Wang [a,*], Jinglong Wang[b,c], Shanshan Zhao [a], Shuang Yan [a], Hao Shi[e], Shaikun Zheng[e], Yu Zhang [a], Xiaoqin Qiu [a]

[a] Key Laboratory of Southwest China Wildlife Resource Conservation (Ministry of Education). China West Normal University, Nanchong, 637009, China

[b] State Key Laboratory of Highland Barley and Yak Germplasm Resources and Genetic Improvement, Lhasa, 850000, China

[c] Institute of Pratacultural Science, Tibet Academy of Agriculture and Animal Husbandry Science, Lhasa, 850000, China

[d] School of Geography and Tourism, Shaanxi Normal University, Xi'an, 710119, China,

[e] College of Forestry, Gansu Agricultural University, Lanzhou, 730070, China

[*] Corresponding author at: Key Laboratory of Southwest China Wildlife Resource Conservation (Ministry of Education). China West Normal University, Nanchong,637009, China.

[1] These authors contributed equally to this work and should be considered co-first authors.

Tel: 0086/457/17390270491.

E-mail address: wangjunq0303@163.com (J.-Q. Wang).

Abstract

Plant growth-promoting rhizobacteria (PGPR) can promote plant growth, increase nutrient uptake, and resistance. We aimed to examine the effects of temperature on PGPR-mediated effects or PGPR inoculum colonization. The effects of PGPR on *Elymus nutans* Griseb (*En*G) in terms of growth and utilization of nutrients under different temperatures were examined using pot experiments including PGPR in the substrate (T1), no PGPR in the substrate (T2), PGPR and fertilizer in the substrate (T3), and fertilizer in the substrate but no PGPR (T4). Compared with the T2 treatment, the T1 treatment increased plant height by 51.72% (10 °C) to 70.67% (20 °C), and increased dry weight, accounting for 24.99% (15 °C) to 51.25% (10 °C) of *En*G. Compared with T2, T1 treatment significantly increased the SS and SP of *En*G by 59.37% and 369.66% at 10 °C, by 100.17% and 94.5% at 15 °C, respectively. Compared with T4, T3 significantly decreased the physiological efficiency of N at 15 °C and 20 °C by 40.43% and 72.11%, respectively. Furthermore, the substrate containing both PGPR and fertilizer demonstrated a marked enhancement in the nutrient agronomic efficiency of *En*G, suggesting that PGPR strains are capable of optimizing plant nutrient uptake, thereby mitigating the necessity for excessive fertilizer application. The findings of this study offer valuable strain resources and foundational data for the formulation of

specialized microbial fertilizers tailored for the alpine meadows of the Qinghai-Tibet Plateau. Moreover, these results provide a theoretical framework for the application of PGPR strains in the sustainable development of alpine meadows on the Qinghai-Tibet Plateau, contributing to the advancement of agricultural practices in this region.

Keywords: Plant Growth-Promoting Rhizobacteria (PGPR); Nutrient Uptake; Temperature Effects; *Elymus nutans* Griseb; Resistance

1. Introduction

Under natural conditions, plants perpetually engage in intricate relationships with a microbial community known as the phytomicrobiome [1]. Certain soil microorganisms contribute to the enhancement of plant growth and progression [2]. Plant growth-promoting rhizobacteria (PGPR), a type of beneficial bacteria found in nature, inhabit plant roots and enhance plant development [3]. PGPR encompasses numerous soil bacteria species that promote the growth of host plants when cultivated in their presence [4]. These microbes populate the root zone of plants and boost plant growth either directly or indirectly [5]. [6] showed that the application of *Bacillus megaterium* and *Bacillus mucilaginous* as microbial inoculants enhanced plant growth and enhanced the plant's uptake of nutrients (total N, P, and K). These microbes play essential roles in the solubilization of P and K and nitrogen fixation. They transform insoluble P and K in the soil into plant-accessible forms via acidolysis, chelation, and exchange reactions, which in turn stimulate plant growth [7]. In addition, PGPR can regulate phytohormone levels in plants and thereby influence plant growth [8].

Plants face diverse environmental challenges that substantially hamper their growth and progression, potentially causing significant crop losses in farming. Cold temperatures represent a key non-biological element that restricts the growth and output of plants [9]. There is extensive documentation that suitable management techniques, encompassing the use of fertilizers and external protective compounds, can reduce the harmful influence of cold stress on plant growth [10]. PGPR which is resistant to cold enhances plant nutrient uptake and accessibility via biochemical and physiological processes [11].[12] revealed that specific PGPR strains can impart cold resistance to rice plants, boosting their viability, fertility, and yield-related traits and accelerating the progression of their reproductive cycle. Furthermore, variation in temperature tolerance exists among PGPR strains, affecting efficacy and plant health [13].

Elymus nutans Griseb *(En*G*)* serves as a native and widespread grazing grass commonly found in the

highland pastures of the Qinghai-Tibetan Plateau in China [14]. The grass is widely applied in cattle farming and ecological protection, owing to its high nutritional quality and ability to withstand low temperatures [15]. The growth of *En*G relies on favorable relationships between its roots and microbes, which are essential for nutrient uptake and enhancing growth in its habitat [16]. Therefore, investigating the PGPR inhabiting the rhizosphere of *En*G on the Qinghai-Tibetan Plateau is crucial. Several studies demonstrate that PGPR can fix N, solubilize P, detoxify K, and secrete indole-3-acetic acid (IAA) to promote growth [17–20]. Nonetheless, few studies examined the effects of temperature on PGPR-mediated effects or PGPR inoculum colonization [21]. To systematically elucidate the growth-promoting mechanism of PGPR at different temperatures and to enhance the yield of *En*G, we aimed to: (1) investigate the effects of PGPR on the growth and nutrient utilization of *En*G and (2) on *En*G at different temperatures. The findings of this study help identify valuable microbial resources and provide foundational insights, thus supporting the development and application of PGPR as a biofertilizer to enhance pasture productivity on the Tibetan Plateau.

2. Materials and methods

2.1. Identification of PGPR strains

The inter-root sampling area is located in Nagqhu Town, Seni District, Nagqhu City, Tibet Autonomous Region, with geographic coordinates of 31°26′N latitude and 92°07′E longitude (Fig. 1), and an average elevation of 4450 m. The average annual temperature is -2.9 ℃, the average temperature in the coldest month (January) is -20 ℃, and the average temperature in the hottest month (July) is 8 ℃, the average annual precipitation is about 430 mm, the annual relative humidity is about 50%, the precipitation is mainly concentrated in June-September, and there is no absolute frost-free period. A total of 273 strains were isolated from the inter-root soil of well-grown forage grasses at three randomly collected sites in the region. These isolates were preserved on Luria Bertani (LB) agar and stored at a temperature of 28°C. Five strains (S1, S2, S3, S4, and S5) with excellent efficacy were selected and were inoculated in LB liquid medium for incubation at 37 °C under shaking (160 rpm) for 24 h. Bacterial genomic DNA was extracted from these strains using a commercial kit (Tiangen Biochemical Technology, Beijing, China). The 16S rRNA gene was polymerase chain reaction (PCR) amplified with the primer set 27 F (5′-AGAGTTTGATCCTGGCTCAG-3′) and 1492R (5′-GGTTACCTTGTTACGACTT-3′) as described by Galkiewicz and Kellogg (2008) [22]. PCR was executed in a 50 μL volume, comprising 1 μL of 5 μM forward primer, 1 μL of 5 μM reverse primer, 25 μL of 2×Taq PCR MasterMix, 2 μL of DNA template, and double-distilled water. The PCR thermal cycling parameters were set to an initial denaturation at 95 °

C for 5 minutes, followed by 30 cycles of 95 °C for 30 seconds, 57 °C for 60 seconds, and 72 °C for 30 seconds, with a concluding extension phase at 72 °C for 10 minutes. The PCR results were validated through 1% agarose gel electrophoresis and subsequently subjected to sequencing via the dideoxy chain-termination approach at Sangon Biotech Co., Ltd. (Shanghai, China) [23]. The sequences of the 16S rRNA genes from the five bacterial strains have been submitted and archived in the GenBank repository, and corresponding accession numbers have been assigned: PQ351188, PQ351189, PQ351190, PQ351191, and PQ351192.

2.2. Determination of plant growth promotion characteristics of PGPR strains

The ability of the isolates to grow on NFM agar and their nitrogen-fixing capacity was evaluated using the acetylene reduction method [24]. Strains' phosphorus solubilizing capacity was tested with Mo blue colorimetry [25]. The strains were cultured in 50 mL King's broth at 28 °C with shaking (180 rpm) for 3 days. IAA was extracted from the culture filtrate using ethyl acetate, with each extraction done three times [26]. The IAA content was measured using high-performance liquid chromatography [27]. The KSB-containing solution was diluted to 50 mL and centrifuged at 500 rpm for 10 minutes to eliminate solids. Subsequently, a 10 mL aliquot was further centrifuged at 10,000 rpm for 10 minutes, and the K in the supernatant was quantified using flame spectrophotometry [28].

2.3 Pot experiment

We conducted a pot experiment to evaluate the effect of isolates S1 to S5 on the growth of *En*G. All five strains solubilized inorganic and organic phosphates, decomposed K, and secreted IAA. In addition, S1, S3, S4, and S5 fixed N (Table 1). Cultures of bacteria (S1-S5) were subjected to centrifugation at 8000 g for 10 minutes at 4 °C. The pelleted bacteria were then re-centrifuged and rinsed thrice with sterile H_2O before being adjusted to a final OD600 of 0.6 with sterile water, serving as the prepared inoculum [29]. Plant seeds (wild *Elymus nutans* Griseb seeds collected in Nagqhu, Tibet) were sterilized on the surface with a 1:1 mixture of 30% hydrogen peroxide and 70% ethanol for 10 minutes and cleaned repeatedly with demineralized sterile water. The growth medium was composed of a blend of peat soil, vermiculite, perlite, and coir in a ratio of 4:1:1:1 (v/v), with 50 g per bag, which was then sterilized at 121 °C for 30 minutes. Subsequently, 78.2 g of the culture medium was combined with 1% (volume/volume) PGPR inoculum and transferred into a 660 mL plastic container (70 × 95 × 98 mm) [30]. A cup inoculated with an equal volume of sterile water was used as the control (T2). Four treatments were established in this study: culture substrate with PGPR bacterial cultures (T1), with equivalent amounts of sterile water (T2), N (150 mg kg^{-1}), P (120 mg kg^{-1}), and K (90 mg kg^{-1}), and PGPR (T3), with equivalent amounts of N, P,

K, and water (T4). Three replicates of each treatment were used, which were incubated at 10 °C, 15 °C, and 20 °C with light (16 h)/dark (8 h) cycles (lights at 350 μE m^{-2} s^{-1}) and 60% humidity. Thirty-five *En*G seeds were planted in each cup. In order to better simulate the plant growth cycle in alpine meadows, plant growth indicators were measured after 120 days of plant growth (based on the local plant growth season).

2.4 Dry weight (DW), plant height (PT), and root system assay

A random sample of 15 plants per pot was taken to determine plant height (PT). After drying at 65 °C for 24 h, the dry weight (DW) was noted. Root length (RL), the count of root branches (RB), root surface area (RSA), and the average root diameter (RD) were all assessed using an LA2400 root scanner (Jeili Electronics Trading Co., Ltd, Shanghai, China) [31].

2.5 Plant chlorophyll (Chl), soluble sugars (SS), and soluble proteins (SP) content assay

To measure the chlorophyll (Chl) content, 0.1 g of fresh leaf tissue from each seedling was mixed with a bit of quartz sand, ground in 80% acetone, and then centrifuged at 9000 × g and 4 °C for 10 minutes. The supernatant's absorbance was read at 440, 663, and 645 nm to assess Chl a, b, and carotenoid levels, respectively. The total Chl content was computed using [16]. The soluble sugar (SS) content was assessed following the method of [32]. Soluble protein (SP) content was determined using Bradford's method for staining with Caulmer Brilliant Blue [33].

2.6 Plant nutrient use efficiency

The physiological efficiency (PE) of N (NPE), P (PPE), and K (KPE), i.e., the kg plant yield N, P, and K uptake, was calculated according to Isfan (1990) [34]. The agronomic efficiencies (AE) of N (NAE), P (PAE), and K (KAE), i.e., the yield increase for each kg N, P, K applied, was calculated according to Novoa and Loomis (1981) [35]. The apparent recovery efficiency (RE) of N (NRE), P (PRE), and K (KRE), i.e., plant N, P, and K uptake (kg ha^{-1}) per kg of N, P, and K applied, was calculated according to Fageria and Baligar (2005) [36].

2.7 Statistical analyses

Statistical analyses were performed using SPSS software (version 27.0; SPSS Inc., Chicago, IL, USA). Mean differences were determined using the least significant difference method and the Tukey test at a significance level of 5%. Statistical significance is reported at $p < 0.05$. All visualizations were produced using R 4.4.1. The relationship between the growth indicators of *En*G and nutrient utilization was analyzed through Mantel analysis.

3. Result

*3.1 Effects of PGPR on En*G *growth characteristics at different temperatures*

Substrate with PGPR microbial inoculants influenced the growth characteristics of *En*G (Fig.2). Compared to T2, treatment T1 increased PT by 51.72% (10 °C) to 70.67% (20 °C), and increased DW, accounting for 24.99% (15 °C) to 51.25% (10 °C) (Fig.3). Compared to T4, treatment T3 significantly increased PT by 30.66% (20 °C) to 55.22% (10 °C), and significantly increased DW, accounting for 4.86 % (10 °C) to 94.71% (20 °C) (p<0.05) (Fig.3a and 3c). Treatments T1 and T3 significantly increased the Chl of *EnG* compared to T2 and T4 (p<0.05) (Fig.3c).

*3.2 Effects of PGPR on En*G *root growth at different temperatures*

The effects of PGPR on the roots of *En*G are shown in Fig.4. Compared to T2, treatment T1 significantly increased RSA of *En*G at 10 °C (by 96.32%) and 15 °C (by 109.31%), respectively (p<0.05) (Fig.4). Compared with the T4 treatment, the T3 treatment increased the RL, RB, RSA, and RD of *En*G. Furthermore, the RL of *En*G was significantly higher at 15 °C than at 20 °C (p<0.05) (Fig.4a).

*3.3 Effects of PGPR on SS and SP of En*G *at different temperatures*

Compared to treatment T2, T1 significantly increased the SS and SP of *En*G by 59.37% and 369.66% at 10 °C, by 100.17% and 94.5% at 15 °C, respectively (p<0.05) (Fig.5). Furthermore, the content of SS at 10 °C under the T3 treatment was significantly higher than that of SS at 20 °C (Fig. 5a). The contents of SS and SP at 10 °C under T1 were significantly higher than at 20 °C (p< 0.05) (Fig.5b).

*3.4 Effects of PGPR on En*G *nutrients use efficiency indexes at different temperatures*

Compared with treatment T4, T3 significantly decreased NPE of *En*G at 15°C and 20°C by 40.43% and 72.11%, respectively (p<0.001) (Fig.6a). Moreover, Compared with T4, T3 significantly decreased the PRE of *En*G (p<0.001) (Fig.6h). Furthermore, the T3 treatment significantly increased the NAE, PAE, KAE, NRE, and KRE of *En*G at 15°C and 20°C (p<0.001) (Fig.6).

3.5 Relationship between PGPR and growth indicators of EnG

The PGPR significantly affected PT, DW, Chl, RL, RSA, RD, SS, and SP (Table 2). The interaction between temperature and PGPR significantly affected the RL of *En*G (Table 2) (p<0.001). In addition, RL was significantly and positively correlated with AE (Fig.7b) (p<0.05). PT and DW were highly significantly correlated with NAE, PAE, and KAE (Fig. 7a) (p<0.001), and PT and DW were significantly correlated with the apparent NRE, PRE, and KRE (Fig.7a) (p<0.05). In addition, RL was significantly and

positively correlated with AE (Fig.7) (p<0.05).

4. Discussion

*4.1 The effects of PGPR on En*G *growth*

The plant microbiome has been recognized as a key plant characteristic influencing crop yield. Olasupo et al. (2021) [37] applied various bacillus treatments to peppers, resulting in a 27%–36% boost in biomass. Similarly, Aw et al. (2020) [38] isolated three As-resistant PGPR strains to verify their roles in promoting rice growth. In the present study, compared to the T2 treatment, the T1 treatment increased the PT and DW of *En*G (Fig.3). These findings corroborated the efficacy of PGPR in augmenting the growth of *En*G. Furthermore, the T3 treatment significantly increased the PT and DW of *En*G compared with T4 (p<0.05) (Fig.3a and 3c). There was also a significant correlation between PT, DW, AE, and RE (Fig.7a) (p<0.05). This correlation underscores the role of the culture substrate with PGPR in facilitating *En*G nutrient uptake. Consequently, the application of PGPR can improve soil nutrient use effectively and reduce the amount of chemical fertilizer applied and the negative effects of excessive fertilization [39], thereby, *En*G biomass can be increased, and economic and ecological development can be achieved.

Plant growth requires the accumulation of photochemical products and is directly linked to the photosynthetic rate [40]. del Rosario Cappellari et al. (2015) [41] evaluated the growth characteristics and Chl levels in peppermint (*Mentha piperita*) seedlings exposed to PGPR strains, noting that the treated plants demonstrated improved values for these metrics compared to the untreated controls. Consistent with these findings, our T1 treatment significantly increased the Chl content of *En*G compared with T2 (p<0.05) (Fig.3c). This indicates that the culture substrate with PGPR had a certain promoting effect on the photosynthesis of *En*G.

We found that treatment T1 significantly increased the RSA of *En*G at 15 °C by 109.31% compared with treatment T2 (p<0.05) (Fig.4). Earlier research implied that the impact of PGPR on root growth is connected to the synthesis of IAA, a substance known to advance root expansion and enhancement, augment the area of the root surface, and stimulate root metabolic processes [17]. Consistent with these findings, the culture substrate containing PGPR elicited IAA production (Table 1). Therefore, the enhanced growth of *En*G that was observed in the culture substrate with PGPR was attributed to auxin secretion. Moreover, there was a significant correlation between RSA levels and PE (Fig.7) (p<0.01). PGPR not only alters root structure and enhances root growth but also plays a role in the pathways that regulate plant development and nutrition [42]. This facilitates plants to access a greater range of nutrients from the soil [4], thereby enhancing their growth and development [43]. Ultimately, this results in superior

plant health and crop yield, in terms of quantity and quality [44].

*4.2 The effect of PGPR on the nutrient utilization of En*G

N, P, and K are fundamental chemical elements essential for plant development, contributing significantly to plant tissue formation and physiological processes [45]. The effect of PGPR on plant nutrition stems from its impact on nutrient absorption by plants [46]. Wu et al. (2005) [6] found that Bacillus megaterium and Bacillus mucilaginous inoculants not only boosted plant growth but also enhanced the plants' nutrient absorption capabilities, particularly in terms of total N, P, and K. The root system of plants performs several functions, including nutrient acquisition, water uptake, stability, and symbiotic relationships with beneficial soil microorganisms, which collectively enhance nutrient absorption efficiency [47]. In the present study, the AE and apparent RE were significantly correlated with RL (Fig. 7a). In addition, NAE, PAE, KAE, and NRE were significantly positively correlated with RL (Fig.7b).These findings affirm that efficient nutrient uptake relies significantly on the root system's capacity to permeate the soil [48].

Compared with treatment T4, T3 significantly decreased the NPE of *En*G at 20 °C by 72.11% (p<0.001) (Fig. 6a). The reason for this discrepancy may be the different capacities of PGPR to convert plant-unavailable nutrient forms into plant-available forms [49], which reduces the plants' need for fertilizer. PGPR plays a significant role in agriculture by facilitating the circulation of plant nutrients and reducing the reliance on chemical fertilizers [50]. Our findings offer microbial resources and foundational data for the development and application of PGPR as biofertilizers; however, compared with t T4, the T3 treatment significantly decreased the PRE of *En*G (p<0.001) (Fig.6h). This aligns with previous research indicating that the use of PGPR can enhance P mobilization and supply to crops in soils deficient in P [51]. Therefore, we propose that the culture substrate with PGPR promotes nutrient uptake by *En*G and can help reduce the use of chemical fertilizers in agriculture.

4.3 The effects of PGPR at different temperatures

Under low-temperature stress conditions, plants exhibit a heightened accumulation of SS such as sucrose, glucose, fructose, ribose, and trehalose [52]. These sugars are capable of neutralizing free radicals and indirectly stimulating protein synthesis, which in turn enhances cold resistance [53]. SP are also pivotal, serving as osmoregulators and supporting low-temperature tolerance in plants [54]. Previous studies show that the contents of SP increased up to 68% in plants treated with PGPR [55]. Additionally, the SS content was significantly elevated in PGPR-treated plants in a previous study, and it was

approximately double that of the untreated controls [52]. Our findings corroborate these observations as compared with treatment T2, T1 significantly increased the SS and SP content of *En*G by 59.37% and 369.66% at 10 °C (Fig. 5). This suggests that the physiological and biochemical status of *En*G significantly improved under the influence of PGPR [56]. Thus, inoculating plants with PGPR can be a productive method to foster growth by enhancing their tolerance to various abiotic stresses [57]. Moreover, the content of SS and SP under the T1 treatment was significantly higher at 10 °C than at 20 °C ($p < 0.05$) (Fig. 5b), The accumulation of stress metabolites and protective agents in PGPR varies with external temperatures, which in turn impacts the plant growth-promoting characteristics of these bacteria [58]. This shows that this PGPR is highly adaptable to low temperatures, which provide a valuable resource for the subsequent establishment of a library of plant-promoting strains on the Tibetan Plateau.

Temperature plays a key role in restricting the distribution and output of plants [59]. Temperature exerts distinct influences on plants [60]. In our study, irrespective of PGPR, temperature had a significant effect on the growth of *En*G (Table 2); however, under the influence of PGPR, *En*G was more productive after inoculation with PGPR under temperature stress than under normal temperatures, in line with the results of L. B. Bruno (2020b) [61]. An augmented plant biomass, stemming from nutrient accumulation, offers the most compelling evidence that PGPR promotes plant growth [62]. Therefore, PGPR can stimulate plant growth and enhance the plants' resistance to unfavorable conditions by intensifying physiological activity and regulating nutrient equilibrium [63]. This will provide favorable resources for the restoration of forage affected by extreme weather conditions in the Qinghai-Tibetan Plateau.

5. Conclusions

In this investigation, five distinct plant growth-promoting rhizobacteria (PGPR) strains (S1, S2, S3, S4, and S5) were isolated and characterized for their potent growth-enhancing capabilities. Subsequent pot experiments confirmed that these PGPR strains significantly bolstered the growth of *Elymus nutans* Griseb (*En*G), concurrently enhancing its nutrient utilization efficiency and cold tolerance. The presence of PGPR in the substrate notably elevated *En*G's plant height and dry weight, as well as its concentrations of soluble sugars and proteins, when compared to substrates devoid of PGPR. Furthermore, substrates amended with both PGPR and fertilizer demonstrated a marked increase in *En*G's apparent nutrient recovery efficiency, suggesting that PGPR strains are efficacious in optimizing plant nutrient uptake, thereby mitigating the necessity for excessive fertilizer application. These outcomes furnish valuable strain resources and foundational data for the formulation of specialized microbial fertilizers tailored for the alpine meadows of the Qinghai-Tibet Plateau.

Author contributions

All authors listed have made a substantial, direct, and intellectual contribution to the work and approved it for publication.

Funding

This work was financially supported by the Science and Technology Program of Tibet Autonomous Region (XZ202201ZY0005N), Sichuan Natural Science Foundation Project (2024NSFSC2074), National Natural Sciences Foundation of China (NO.41867013), China Forestry and Grassland Reform and Development Fund (GZFCG2023-17620), and Innovation Team Funds of China West Normal University (KCXTD2023-5). We also thank the anonymous reviewers for providing critical comments and suggestions that improved the manuscript.

Conflict of interest

The authors declare that the research was conducted in the absence of any commercial or financial relationships that could be construed as a potential conflict of interest.

Reference

1. Raina J-B, Eme L, Pollock FJ, Spang A, Archibald JM, Williams TA. Symbiosis in the microbial world: from ecology to genome evolution. Biol Open. 2018;7: bio032524. doi:10.1242/bio.032524

2. Smith D, Praslickova D, Ilangumaran G. Inter-organismal signaling and management of the phytomicrobiome. Frontiers in Plant Science. 2015;6. doi:10.3389/fpls.2015.00722

3. FAN X-H, ZHANG S-A, MO X-D, LI Y-C, FU Y-Q, Liu Z. Effect of PGPR and N Source on Plant Growth and N, P Uptake by Tomato Grown in Calcareous Soils. Pedosphere. 2017;27. doi:10.1016/S1002-0160(17)60379-5

4. Vessey JK. Plant growth promoting rhizobacteria as biofertilizers. Plant and Soil. 2003;255: 571–586. doi:10.1023/A:1026037216893

5. Santoyo G, Urtis-Flores CA, Loeza-Lara PD, Orozco-Mosqueda MDC, Glick BR. Rhizosphere Colonization Determinants by Plant Growth-Promoting Rhizobacteria (PGPR). Biology (Basel). 2021;10: 475. doi:10.3390/biology10060475

6. Wu SC, Cao ZH, Li ZG, Cheung KC, Wong MH. Effects of biofertilizer containing N-fixer, P and K solubilizers and AM fungi on maize growth: a greenhouse trial. Geoderma. 2005;125: 155–166. doi:10.1016/j.geoderma.2004.07.003

7. Elhaissoufi W, Ghoulam C, Barakat A, Zeroual Y, Bargaz A. Phosphate bacterial solubilization: A key rhizosphere driving force enabling higher P use efficiency and crop productivity. Journal of Advanced Research. 2022;38: 13–28. doi:10.1016/j.jare.2021.08.014

8. El-Tohamy W, El-Abagy H, El-Greadly N, Gruda N. Hormonal changes, growth and yield of tomato plants in response to chemical and bio-fertilization application in sandy soils. Journal of Applied Botany and Food Quality. 2008;82.

9. Zhang H, Zhu J, Gong Z, Zhu J-K. Abiotic stress responses in plants. Nat Rev Genet. 2022;23: 104–119. doi:10.1038/s41576-021-00413-0

10. Baum C. Interactive effects of plant growth-promoting rhizobacteria and organic fertilization on P nutrition of Zea mays L. and Brassica napus L. Journal of Plant Nutrition and Soil Science. 2011 [cited 12 Jul 2024]. Available: https://www.academia.edu/20876205/Interactive_effects_of_plant_growth_promoting_rhizobacteria_and_organic_fertilization_on_P_nutrition_of_Zea_mays_L_and_Brassica_napus_L

11. Jha Y, Mohamed HI. Inoculation with Lysinibacillus fusiformis Strain YJ4 and Lysinibacillus sphaericus Strain YJ5 Alleviates the Effects of Cold Stress in Maize Plants. Gesunde Pflanzen. 2023;75: 77–95. doi:10.1007/s10343-022-00666-7

12. de Souza EM, Lamb TI, Lamb TA, dos Santos Silva A, da Fré de Carvalho S, Nyland V, et al. Rhizospheric Soil from Rice Paddy Presents Isolable Bacteria Able to Induce Cold Tolerance in Rice Plants. J Soil Sci Plant Nutr. 2021;21: 1993–2006. doi:10.1007/s42729-021-00496-y

13. Askari-Khorasgani O, Hatterman-Valenti H, Flores F, Pessarakli M. Managing plant-environment-symbiont interactions to promote plant performance under low temperature stress. Journal of Plant Nutrition. 2019;42: 2010–2027. doi:10.1080/01904167.2019.1648682

14. Guo J, Bowatte S, Hou F. Diversity of endophytic bacteria and fungi in seeds of Elymus nutans growing in four locations of Qinghai Tibet Plateau, China. Plant Soil. 2021;459: 49–63. doi:10.1007/s11104-020-04608-y

15. Liu B, Wang R, Gong J, Zhu T, Long S, Guo H, et al. Combined Cold and Drought Stress-Induced Response of Photosynthesis and Osmotic Adjustment in Elymus nutans Griseb. Agronomy. 2023;13: 2368. doi:10.3390/agronomy13092368

16. Li M, Yang J, Wang X, Li D, Zhang C, Tian Z, et al. Transcriptome profiles identify the common responsive genes to drought stress in two *Elymus* species. Journal of Plant Physiology. 2020;250: 153183. doi:10.1016/j.jplph.2020.153183

17. Agarwal P, Singh PC, Chaudhry V, Shirke PA, Chakrabarty D, Farooqui A, et al. PGPR-induced OsASR6 improves plant growth and yield by altering root auxin sensitivity and the xylem structure in transgenic Arabidopsis thaliana. J Plant Physiol. 2019;240: 153010. doi:10.1016/j.jplph.2019.153010

18. Gul F, Khan IU, Rutherford S, Dai Z-C, Li G, Du D-L. Plant growth promoting rhizobacteria and biochar production from Parthenium hysterophorus enhance seed germination and productivity in barley under drought stress. Front Plant Sci. 2023;14. doi:10.3389/fpls.2023.1175097

19. Landa BB, Montes-Borrego M, Navas-Cortés JA. Use of PGPR for Controlling Soilborne Fungal Pathogens: Assessing the Factors Influencing Its Efficacy. In: Maheshwari DK, editor. Bacteria in Agrobiology: Disease Management. Berlin, Heidelberg: Springer; 2013. pp. 259–292. doi:10.1007/978-3-642-33639-3_10

20. Singh R, Jha P. The PGPR Stenotrophomonas maltophilia SBP-9 Augments Resistance against Biotic and Abiotic Stress in Wheat Plants. Frontiers in Microbiology. 2017;8: 1945. doi:10.3389/fmicb.2017.01945

21. Zhang X, Yang Z, Wang L, Yue Y, Wang L, Xiulian Yang. The effects of plant growth-promoting rhizobacteria on plants under temperature stress:A meta-analysis. Rhizosphere. 2023;28: 100788. doi:10.1016/j.rhisph.2023.100788

22. Galkiewicz JP, Kellogg CA. Cross-kingdom amplification using bacteria-specific primers: complications for studies of coral microbial ecology. Appl Environ Microbiol. 2008;74: 7828–7831. doi:10.1128/AEM.01303-08

23. Johnson JS, Spakowicz DJ, Hong B-Y, Petersen LM, Demkowicz P, Chen L, et al. Evaluation of 16S rRNA gene

sequencing for species and strain-level microbiome analysis. Nat Commun. 2019;10: 5029. doi:10.1038/s41467-019-13036-1

24. Hardy RWF, Holsten RD, Jackson EK, Burns RC. The Acetylene-Ethylene Assay for N_2 Fixation: Laboratory and Field Evaluation. Plant Physiology. 1968;43: 1185–1207.

25. Murphy J, Riley JP. A modified single solution method for the determination of phosphate in natural waters. Analytica Chimica Acta. 1962;27: 31–36. doi:10.1016/S0003-2670(00)88444-5

26. Nomura H, Komori T, Uemura S, Kanda Y, Shimotani K, Nakai K, et al. Chloroplast-mediated activation of plant immune signalling in Arabidopsis. Nat Commun. 2012;3: 926. doi:10.1038/ncomms1926

27. Wang M, Tachibana S, Murai Y, Li L, Lau SYL, Cao M, et al. Indole-3-Acetic Acid Produced by Burkholderia heleia Acts as a Phenylacetic Acid Antagonist to Disrupt Tropolone Biosynthesis in Burkholderia plantarii. Sci Rep. 2016;6: 22596. doi:10.1038/srep22596

28. Weselowski B, Nathoo N, Eastman AW, MacDonald J, Yuan Z-C. Isolation, identification and characterization of Paenibacillus polymyxa CR1 with potentials for biopesticide, biofertilization, biomass degradation and biofuel production. BMC Microbiol. 2016;16: 244. doi:10.1186/s12866-016-0860-y

29. Vijay S, Nair RR, Sharan D, Jakkala K, Ajitkumar P. Percoll discontinuous density gradient centrifugation method for the fractionation of the subpopulations of *Mycobacterium smegmatis* and *Mycobacterium tuberculosis* from *in vitro* cultures. MethodsX. 2023;11: 102344. doi:10.1016/j.mex.2023.102344

30. Zhao X, Zhong Y, Zhou W. Molecular mechanisms of mesocotyl elongation induced by brassinosteroid in maize under deep-seeding stress by RNA-sequencing, microstructure observation, and physiological metabolism. Genomics. 2021;113: 3565–3581. doi:10.1016/j.ygeno.2021.08.020

31. Xu F, Chu C, Xu Z. Effects of different fertilizer formulas on the growth of loquat rootstocks and stem lignification. Sci Rep. 2020;10: 1033. doi:10.1038/s41598-019-57270-5

32. Gurrieri L, Merico M, Trost P, Forlani G, Sparla F. Impact of Drought on Soluble Sugars and Free Proline Content in Selected Arabidopsis Mutants. Biology. 2020;9. doi:10.3390/biology9110367

33. Kielkopf CL, Bauer W, Urbatsch IL. Bradford Assay for Determining Protein Concentration. Cold Spring Harb Protoc. 2020;2020: 102269. doi:10.1101/pdb.prot102269

34. Isfan D. Nitrogen physiological efficiency index in some selected spring barley cultivars. Journal of Plant Nutrition. 1990;13: 907–914. doi:10.1080/01904169009364125

35. Novoa R, Loomis RS. Nitrogen and plant production. Plant and Soil. 1981;58: 177–204. doi:10.1007/BF02180053

36. Fageria NK, Baligar VC. Enhancing Nitrogen Use Efficiency in Crop Plants. Advances in Agronomy. Academic Press; 2005. pp. 97–185. doi:10.1016/S0065-2113(05)88004-6

37. Olasupo IO, Liang Q, Zhang C, Islam MS, Li Y, Yu X, et al. Agronomic Biofortification of Cayenne Pepper Cultivars with Plant Growth-Promoting Rhizobacteria and Chili Residue in a Chinese Solar Greenhouse. Microorganisms. 2021;9: 2398. doi:10.3390/microorganisms9112398

38. Aw X, Z L, Wc L, Zh Y. The effect of plant growth-promoting rhizobacteria (PGPR) on arsenic accumulation and the growth of rice plants (Oryza sativa L.). Chemosphere. 2020;242: 125136. doi:10.1016/j.chemosphere.2019.125136

39. Dhar Purkayastha G, Mangar P, Saha A, Saha D. Evaluation of the biocontrol efficacy of a Serratia marcescens strain indigenous to tea rhizosphere for the management of root rot disease in tea. PLoS One. 2018;13: e0191761. doi:10.1371/journal.pone.0191761

40. Ansari FA, Ahmad I. Fluorescent Pseudomonas -FAP2 and Bacillus licheniformis interact positively in biofilm mode enhancing plant growth and photosynthetic attributes. Sci Rep. 2019;9: 4547. doi:10.1038/s41598-019-40864-4

41. del Rosario Cappellari L, Santoro MV, Reinoso H, Travaglia C, Giordano W, Banchio E. Anatomical, morphological, and phytochemical effects of inoculation with plant growth- promoting rhizobacteria on peppermint (Mentha piperita). J Chem Ecol. 2015;41: 149–158. doi:10.1007/s10886-015-0549-y

42. Guan H, Liu X, Fu Y, Han X, Wang Y, Li Q, et al. The locoweed endophyte Alternaria oxytropis affects root development in Arabidopsis in vitro through auxin signaling and polar transport. J Exp Bot. 2023;74: 931–944. doi:10.1093/jxb/erac430

43. Hmaeid N, Wali M, Metoui-Ben Mahmoud O, Pueyo JJ, Ghnaya T, Abdelly C. Efficient rhizobacteria promote growth and alleviate NaCl-induced stress in the plant species *Sulla carnosa*. Applied Soil Ecology. 2019;133: 104–113. doi:10.1016/j.apsoil.2018.09.011

44. Akhtar N, Ilyas N, Meraj TA, Pour-Aboughadareh A, Sayyed RZ, Mashwani Z-U-R, et al. Improvement of Plant Responses by Nanobiofertilizer: A Step towards Sustainable Agriculture. Nanomaterials (Basel). 2022;12: 965. doi:10.3390/nano12060965

45. Fan H, Wu J, Liu W, Yuan Y, Hu L, Cai Q. Linkages of plant and soil C:N:P stoichiometry and their relationships to forest growth in subtropical plantations. Plant Soil. 2015;392: 127–138. doi:10.1007/s11104-015-2444-2

46. Mantelin S, Touraine B. Plant growth-promoting bacteria and nitrate availability: impacts on root development and nitrate uptake. Journal of Experimental Botany. 2004;55: 27–34. doi:10.1093/jxb/erh010

47. Ruiz Herrera LF, Shane MW, López-Bucio J. Nutritional regulation of root development. Wiley Interdiscip Rev Dev Biol. 2015;4: 431–443. doi:10.1002/wdev.183

48. Li X, Zeng R, Liao H. Improving crop nutrient efficiency through root architecture modifications. Journal of Integrative Plant Biology. 2016;58: 193–202. doi:10.1111/jipb.12434

49. Das PP, Singh KR, Nagpure G, Mansoori A, Singh RP, Ghazi IA, et al. Plant-soil-microbes: A tripartite interaction for nutrient acquisition and better plant growth for sustainable agricultural practices. Environ Res. 2022;214: 113821. doi:10.1016/j.envres.2022.113821

50. Egamberdiyeva D. The effect of plant growth promoting bacteria on growth and nutrient uptake of maize in two different soils. Applied Soil Ecology. 2007;36: 184–189. doi:10.1016/j.apsoil.2007.02.005

51. Krey T, Caus M, Baum C, Ruppel S, Eichler-Löbermann B. Interactive effects of plant growth–promoting rhizobacteria and organic fertilization on P nutrition of Zea mays L. and Brassica napus L. Journal of Plant Nutrition and Soil Science. 2011;174: 602–613. doi:10.1002/jpln.200900349

52. Wei T, Wang Y, Xie Z, Guo D, Chen C, Fan Q, et al. Enhanced ROS scavenging and sugar accumulation contribute to drought tolerance of naturally occurring autotetraploids in Poncirus trifoliata. Plant Biotechnol J. 2019;17: 1394–1407. doi:10.1111/pbi.13064

53. Selvarajan D, Mohan C, Dhandapani V, Nerkar G, Jayanarayanan AN, Vadakkancherry Mohanan M, et al. Differential gene expression profiling through transcriptome approach of Saccharum spontaneum L. under low

temperature stress reveals genes potentially involved in cold acclimation. 3 Biotech. 2018;8: 195. doi:10.1007/s13205-018-1194-2

54. Janská A, Marsík P, Zelenková S, Ovesná J. Cold stress and acclimation - what is important for metabolic adjustment? Plant Biol (Stuttg). 2010;12: 395–405. doi:10.1111/j.1438-8677.2009.00299.x

55. Batool T, Ali S, Seleiman MF, Naveed NH, Ali A, Ahmed K, et al. Plant growth promoting rhizobacteria alleviates drought stress in potato in response to suppressive oxidative stress and antioxidant enzymes activities. Sci Rep. 2020;10: 16975. doi:10.1038/s41598-020-73489-z

56. Bruno B, Karthik C, Ma Y, Kadirvelu K, Freitas H, Rajkumar M. Amelioration of chromium and heat stresses in Sorghum bicolor by Cr6+ reducing-thermotolerant plant growth promoting bacteria. Chemosphere. 2020;244: 125521. doi:10.1016/j.chemosphere.2019.125521

57. Patani A, Prajapati D, Ali D, Kalasariya H, Yadav VK, Tank J, et al. Evaluation of the growth-inducing efficacy of various Bacillus species on the salt-stressed tomato (Lycopersicon esculentum Mill.). Front Plant Sci. 2023;14. doi:10.3389/fpls.2023.1168155

58. Bisht SC, Joshi GK, Haque S, Mishra PK. Cryotolerance strategies of Pseudomonads isolated from the rhizosphere of Himalayan plants. Springerplus. 2013;2: 667. doi:10.1186/2193-1801-2-667

59. Theocharis A, Clément C, Barka EA. Physiological and molecular changes in plants grown at low temperatures. Planta. 2012;235: 1091–1105. doi:10.1007/s00425-012-1641-y

60. Croser J, Clarke H, Siddique K, Khan T. Low-Temperature Stress: Implications for Chickpea (Cicer arietinum L.) Improvement. Critical Reviews in Plant Sciences. 2003;22: 185–219. doi:10.1080/713610855

61. Bruno LB, Karthik C, Ma Y, Kadirvelu K, Freitas H, Rajkumar M. Amelioration of chromium and heat stresses in *Sorghum bicolor* by Cr6+ reducing-thermotolerant plant growth promoting bacteria. Chemosphere. 2020;244: 125521. doi:10.1016/j.chemosphere.2019.125521

62. Zhao X, Yuan X, Xing Y, Dao J, Zhao D, Li Y, et al. A meta-analysis on morphological, physiological and biochemical responses of plants with PGPR inoculation under drought stress. Plant, Cell & Environment. 2023;46: 199–214. doi:10.1111/pce.14466

63. Franco-Franklin V, Moreno-Riascos S, Ghneim-Herrera T. Are Endophytic Bacteria an Option for Increasing Heavy Metal Tolerance of Plants? A Meta-Analysis of the Effect Size. Front Environ Sci. 2021;8. doi:10.3389/fenvs.2020.603668

Table 1 Growth promoting characteristics of four PGPR strains

Strains	Nitrogen fixation capacity (μg mL^{-1})	Inorganic phosphorus solubilization capacity (μg mL^{-1})	Organic phosphorus solubilization capacity (μg mL^{-1})	Potassium-solubilising capacity (μg mL^{-1})	IAA contents (μg mL^{-1})
S1	5.10±0.07	176.2±62.46	24.73±15.91	4.42±0.34	596.87±63.7
S2	-	136.32±68.19	79.85±5.34	11.42±0.59	197.10±22.80
S3	2.08±0.06	250.24±30.11	44.52±26.51	8.18±0.42	193.32±7.17
S4	5.06±0.02	172.09±31.22	47.34±4.24	6.05±0.35	168.77±21.35
S5	5.57±0.03	311.95±50.06	49.46±5.61	4.59±0.44	166.64±16.88

Table 2 The main effects of temperature (T), PGPR, and fertilizers (NPK) on *Elymus nutans Griseb*. PT: plant height; DW: dry weight, Chl: plant chlorophyll; RL: root length; RB: number of root branches; RSA: root surface area; RD: root average diameter; SS: soluble sugars; SP: soluble proteins.

Source of variation	PT	DW	Chl	RL	RB	RSA	RD	SS	SP
**T**	<0.001	<0.001	<0.001	<0.001	0.076	<0.001	<0.001	<0.001	<0.001
**NPK**	<0.001	<0.001	<0.001	<0.001	<0.001	<0.001	0.847	<0.001	<0.001
**PGPR**	<0.001	<0.001	<0.001	<0.001	<0.001	0.007	<0.001	<0.001	<0.001
**T* NPK**	0.333	0.007	<0.001	0.429	0.893	0.012	0.015	<0.001	<0.001
**T* PGPR**	0.629	0.028	<0.001	<0.001	<0.001	0.392	<0.001	<0.001	<0.001
**NPK* PGPR**	0.386	0.007	<0.001	0.174	0.949	0.728	<0.001	<0.001	<0.001
**T* NPK* PGPR**	0.290	0.026	<0.001	0.020	0.898	0.010	0.181	<0.001	<0.001

Fig.1. General location of three sampling sites. Three randomly selected areas where *Elymus nutans Griseb* was growing vigorously.

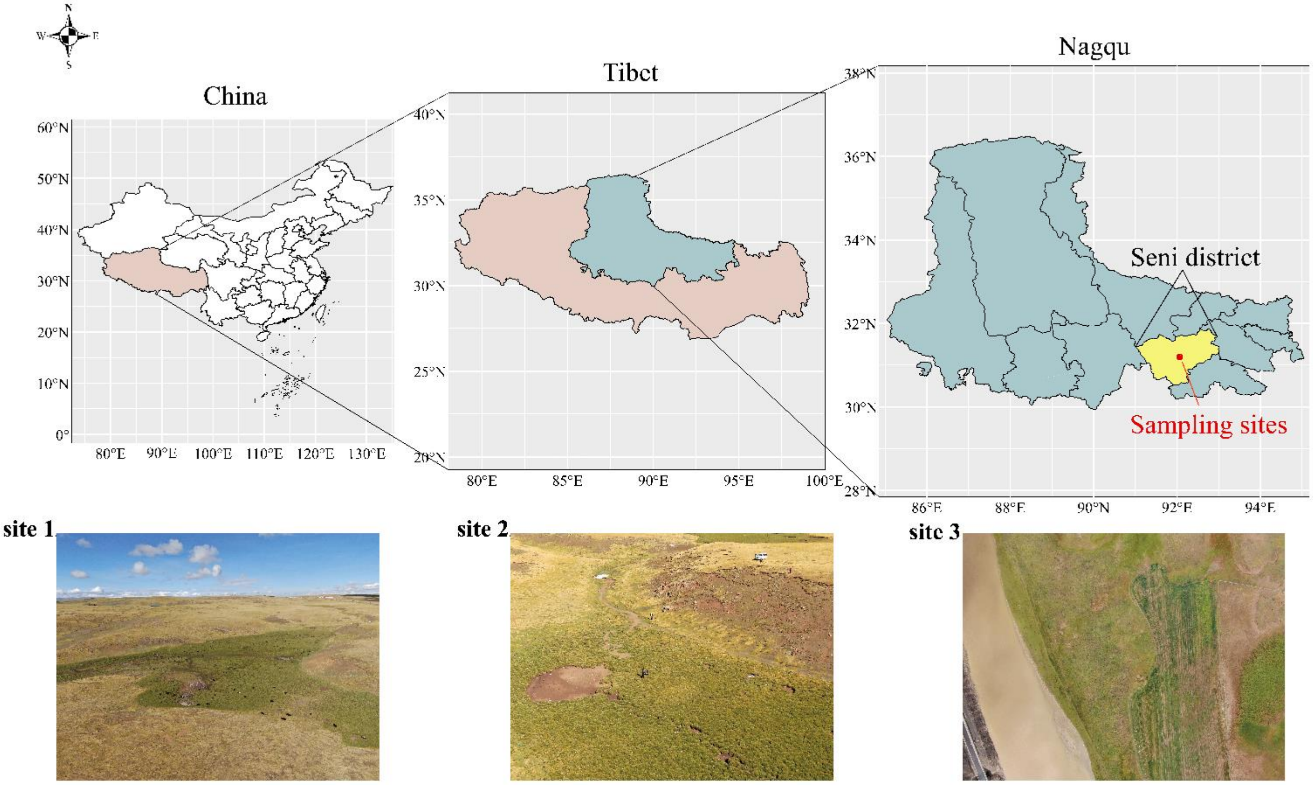

Fig.2. Effects of PGPR on *En*G at different temperatures. T1: PGPR in the substrate; T2: no PGPR in the substrate; T3: PGPR and fertilizer in the substrate; T4: fertilizer in the substrate but no PGPR.

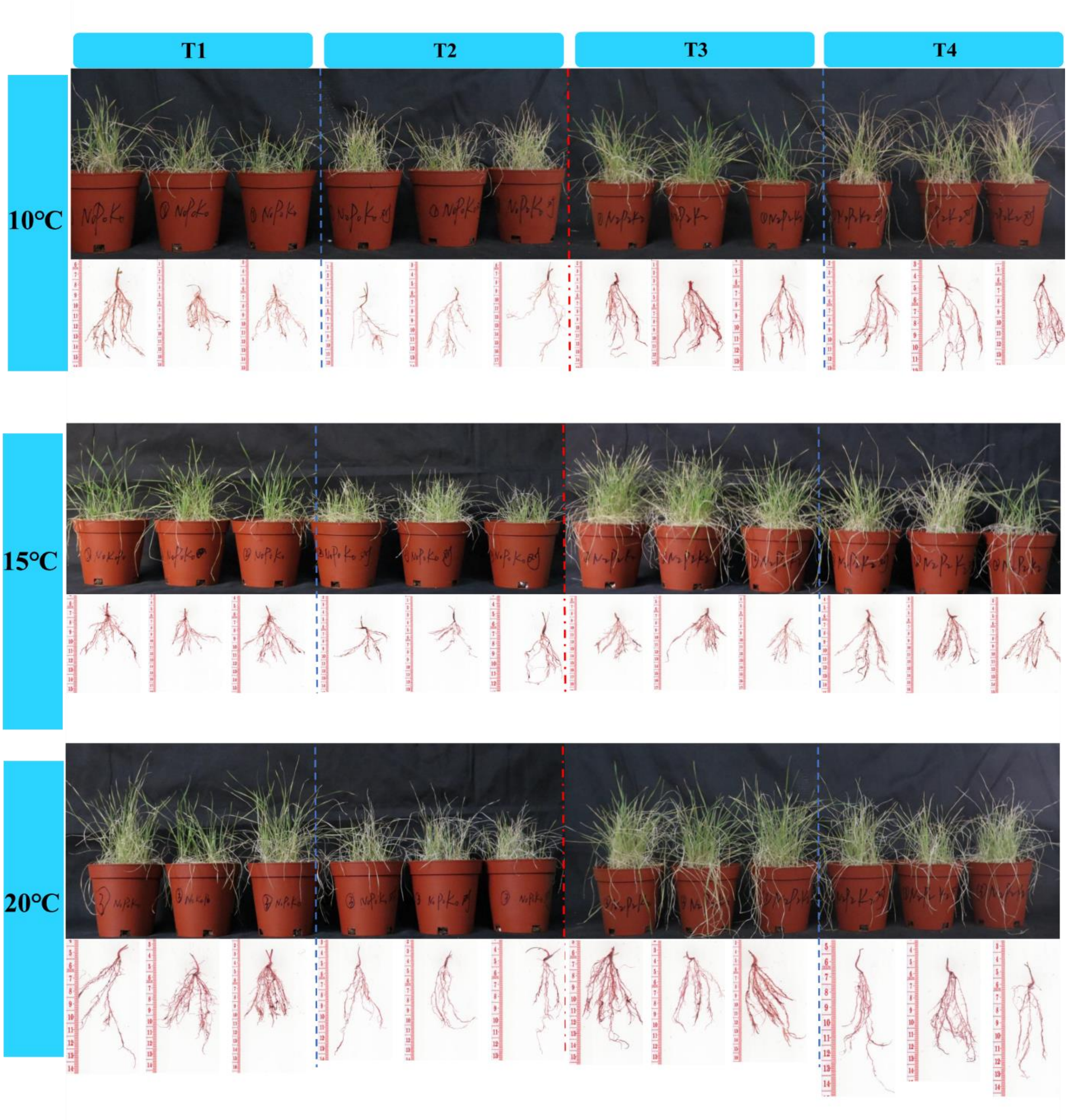

Fig.3. Effect of different treatments (PGPR in the substrate (T1), no PGPR in the substrate (T2), PGPR and fertilizer in the substrate (T3), and fertilizer in the substrate but no PGPR (T4)) on plant height (PT), dry weight (DW), and plant chlorophyll (Chl)content. Lowercase letters indicate differences between treatments and uppercase letters indicate differences between temperatures.

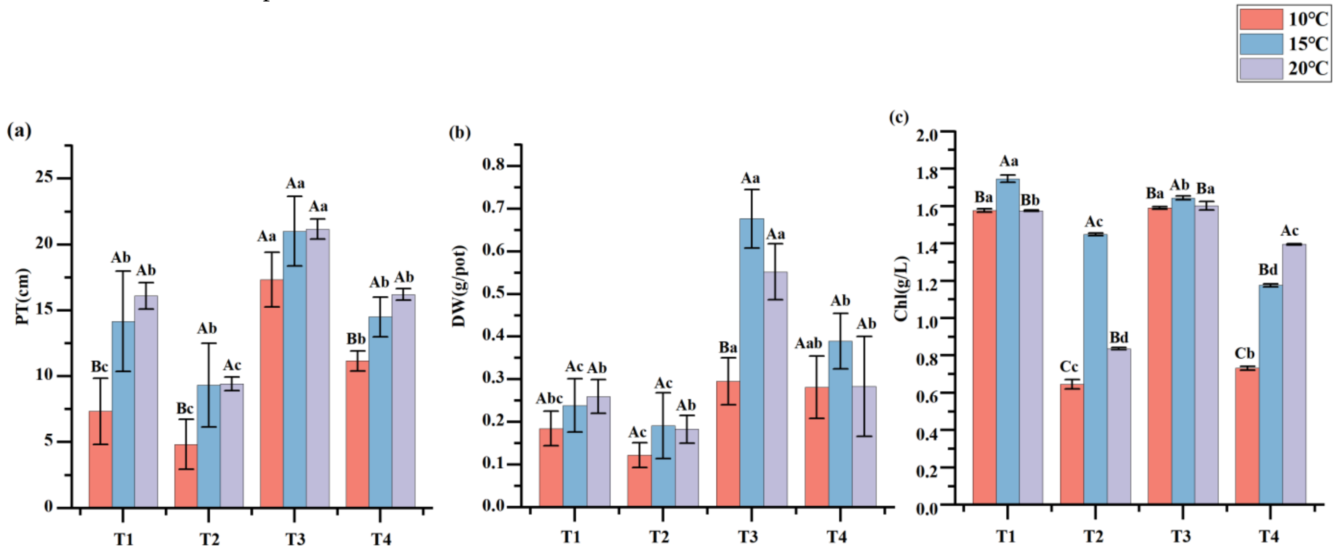

Fig.4. Effect of different treatments (PGPR in the substrate (T1), no PGPR in the substrate (T2), PGPR and fertilizer in the substrate (T3), and fertilizer in the substrate but no PGPR (T4)) on root length (RL), number of root branches (RB), root surface area (RSA), and root average diameter (RD). Lowercase letters indicate differences between treatments and uppercase letters indicate differences between temperatures.

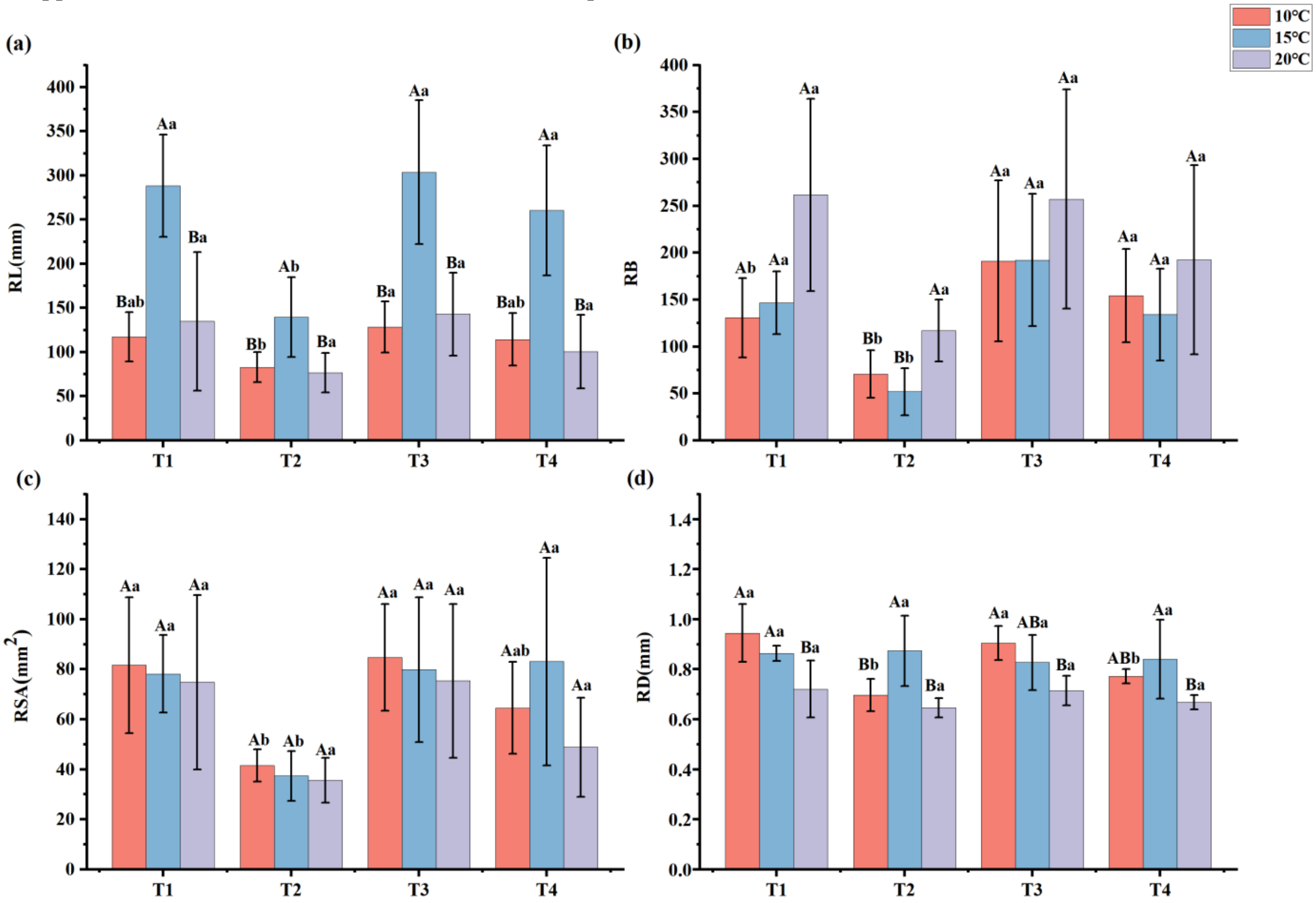

Fig.5. Effect of different treatments (PGPR in the substrate (T1), no PGPR in the substrate (T2), PGPR and fertilizer in the substrate (T3), and fertilizer in the substrate but no PGPR (T4)) on soluble sugars (SS), and soluble proteins (SP)content. Lowercase letters indicate differences between treatments and uppercase letters indicate differences between temperatures.

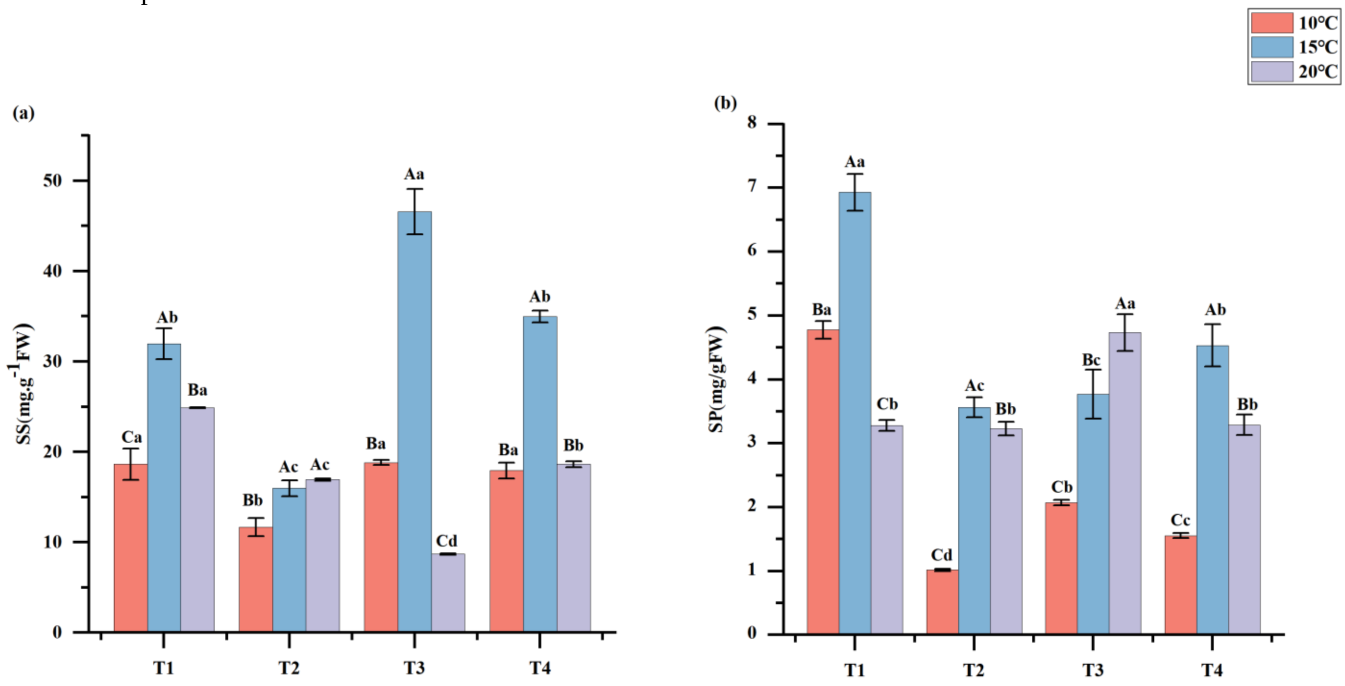

Fig.6. Effect of different treatments (PGPR and fertilizer in the substrate (T3) and fertilizer in the substrate but no PGPR (T4)) on physiological efficiency of nitrogen (NPE), phosphorus (PPE), potassium (KPE), agronomic efficiency of nitrogen (NAE), phosphorus (PAE) and potassium (KAE), and apparent recovery efficiency of nitrogen (NRE), phosphorus (PRE) and potassium (KRE) content. * means $p < 0.05$, ** means $p < 0.01$, *** means $p < 0.001$, ns means no significant difference.

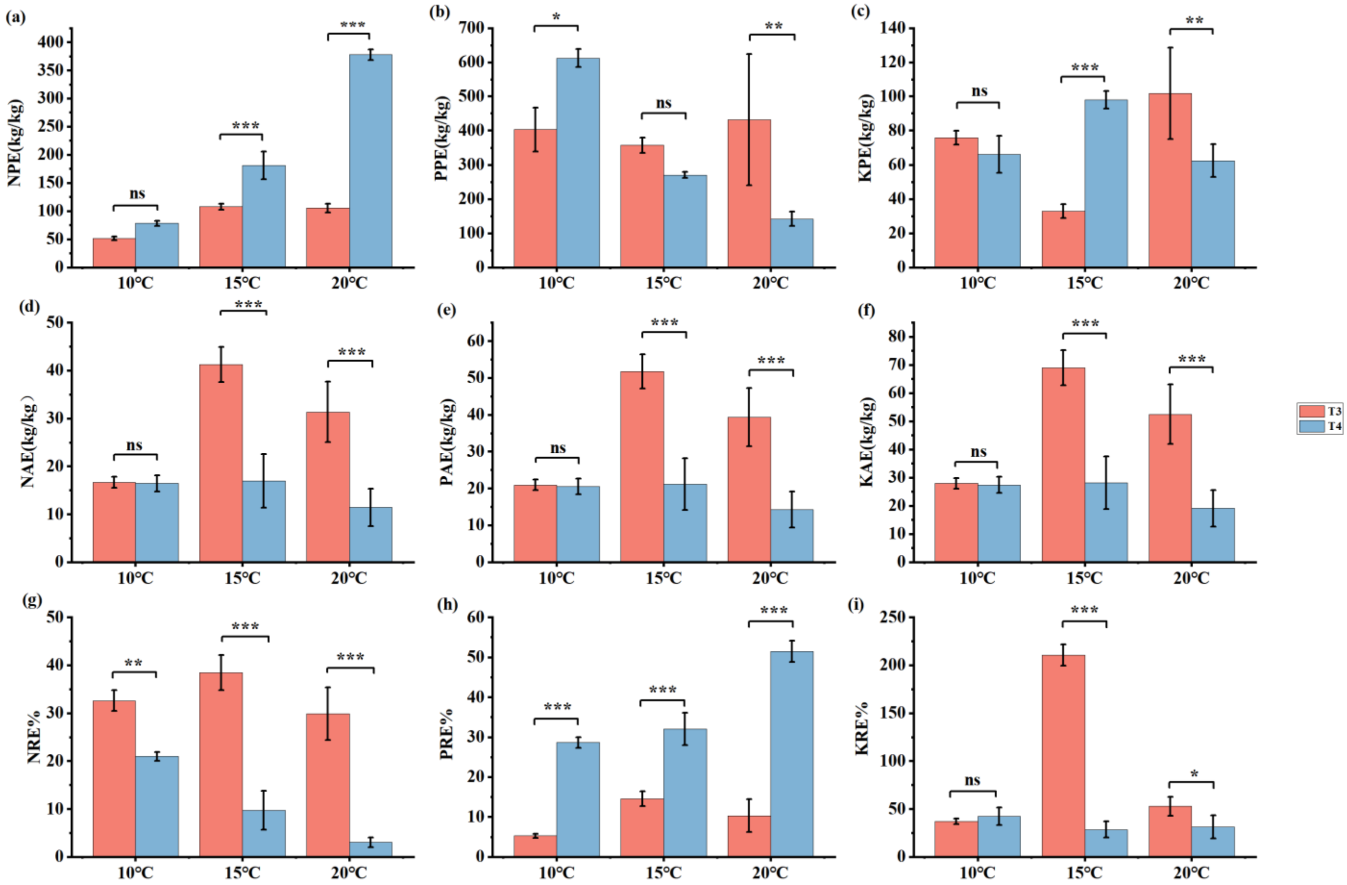

Fig.7. (a) Spearman's correlation between the relative abundance of PE, AE, and RE, and Plant growth factors. *, **, and *** represent significant correlations at the 0.05, 0.01, and 0.001 levels. (b) Mantel test between nutrient utilization and indicators of plant growth. PT, plant height; DW, dry weight; Chl, plant chlorophyll; RL, root length; RB, number of root branches; RSA, root surface area; RD, root average diameter; SS, soluble sugars; SP, soluble proteins.

(a) **(b)**

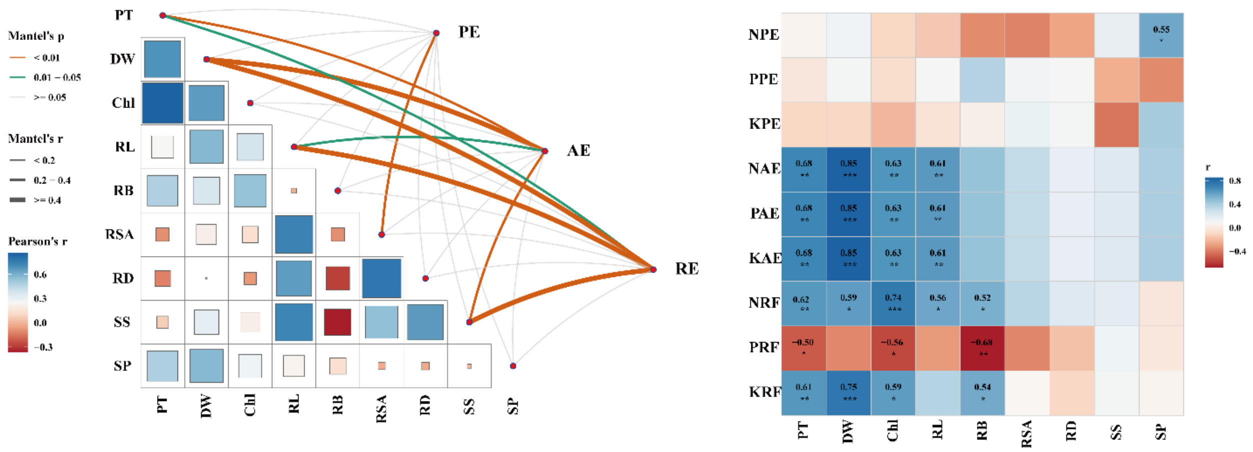

Supplement: Attachment — Submitted filename: PONE-D-24-56644.pdf [file pone.0323613.s001.pdf]
