# Supplementary material for: Effect of PGPR on growth and nutrient utilization of Elymus nutans Griseb at different temperatures
Source: PLoS One. 2025 May 12;20(5):e0323613. doi: 10.1371/journal.pone.0323613 (PMC12068589; doi:10.1371/journal.pone.0323613)
Supplement: Attachment — Submitted filename: PONE-D-24-56644_R1.pdf [file pone.0323613.s003.pdf]

PLOS ONE

Effect of PGPR on growth and nutrient utilization of Elymus nutans Griseb at different temperatures

--Manuscript Draft--

**Manuscript Number:**	PONE-D-24-56644R1
**Article Type:**	Research Article
**Full Title:**	Effect of PGPR on growth and nutrient utilization of Elymus nutans Griseb at different temperatures
**Short Title:**	Effect of PGPR on growth and nutrient utilization of Elymus nutans Griseb at different temperatures
**Corresponding Author:**	junqiang wang<br>China West Normal University<br>Wuwei, CHINA
**Keywords:**	Plant Growth-Promoting Rhizobacteria (PGPR); Nutrient Uptake; Temperature Effects; Elymus nutans Griseb; Resistance
**Abstract:**	In order to show the growth-promoting effects of PGPR (S1, S2, S3, S4 and S5) isolated from the rhizosphere soil of Elymus nutans Griseb (EnG) on the Tibetan Plateau. In this study, we simulated three temperature gradients (10°C, 15°C, and 20°C) during the growing season of Elymus nutans Griseb on the Tibetan Plateau, and set up four incubation substrate treatments:T1(addition of PGPR and no addition of NPK fertilizers), T2 (neither PGPR nor NPK fertilizers), T3 (addition of PGPR and NPK fertilizers), and T4 (no addition of PGPR, but addition of NPK fertilizers), to explore the effects of PGPR on the growth and nutrient (NPK) utilization of Elymus nutans Griseb at different temperatures. The results showed that compared with T2, the plant height (PT) of T1 increased by 51.72% to 70.67%, the dry weight (DW) increased by 24.99% to 51.25%, the soluble sugar (SS) and soluble protein (SP) content significantly increased by 59.37% and 369.66% at 10 °C respectively ($p<0.05$), and significantly increased by 100.17% and 94.5% at 15 °C respectively ($p<0.05$). Compared with T4, T3 significantly decreased the physiological efficiency of N (NPE) at 15 °C and 20 °C by 40.43% and 72.11%, respectively. In summary, our results showed that this PGPR can promote the growth of Elymus nutans Griseb and improve its nutrient utilization efficiency.
**Order of Authors:**	Linling Ran
	Haoyang Wu
	Fei Xia
	Yunyin Xue
	Wei Wei
	junqiang wang
	Jinglong Wang
	Shanshan Zhao
	Shuang Yan
	Hao Shi
	Shaikun Zheng
	Yu Zhang
	Xiaoqin Qiu
**Response to Reviewers:**	Dear Editor and Reviewers,<br>Thank you very much for your comments and professional advice. These opinions help to improve academic rigor of our article. Based on your suggestion and request we have made corrected modifications on the revised manuscript. We hope that our work

Powered by Editorial Manager® and ProduXion Manager® from Aries Systems Corporation

	can be improved again. We would like to show the details as File “Response to Reviewers”.
**Additional Information:**	
**Question**	**Response**
**Financial Disclosure**<br><br>Enter a financial disclosure statement that describes the sources of funding for the work included in this submission. Review the submission guidelines for detailed requirements. View published research articles from *PLOS ONE* for specific examples.<br><br>This statement is required for submission and **will appear in the published article** if the submission is accepted. Please make sure it is accurate.<br><br>**Funded studies**<br>Enter a statement with the following details:<br>• Initials of the authors who received each award<br>• Grant numbers awarded to each author<br>• The full name of each funder<br>• URL of each funder website<br>• Did the sponsors or funders play any role in the study design, data collection and analysis, decision to publish, or preparation of the manuscript?<br><br>Did you receive funding for this work?	Yes
Please add funding details.<br>as follow-up to "**Financial Disclosure**<br><br>Enter a financial disclosure statement that describes the sources of funding for the work included in this submission. Review the submission guidelines for detailed requirements. View published research articles from *PLOS ONE* for specific examples.<br><br>This statement is required for submission and **will appear in the published article** if the submission is accepted. Please make sure it is accurate.	This work was financially supported by the Science and Technology Program of Tibet Autonomous Region (XZ202201ZY0005N), Sichuan Natural Science Foundation Project (2024NSFSC2074), National Natural Sciences Foundation of China (NO.41867013), China Forestry and Grassland Reform and Development Fund (GZFCG2023-17620), and Innovation Team Funds of China West Normal University (KCXTD2023-5). We also thank the anonymous reviewers for providing critical comments and suggestions that improved the manuscript.<br><br>The funder actively participated in the study design, data gathering and analytical processes, as well as in making the decision regarding publication and manuscript compilation.

**Funded studies**<br>Enter a statement with the following details:<br>• Initials of the authors who received each award<br>• Grant numbers awarded to each author<br>• The full name of each funder<br>• URL of each funder website<br>• Did the sponsors or funders play any role in the study design, data collection and analysis, decision to publish, or preparation of the manuscript?<br><br>Did you receive funding for this work?"	
Please select the country of your main research funder (please select carefully as in some cases this is used in fee calculation).<br>as follow-up to "**Financial Disclosure**<br><br>Enter a financial disclosure statement that describes the sources of funding for the work included in this submission. Review the submission guidelines for detailed requirements. View published research articles from *PLOS ONE* for specific examples.<br><br>This statement is required for submission and **will appear in the published article** if the submission is accepted. Please make sure it is accurate.<br><br>**Funded studies**<br>Enter a statement with the following details:<br>• Initials of the authors who received each award<br>• Grant numbers awarded to each author<br>• The full name of each funder<br>• URL of each funder website<br>• Did the sponsors or funders play any role in the study design, data collection and analysis, decision to publish, or preparation of the manuscript?<br><br>Did you receive funding for this work?"	CHINA - CN

**Competing Interests**<br><br>Use the instructions below to enter a competing interest statement for this submission. On behalf of all authors, disclose any competing interests that could be perceived to bias this work—acknowledging all financial support and any other relevant financial or non-financial competing interests.<br><br>This statement is required for submission and **will appear in the published article** if the submission is accepted. Please make sure it is accurate and that any funding sources listed in your Funding Information later in the submission form are also declared in your Financial Disclosure statement.<br><br>View published research articles from *PLOS ONE* for specific examples.<br><br>**NO authors have competing interests**<br><br>Enter: *The authors have declared that no competing interests exist.*<br><br>**Authors with competing interests**<br><br>Enter competing interest details beginning with this statement:<br><br>*I have read the journal's policy and the authors of this manuscript have the following competing interests: [insert competing interests here]*<br><br>* typeset	The authors declare that the research was conducted in the absence of any commercial or financial relationships that could beconstrued as a potential conflict of interest.
**Ethics Statement**<br><br>Enter an ethics statement for this submission. This statement is required if the study involved:<br><br>• Human participants<br>• Human specimens or tissue<br>• Vertebrate animals or cephalopods	No.

Powered by Editorial Manager® and ProduXion Manager® from Aries Systems Corporation

• Vertebrate embryos or tissues<br>• Field research<br><br>Write "N/A" if the submission does not require an ethics statement.<br><br>General guidance is provided below. Consult the submission guidelines for detailed instructions. **Make sure that all information entered here is included in the Methods section of the manuscript.**	

**Format for specific study types**<br><br>**Human Subject Research (involving human participants and/or tissue)**<br>• Give the name of the institutional review board or ethics committee that approved the study<br>• Include the approval number and/or a statement indicating approval of this research<br>• Indicate the form of consent obtained (written/oral) or the reason that consent was not obtained (e.g. the data were analyzed anonymously)<br><br>**Animal Research (involving vertebrate animals, embryos or tissues)**<br>• Provide the name of the Institutional Animal Care and Use Committee (IACUC) or other relevant ethics board that reviewed the study protocol, and indicate whether they approved this research or granted a formal waiver of ethical approval<br>• Include an approval number if one was obtained<br>• If the study involved *non-human primates*, add *additional details* about animal welfare and steps taken to ameliorate suffering<br>• If anesthesia, euthanasia, or any kind of animal sacrifice is part of the study, include briefly which substances and/or methods were applied<br><br>**Field Research**<br><br>Include the following details if this study involves the collection of plant, animal, or other materials from a natural setting:<br>• Field permit number<br>• Name of the institution or relevant body that granted permission	
**Data Availability**<br><br>Authors are required to make all data underlying the findings described fully available, without restriction, and from the time of publication. PLOS allows rare exceptions to address legal and ethical concerns. See the PLOS Data Policy and FAQ for detailed information.	Yes - all data are fully available without restriction

A Data Availability Statement describing where the data can be found is required at submission. Your answers to this question constitute the Data Availability Statement and **will be published in the article**, if accepted.<br><br>**Important:** Stating 'data available on request from the author' is not sufficient. If your data are only available upon request, select 'No' for the first question and explain your exceptional situation in the text box.<br><br>Do the authors confirm that all data underlying the findings described in their manuscript are fully available without restriction?	
**Describe where the data may be found in full sentences. If you are copying our sample text, replace any instances of XXX with the appropriate details.**<br><br>• If the data are **held or will be held in a public repository**, include URLs, accession numbers or DOIs. If this information will only be available after acceptance, indicate this by ticking the box below. For example: *All XXX files are available from the XXX database (accession number(s) XXX, XXX.*).<br>• If the data are all contained **within the manuscript and/or Supporting Information files**, enter the following: *All relevant data are within the manuscript and its Supporting Information files.*<br>• If neither of these applies but you are able to provide **details of access elsewhere**, with or without limitations, please do so. For example:<br><br>*Data cannot be shared publicly because of [XXX]. Data are available from the XXX Institutional Data Access / Ethics Committee (contact via XXX) for researchers who meet the criteria for access to confidential data.*<br><br>*The data underlying the results presented in the study are available from (include the name of the third party*	Data used in this study can be found in Figshare (https://doi.org/10.6084/m9.figshare.27094078.v1).

and contact information or URL).<br>• This text is appropriate if the data are owned by a third party and authors do not have permission to share the data.<br><br>* typeset	
Additional data availability information:	

Manuscript Click here to access/download;Manuscript;Manuscript.docx

Effect of PGPR on growth and nutrient utilization of *Elymus nutans* Griseb at different temperatures

Linling Ran [a,1], Haoyang Wu [a], Fei Xia [b,c,1], Yunyin Xue [d], Wei Wei [b,c], Junqiang Wang [a,*], Jinglong Wang[b,c], Shanshan Zhao [a], Shuang Yan [a], Hao Shi[e], Shaikun Zheng[e], Yu Zhang [a], Xiaoqin Qiu [a]

[a] Key Laboratory of Southwest China Wildlife Resource Conservation (Ministry of Education). China West Normal University, Nanchong, 637009, China

[b] State Key Laboratory of Highland Barley and Yak Germplasm Resources and Genetic Improvement, Lhasa, 850000, China

[c] Institute of Pratacultural Science, Tibet Academy of Agriculture and Animal Husbandry Science, Lhasa, 850000, China

[d] School of Geography and Tourism, Shaanxi Normal University, Xi'an, 710119, China,

[e] College of Forestry, Gansu Agricultural University, Lanzhou, 730070, China

[*] Corresponding author at: Key Laboratory of Southwest China Wildlife Resource Conservation (Ministry of Education). China West Normal University, Nanchong,637009, China.

[1] These authors contributed equally to this work and should be considered co-first authors.

Tel: 0086/457/17390270491.

E-mail address: wangjunq0303@163.com (J.-Q. Wang).

Abstract

In order to show the growth-promoting effects of PGPR (S1, S2, S3, S4 and S5) isolated from the rhizosphere soil of *Elymus nutans* Griseb (*En*G) on the Tibetan Plateau. In this study, we simulated three temperature gradients (10°C, 15°C, and 20°C) during the growing season of *Elymus nutans* Griseb on the Tibetan Plateau, and set up four incubation substrate treatments:T1(addition of PGPR and no addition of NPK fertilizers), T2 (neither PGPR nor NPK fertilizers), T3 (addition of PGPR and NPK fertilizers), and T4 (no addition of PGPR, but addition of NPK fertilizers), to explore the effects of PGPR on the growth and nutrient (NPK) utilization of *Elymus nutans* Griseb at different temperatures. The results showed that compared with T2, the plant height (PT) of T1 increased by 51.72% to 70.67%, the dry weight (DW) increased by 24.99% to 51.25%, the soluble sugar (SS) and soluble protein (SP) content significantly increased by 59.37% and 369.66% at 10 °C respectively (p<0.05), and significantly increased by 100.17% and 94.5% at 15 °C respectively (p<0.05). Compared with T4, T3 significantly decreased the physiological efficiency of N (NPE) at 15 °C and 20 °C by 40.43% and 72.11%, respectively. In summary, our results showed that this PGPR can promote the growth of *Elymus nutans* Griseb and improve its nutrient utilization efficiency.

Keywords: Plant Growth-Promoting Rhizobacteria (PGPR); Nutrient Uptake; Temperature Effects; *Elymus nutans* Griseb; Resistance

1. Introduction

Under natural conditions, plants perpetually engage in intricate relationships with a microbial community known as the phytomicrobiome [1]. Certain soil microorganisms contribute to the enhancement of plant growth and progression [2]. Plant growth-promoting rhizobacteria (PGPR), a type

of beneficial bacteria found in nature, inhabit plant roots and enhance plant development [3]. PGPR encompasses numerous soil bacteria species that promote the growth of host plants when cultivated in their presence [4]. These microbes populate the root zone of plants and boost plant growth either directly or indirectly [5]. Wu [6] showed that the application of *Bacillus megaterium* and *Bacillus mucilaginous* as microbial inoculants enhanced plant growth and enhanced the plant's uptake of nutrients (total N, P, and K). These microbes play essential roles in the solubilization of P and K and nitrogen fixation. They transform insoluble P and K in the soil into plant-accessible forms via acidolysis, chelation, and exchange reactions, which in turn stimulate plant growth [7]. In addition, PGPR can regulate phytohormone levels in plants and thereby influence plant growth [8].

Plants face diverse environmental challenges that substantially hamper their growth and progression, potentially causing significant crop losses in farming. Cold temperatures represent a key non-biological element that restricts the growth and output of plants [9]. There is extensive documentation that suitable management techniques, encompassing the use of fertilizers and external protective compounds, can reduce the harmful influence of cold stress on plant growth [10]. PGPR can enhance plant nutrient uptake and accessibility via biochemical and physiological processes [11], and also enhance plant stress tolerance through systemic resistance induction, producing phytohormones, solubilizing nutrients [12,13]. Nevertheless, there are significant differences in temperature tolerance among different PGPR strains, and these differences directly affect their efficacy in plant growth [14]. Therefore, it is crucial to choose PGPR strains that are suitable for a particular forage and its environment.

Elymus nutans Griseb (*En*G) serves as a native and widespread grazing grass commonly found in the highland pastures of the Qinghai-Tibetan Plateau in China [15]. As an excellent pasture grass in the Qinghai-Tibetan Plateau region in China, it has strong resistance and tolerance to cold, rich nutrients, good palatability, high yield, and tolerance to desert areas among others. It can also prevent winds and fix sands,

conserve soil and water, and accelerate the process of ecological restoration of the grassland, which can effectively reduce the economic losses caused by natural disasters [16]. Thus, it is widely planted in the restoration of degraded alpine meadows on the Qinghai - Tibet Plateau [17].

The growth of *En*G relies on favorable relationships between its roots and microbes, which are essential for nutrient uptake and enhancing growth in its habitat [18]. Therefore, investigating the PGPR inhabiting the rhizosphere of *En*G on the Qinghai-Tibetan Plateau is crucial. Several studies demonstrate that PGPR can fix N, solubilize P, detoxify K, and secrete indole-3-acetic acid (IAA) to promote growth [19–22]. Nonetheless, few studies examined the effects of temperature on PGPR-mediated effects or PGPR inoculum colonization [23]. To systematically elucidate the growth-promoting mechanism of PGPR at different temperatures and to enhance the yield of *En*G, we aimed to: (1) investigate the effects of PGPR on the growth and nutrient utilization of *En*G and (2) on *En*G at different temperatures. The findings of this study help identify valuable microbial resources and provide foundational insights, thus supporting the development and application of PGPR as a biofertilizer to enhance pasture productivity on the Tibetan Plateau.

2. Materials and methods

2.1. Sources of PGPR

The sampling area is located in Nagqhu Town, Seni District, Nagqhu City, Tibet Autonomous Region, with geographic coordinates of 31°26′N latitude and 92°07′E longitude (Fig. 1), and an average elevation of 4450 m. Three sampling sites with good growth and disease-free *En*G were randomly selected, and the rhizosphere soil of the *En*G was collected using root shaking method, with five replicate mixes randomly sampled at each site. And the rhizosphere soil, together with the fine roots, were put into sterile self-sealing bags and stored at low temperature (4℃) to be brought back to the laboratory. The collected soil samples

were prepared into 10^{-4} and 10^{-5} soil suspensions and 100 μL of soil suspension was aspirated and spread evenly on beef paste peptone agar plates for bacterial purification and isolation of bacteria.

2.2. Determination of the growth-promoting ability of PGPR and screening of high-quality bacterial strains

Inoculate the purified strains onto Nitrogen free medium (NFM), Pikovskaya medium, Bacterial Organophosphorus medium, Potassium Bacteria medium and King's B medium respectively to determine whether the strains have growth - promoting abilities. Among them, 273 strains can grow on the selective medium. The ability of the isolates to grow on NFM agar and their nitrogen-fixing capacity was evaluated using the acetylene reduction method [24]. Strains' phosphorus solubilizing capacity was tested with Mo blue colorimetry [25]. The strains were cultured in 50 mL King's broth at 28 °C with shaking (180 rpm) for 3 days. IAA was extracted from the culture filtrate using ethyl acetate, with each extraction done three times [26]. The IAA content was measured using high-performance liquid chromatography [27]. The KSB-containing solution was diluted to 50 mL and centrifuged at 500 rpm for 10 minutes to eliminate solids. Subsequently, a 10 mL aliquot was further centrifuged at 10,000 rpm for 10 minutes, and the K in the supernatant was quantified using flame spectrophotometry [28]. Five excellent strains (S1, S2, S3, S4 and S5) with high growth promoting ability were screened by the determination of growth-promoting ability. All five strains solubilized inorganic and organic phosphates, decomposed K, and secreted IAA. In addition, S1, S3, S4, and S5 fixed N (Table 1).

2.3. Identification of PGPR strains

Five strains (S1, S2, S3, S4, and S5) were inoculated in LB liquid medium for incubation at 37 °C under shaking (160 rpm) for 24 h. Bacterial genomic DNA was extracted from these strains using a commercial kit (Tiangen Biochemical Technology, Beijing, China). The 16S rRNA gene was polymerase chain reaction (PCR) amplified with the primer set 27 F (5′-AGAGTTTGATCCTGGCTCAG-3′) and

1492R (5′-GGTTACCTTGTTACGACTT-3′) as described by Galkiewicz and Kellogg (2008) [29]. PCR was executed in a 50 μL volume, comprising 1 μL of 5 μM forward primer, 1 μL of 5 μM reverse primer, 25 μL of 2×Taq PCR MasterMix, 2 μL of DNA template, and double-distilled water. The PCR thermal cycling parameters were set to an initial denaturation at 95 °C for 5 minutes, followed by 30 cycles of 95 °C for 30 seconds, 57 °C for 60 seconds, and 72 °C for 30 seconds, with a concluding extension phase at 72 °C for 10 minutes. The PCR results were validated through 1% agarose gel electrophoresis and subsequently subjected to sequencing via the dideoxy chain-termination approach at Sangon Biotech Co., Ltd. (Shanghai, China) [30]. The sequences of the 16S rRNA genes from the five bacterial strains have been submitted and archived in the GenBank repository, and corresponding accession numbers have been assigned: PQ351188, PQ351189, PQ351190, PQ351191, and PQ351192.

2.4 Mixed bacterial solution and incubation substrate preparation

The compatibility between excellent strains (S1-S5) was verified by the plate confrontation method [31]. The excellent strains (S1-S5) were inoculated into sterilized LB liquid medium respectively, and activated and cultured in a constant temperature shaker at 150 r/min and 30 °C for 12 h. The OD_{600} value of each activated bacterial solution was adjusted to 0.6. And then transferred to the fresh LB medium, 150r/min, 30°C constant temperature shaker to expand the culture for 16h, after doubling the bacterial solution after centrifugation to remove the supernatant, to the bacterial body of the re-suspension of sterile water, fully mixed and made into a single bacterial agent. After sufficient mixing and make the single bacterial agent with OD_{600} value of 0.6 (the concentration of the single bacterial agent is about 1×10^8CFU/ml). Equal amount of mixing to prepare a composite bacterial agent [32].

The culture substrate was formulated with reference to the soil characteristics of the sampling sites, which was composed of a blend of peat soil, vermiculite, perlite, and coir in a ratio of 4:1:1:1 (v/v), with

50 g per bag, which was then sterilized at 121 °C for 30 minutes. Subsequently, 78.2 g of the culture medium was combined with 1% (volume/volume) PGPR bacterial mixture and transferred into a 660 mL plastic container (70 × 95 × 98 mm) [33]. A cup with an equal volume of sterile water was used as the control.

2.5 Incubation experiment

The four treatments set up in this study were T1(addition of PGPR and no addition of NPK fertilizers), T2 (neither PGPR nor NPK fertilizers), T3 (addition of PGPR and NPK fertilizers), and T4 (no addition of PGPR, but addition of NPK fertilizers) at three replicates. The NPK represents applied rates of 150 mg kg^{-1}, 120 mg kg^{-1}, and 90 mg kg^{-1} of urea (N,46.65%), superphosphate (P_2O_5,12%) and potassiumchloride (K_2O,60%), respectively. Furthermore, according to the climatic temperature during the growing season (June, July, and August) of *En*G on the Tibetan Plateau (Fig. 1), this study was set up with temperature gradients of 10 °C, 15 °C, and 20 °C. Incubation was carried out in three incubators with a light (16 h)/dark (8 h) cycle (light at 350 μEm^{-2} s^{-1}) and 60% humidity. Plant seeds (wild *En*G seeds collected in Nagqhu, Tibet) were sterilized on the surface with a 1:1 mixture of 30% hydrogen peroxide and 70% ethanol for 10 minutes and cleaned repeatedly with demineralized sterile water. Thirty-five *En*G seeds were planted in each cup. In order to better simulate the plant growth cycle in alpine meadows, plant growth indicators were measured after 120 days of plant growth (based on the local plant growth season).

2.6 Indicators measurement

2.6.1 Dry weight (DW), plant height (PT), and root system assay

A random sample of 15 plants per pot was taken to determine plant height (PT). After drying at 65 °C for 24 h, the dry weight (DW) was noted. Root length (RL), the count of root branches (RB), root surface area (RSA), and the average root diameter (RD) were all assessed using an LA2400 root scanner (Jeili

Electronics Trading Co., Ltd, Shanghai, China) [34].

2.6.2 Plant chlorophyll (Chl), soluble sugars (SS), and soluble proteins (SP) content assay

To measure the chlorophyll (Chl) content, 0.1 g of fresh leaf tissue from each seedling was mixed with a bit of quartz sand, ground in 80% acetone, and then centrifuged at 9000 × g and 4 °C for 10 minutes. The supernatant's absorbance was read at 440, 663, and 645 nm to assess Chl a, b, and carotenoid levels, respectively. The total Chl content was computed using Li [18]. The soluble sugar (SS) content was assessed following the method of [35]. Soluble protein (SP) content was determined using Bradford's method for staining with Caulmer Brilliant Blue [36].

2.6.3 Plant nutrient use efficiency

The physiological efficiency (PE) of N (NPE), P (PPE), and K (KPE), i.e., the kg plant yield N, P, and K uptake, was calculated according to [37]. The agronomic efficiencies (AE) of N (NAE), P (PAE), and K (KAE), i.e., the yield increase for each kg N, P, K applied, was calculated according to Novoa and Loomis [39]. The apparent recovery efficiency (RE) of N (NRE), P (PRE), and K (KRE), i.e., plant N, P, and K uptake (kg ha^{-1}) per kg of N, P, and K applied, was calculated according to Fageria and Baligar [40].

2.7 Statistical analyses

Statistical analyses were performed using SPSS software (version 27.0; SPSS Inc., Chicago, IL, USA). In the context of our research, prior to conducting parametric tests on each dataset, we initiated a preliminary assessment using the Shapiro-Wilk test to ascertain the conformity of the data to a normal distribution pattern. Mean differences were determined using the least significant difference method and the Tukey test at a significance level of 5%. Statistical significance is reported at $p < 0.05$. All visualizations were produced using R 4.4.1. The relationship between the growth indicators of *En*G and nutrient utilization was analyzed through Mantel analysis.

3. Result

*3.1 Effects of PGPR on En*G *growth characteristics at different temperatures*

Substrate with PGPR microbial inoculants influenced the growth characteristics of *En*G (Fig.2). Compared to T2, treatment T1 increased PT by 51.72% (10 ℃) to 70.67% (20 ℃), and increased DW, accounting for 24.99% (15 ℃) to 51.25% (10 ℃) (Fig.3). Compared to T4, treatment T3 significantly increased PT by 30.66% (20 ℃) to 55.22% (10 ℃), and significantly increased DW, accounting for 4.86 % (10 ℃) to 94.71% (20 ℃) (p<0.05) (Fig.3a and 3c). Treatments T1 and T3 significantly increased the Chl of *EnG* compared to T2 and T4 (p<0.05) (Fig.3c).

*3.2 Effects of PGPR on En*G *root growth at different temperatures*

The effects of PGPR on the roots of *En*G are shown in Fig.4. Compared to T2, the T1 significantly increased RSA of *En*G by 96.32% and 109.31% at 10 °C and 15 °C, respectively (p<0.05) (Fig.4). Compared with the T4 treatment, the T3 treatment increased the RL, RB, RSA, and RD of *En*G. Furthermore, the RL of *En*G was significantly higher at 15 °C than at 20 °C (p<0.05) (Fig.4a).

*3.3 Effects of PGPR on SS and SP of En*G *at different temperatures*

Compared to treatment T2, T1 significantly increased the SS and SP of *En*G by 59.37% and 369.66% at 10 °C, by 100.17% and 94.5% at 15 °C, respectively (p<0.05) (Fig.5). Furthermore, the content of SS at 10 °C under the T3 treatment was significantly higher than that of SS at 20 °C (Fig. 5a). The contents of SS and SP at 10 °C under T1 were significantly higher than at 20 °C (p< 0.05) (Fig.5b).

*3.4 Effects of PGPR on En*G *nutrients use efficiency indexes at different temperatures*

Compared with treatment T4, T3 significantly decreased NPE of *En*G at 15°C and 20°C by 40.43% and 72.11%, respectively (p<0.001) (Fig.6a). However, compared with T4, T3 significantly decreased the PRE of *En*G (p<0.001) (Fig.6h). Furthermore, the T3 treatment significantly increased the NAE, PAE,

KAE, NRE, and KRE of *En*G at 15°C and 20°C ($p<0.001$) (Fig.6).

3.5 Relationship between PGPR and growth indicators of EnG

The PGPR significantly affected PT, DW, Chl, RL, RSA, RD, SS, and SP (Table 2). The interaction between temperature and PGPR significantly affected the RL of *En*G (Table 2) ($p<0.001$). In addition, RL was significantly and positively correlated with AE (Fig.7b) ($p<0.05$). PT and DW were highly significantly correlated with NAE, PAE, and KAE (Fig. 7a) ($p<0.001$), and PT and DW were significantly correlated with the apparent NRE, PRE, and KRE (Fig.7a) ($p<0.05$). In addition, RL was significantly and positively correlated with AE (Fig.7) ($p<0.05$).

4. Discussion

*4.1 The effects of PGPR on En*G *growth*

The plant microbiome has been recognized as a key plant characteristic influencing crop yield. Olasupo et al. [41] applied various bacillus treatments to peppers, resulting in a 27%–36% boost in biomass. Similarly, Aw et al. [42] isolated three As-resistant PGPR strains to verify their roles in promoting rice growth. In the present study, compared to the T2 treatment, the T1 treatment increased the PT and DW of *En*G (Fig.3). These findings corroborated the efficacy of PGPR in augmenting the growth of *En*G. Furthermore, the T3 treatment significantly increased the PT and DW of *En*G compared with T4 ($p<0.05$) (Fig.3a and 3c). There was also a significant correlation between PT, DW, AE, and RE (Fig.7a) ($p<0.05$). This correlation underscores the role of the culture substrate with PGPR in facilitating *En*G nutrient uptake. Consequently, the application of PGPR can improve soil nutrient use effectively and reduce the amount of chemical fertilizer applied and the negative effects of excessive fertilization [42], thereby, *En*G biomass can be increased, and economic and ecological development can be achieved.

Plant growth requires the accumulation of photochemical products and is directly linked to the

photosynthetic rate [43]. del Rosario Cappellari [45] evaluated the growth characteristics and Chl levels in peppermint (*Mentha piperita*) seedlings exposed to PGPR strains, noting that the treated plants demonstrated improved values for these metrics compared to the untreated controls. Consistent with these findings, our T1 treatment significantly increased the Chl content of *En*G compared with T2 (p<0.05) (Fig.3c). This indicates that the culture substrate with PGPR had a certain promoting effect on the photosynthesis of *En*G.

Furthermore, we found that treatment T1 significantly increased the RSA of *En*G at 15 °C by 109.31% compared with treatment T2 (p<0.05) (Fig.4). Earlier research implied that the impact of PGPR on root growth is connected to the synthesis of IAA, a substance known to advance root expansion and enhancement, augment the area of the root surface, and stimulate root metabolic processes [19]. Consistent with these findings, the culture substrate containing PGPR elicited IAA production (Table 1). The expansion of the root system surface area augments the nutrient absorption area, which is beneficial for facilitating the uptake of nutrients and water [45]. It is worth noting that there was a significant correlation between RSA levels and PE (Fig.7) (p<0.01). This indicates that the PGPR is capable of enhancing the nutrient uptake efficiency of *En*G by promoting root growth [46].

*4.2 The effect of PGPR on the nutrient utilization of En*G

N, P, and K are fundamental chemical elements essential for plant development, contributing significantly to plant tissue formation and physiological processes [47]. The effect of PGPR on plant nutrition stems from its impact on nutrient absorption by plants [48]. Wu et al. [6] found that Bacillus megaterium and Bacillus mucilaginous inoculants not only boosted plant growth but also enhanced the plants' nutrient absorption capabilities, particularly in terms of total N, P, and K. In this study, the T3 treatment significantly increased the NAE, PAE, and KAE of *En*G at 15°C and 20°C. This indicates that the PGPR is capable of enhancing the nutrient uptake efficiency of *En*G, reducing the dependence on

chemical fertilizers, and thereby promoting sustainable agricultural development [49]. In addition, the root system of plants performs several functions, including nutrient acquisition, water uptake, stability, and symbiotic relationships with beneficial soil microorganisms, which collectively enhance nutrient absorption efficiency [50]. In the present study, the AE and RE were significantly correlated with RL (Fig. 7a). In addition, NAE, PAE, KAE, and NRE were significantly positively correlated with RL (Fig.7b).These findings affirm that efficient nutrient uptake also relies significantly on the root system's capacity to permeate the soil [51].

Compared with treatment T4, T3 significantly decreased the NPE of *En*G at 20 °C by 72.11% ($p<0.001$) (Fig. 6a). This may be due to the fact that PGPR transform unavailable nutrient forms for plants into available forms [52], which reduces the plants' need for fertilizer. PGPR plays a significant role in agriculture by facilitating the circulation of plant nutrients and reducing the reliance on chemical fertilizers [53]. Our findings offer microbial resources and foundational data for the development and application of PGPR as biofertilizers; however, compared witht T4, the T3 treatment significantly decreased the PRE of *En*G ($p<0.001$) (Fig.6h). This aligns with previous research indicating that the use of PGPR can enhance P mobilization and supply to crops in soils deficient in P [54]. Therefore, we propose that the culture substrate with PGPR promotes nutrient uptake by *En*G and can help reduce the use of chemical fertilizers in agriculture.

4.3 The effects of PGPR at different temperatures

Under low-temperature stress conditions, plants exhibit a heightened accumulation of SS such as sucrose, glucose, fructose, ribose, and trehalose [55]. These sugars are capable of neutralizing free radicals and indirectly stimulating protein synthesis, which in turn enhances cold resistance [56]. SP are also pivotal, serving as osmoregulators and supporting low-temperature tolerance in plants [57]. Previous studies show that the contents of SP increased up to 68% in plants treated with PGPR [58]. Additionally,

the SS content was significantly elevated in PGPR-treated plants in a previous study, and it was approximately double that of the untreated controls [55]. Our findings corroborate these observations as compared with treatment T2, T1 significantly increased the SS and SP content of *En*G by 59.37% and 369.66% at 10 °C (Fig. 5). This suggests that the physiological and biochemical status of *En*G significantly improved under the influence of PGPR [59]. Thus, inoculating plants with PGPR can be a productive method to foster growth by enhancing their tolerance to various abiotic stresses [60]. It is worth noting that the content of SS and SP under the T1 and T3 treatment was significantly higher at 10 °C than at 20 °C ($p < 0.05$) (Fig. 5b). This is because low temperatures may induce changes in the activity of enzymes related to the synthesis of soluble sugars (SS). At 10 °C, these enzymes may be more inclined to break down and convert stored complex carbohydrates, such as starch, into soluble sugars. This is due to the fact that upon sensing the low - temperature environment, plants initiate a series of physiological adaptation mechanisms. They transform less mobile nutrient forms, like starch, into soluble sugars that are more easily transported and utilized, so as to maintain the osmotic balance and energy supply of cells [61,62].

Temperature plays a key role in restricting the distribution and output of plants [63]. It is a critical environmental factor that profoundly influences plant root growth, shaping root system architecture and influencing nutrient uptake and overall plant health [64]. In this study, the interaction between temperature and the application of PGPR inoculum significantly affected the root length of *En*G ($p<0.001$) (Table 2). This can be attributed to the combined effects of temperature on root development and the ability of PGPR to modulate plant hormone levels and improve nutrient and water uptake [65]. Consequently, the RL of *En*G was significantly higher at 15 °C than at 20 °C ($p<0.05$) (Fig.4a). However, under the influence of PGPR, the *En*G was more productive after inoculation with PGPR under temperature stress than under normal temperatures, in line with the results of L. B. Bruno [67]. An augmented plant biomass, stemming from nutrient accumulation, offers the most compelling evidence that PGPR promotes plant growth [67].

Therefore, PGPR can stimulate plant growth and enhance the plants' resistance to unfavorable conditions by intensifying physiological activity and regulating nutrient equilibrium [68]. This will provide favorable resources for the restoration of forage affected by extreme weather conditions in the Qinghai-Tibetan Plateau.

5. Conclusions

In this investigation, five distinct plant growth-promoting rhizobacteria (PGPR) strains (S1, S2, S3, S4, and S5) were isolated and characterized for their potent growth-enhancing capabilities. Subsequent pot experiments confirmed that these PGPR strains significantly bolstered the growth of *Elymus nutans* Griseb (*En*G), concurrently enhancing its nutrient utilization efficiency and cold tolerance. The presence of PGPR in the substrate notably elevated *En*G's plant height and dry weight, as well as its concentrations of soluble sugars and proteins, when compared to substrates devoid of PGPR. Furthermore, substrates amended with both PGPR and fertilizer demonstrated a marked increase in *En*G's apparent nutrient recovery efficiency, suggesting that PGPR strains are efficacious in optimizing plant nutrient uptake, thereby mitigating the necessity for excessive fertilizer application. These outcomes furnish valuable strain resources and foundational data for the formulation of specialized microbial fertilizers tailored for the alpine meadows of the Qinghai-Tibet Plateau.

Author contributions

All authors listed have made a substantial, direct, and intellectual contribution to the work and approved it for publication.

Funding

This work was financially supported by the Science and Technology Program of Tibet Autonomous Region (XZ202201ZY0005N), Sichuan Natural Science Foundation Project (2024NSFSC2074), China

Forestry and Grassland Reform and Development Fund (GZFCG2023-17620), and Innovation Team Funds of China West Normal University (KCXTD2023-5). We also thank the anonymous reviewers for providing critical comments and suggestions that improved the manuscript.

Conflict of interest

The authors declare that the research was conducted in the absence of any commercial or financial relationships that could be construed as a potential conflict of interest.

Reference

1. Raina J-B, Eme L, Pollock FJ, Spang A, Archibald JM, Williams TA. Symbiosis in the microbial world: from ecology to genome evolution. Biol Open. 2018;7: bio032524. doi:10.1242/bio.032524
2. Smith D, Praslickova D, Ilangumaran G. Inter-organismal signaling and management of the phytomicrobiome. Frontiers in Plant Science. 2015;6. doi:10.3389/fpls.2015.00722
3. FAN X-H, ZHANG S-A, MO X-D, LI Y-C, FU Y-Q, Liu Z. Effect of PGPR and N Source on Plant Growth and N, P Uptake by Tomato Grown in Calcareous Soils. Pedosphere. 2017;27. doi:10.1016/S1002-0160(17)60379-5
4. Vessey JK. Plant growth promoting rhizobacteria as biofertilizers. Plant and Soil. 2003;255: 571–586. doi:10.1023/A:1026037216893
5. Santoyo G, Urtis-Flores CA, Loeza-Lara PD, Orozco-Mosqueda MDC, Glick BR. Rhizosphere Colonization Determinants by Plant Growth-Promoting Rhizobacteria (PGPR). Biology (Basel). 2021;10: 475. doi:10.3390/biology10060475
6. Wu SC, Cao ZH, Li ZG, Cheung KC, Wong MH. Effects of biofertilizer containing N-fixer, P and K solubilizers and AM fungi on maize growth: a greenhouse trial. Geoderma. 2005;125: 155–166. doi:10.1016/j.geoderma.2004.07.003
7. Elhaissoufi W, Ghoulam C, Barakat A, Zeroual Y, Bargaz A. Phosphate bacterial solubilization: A key rhizosphere driving force enabling higher P use efficiency and crop productivity. Journal of Advanced Research. 2022;38: 13–28. doi:10.1016/j.jare.2021.08.014
8. El-Tohamy W, El-Abagy H, El-Greadly N, Gruda N. Hormonal changes, growth and yield of tomato plants in response to chemical and bio-fertilization application in sandy soils. Journal of Applied Botany and Food Quality. 2008;82.
9. Zhang H, Zhu J, Gong Z, Zhu J-K. Abiotic stress responses in plants. Nat Rev Genet. 2022;23: 104–119. doi:10.1038/s41576-021-00413-0
10. Baum C. Interactive effects of plant growth-promoting rhizobacteria and organic fertilization on P nutrition of Zea mays L. and Brassica napus L. Journal of Plant Nutrition and Soil Science. 2011 [cited 12 Jul 2024]. Available: https://www.academia.edu/20876205/Interactive_effects_of_plant_growth_promoting_rhizobacteria_and_organic_fertilization_on_P_nutrition_of_Zea_mays_L_and_Brassica_napus_L
11. Jha Y, Mohamed HI. Inoculation with Lysinibacillus fusiformis Strain YJ4 and Lysinibacillus sphaericus Strain YJ5 Alleviates the Effects of Cold Stress in Maize Plants. Gesunde Pflanzen. 2023;75: 77–95. doi:10.1007/s10343-022-00666-7
12. Kumari R, Pandey E, Bushra S, Faizan S, Pandey S. Plant Growth Promoting Rhizobacteria (PGPR) induced protection: A plant immunity perspective. Physiol Plant. 2024;176: e14495. doi:10.1111/ppl.14495
13. Rizvi A, Chandrawal R, Khan MohdH, Ahmed B, Umar S, Khan MohdS. Microbiological Control of Xanthomonas Induced Bacterial Leaf Streak Disease of Wheat via Phytocompounds and ROS Processing Enzymes Produced Under Biotic Stress. J Plant Growth Regul. 2024;43: 601–623. doi:10.1007/s00344-023-11119-4
14. Ahmad M, Imtiaz M, Nawaz MS, Mubeen F, Sarwar Y, Hayat M, et al. Thermotolerant PGPR consortium B3P

modulates physio-biochemical and molecular machinery for enhanced heat tolerance in maize during early vegetative growth. Annals of Microbiology. 2023;73: 34. doi:10.1186/s13213-023-01736-5
15. Guo J, Bowatte S, Hou F. Diversity of endophytic bacteria and fungi in seeds of Elymus nutans growing in four locations of Qinghai Tibet Plateau, China. Plant Soil. 2021;459: 49–63. doi:10.1007/s11104-020-04608-y
16. Long J, Liu D, Qiao W, Wang Y, Miao Y, Baosai H. Response of Elymus nutans Griseb. seedling physiology and endogenous hormones to drought and salt stress. Sci Rep. 2024;14: 17810. doi:10.1038/s41598-024-68894-7
17. Quan X, Qiao Y, Chen M, Duan Z, Shi H. Comprehensive evaluation of the allelopathic potential of Elymus nutans. Ecology and Evolution. 2021;11: 12389–12400. doi:10.1002/ece3.7982
18. Li M, Yang J, Wang X, Li D, Zhang C, Tian Z, et al. Transcriptome profiles identify the common responsive genes to drought stress in two *Elymus* species. Journal of Plant Physiology. 2020;250: 153183. doi:10.1016/j.jplph.2020.153183
19. Agarwal P, Singh PC, Chaudhry V, Shirke PA, Chakrabarty D, Farooqui A, et al. PGPR-induced OsASR6 improves plant growth and yield by altering root auxin sensitivity and the xylem structure in transgenic Arabidopsis thaliana. J Plant Physiol. 2019;240: 153010. doi:10.1016/j.jplph.2019.153010
20. Gul F, Khan IU, Rutherford S, Dai Z-C, Li G, Du D-L. Plant growth promoting rhizobacteria and biochar production from Parthenium hysterophorus enhance seed germination and productivity in barley under drought stress. Front Plant Sci. 2023;14. doi:10.3389/fpls.2023.1175097
21. Landa BB, Montes-Borrego M, Navas-Cortés JA. Use of PGPR for Controlling Soilborne Fungal Pathogens: Assessing the Factors Influencing Its Efficacy. In: Maheshwari DK, editor. Bacteria in Agrobiology: Disease Management. Berlin, Heidelberg: Springer; 2013. pp. 259–292. doi:10.1007/978-3-642-33639-3_10
22. Singh R, Jha P. The PGPR Stenotrophomonas maltophilia SBP-9 Augments Resistance against Biotic and Abiotic Stress in Wheat Plants. Frontiers in Microbiology. 2017;8: 1945. doi:10.3389/fmicb.2017.01945
23. Zhang X, Yang Z, Wang L, Yue Y, Wang L, Xiulian Yang. The effects of plant growth-promoting rhizobacteria on plants under temperature stress:A meta-analysis. Rhizosphere. 2023;28: 100788. doi:10.1016/j.rhisph.2023.100788
24. Hardy RWF, Holsten RD, Jackson EK, Burns RC. The Acetylene-Ethylene Assay for N_2 Fixation: Laboratory and Field Evaluation. Plant Physiology. 1968;43: 1185–1207.
25. Murphy J, Riley JP. A modified single solution method for the determination of phosphate in natural waters. Analytica Chimica Acta. 1962;27: 31–36. doi:10.1016/S0003-2670(00)88444-5
26. Nomura H, Komori T, Uemura S, Kanda Y, Shimotani K, Nakai K, et al. Chloroplast-mediated activation of plant immune signalling in Arabidopsis. Nat Commun. 2012;3: 926. doi:10.1038/ncomms1926
27. Wang M, Tachibana S, Murai Y, Li L, Lau SYL, Cao M, et al. Indole-3-Acetic Acid Produced by Burkholderia heleia Acts as a Phenylacetic Acid Antagonist to Disrupt Tropolone Biosynthesis in Burkholderia plantarii. Sci Rep. 2016;6: 22596. doi:10.1038/srep22596
28. Weselowski B, Nathoo N, Eastman AW, MacDonald J, Yuan Z-C. Isolation, identification and characterization of Paenibacillus polymyxa CR1 with potentials for biopesticide, biofertilization, biomass degradation and biofuel production. BMC Microbiol. 2016;16: 244. doi:10.1186/s12866-016-0860-y
29. Galkiewicz JP, Kellogg CA. Cross-kingdom amplification using bacteria-specific primers: complications for studies of coral microbial ecology. Appl Environ Microbiol. 2008;74: 7828–7831. doi:10.1128/AEM.01303-08
30. Johnson JS, Spakowicz DJ, Hong B-Y, Petersen LM, Demkowicz P, Chen L, et al. Evaluation of 16S rRNA gene sequencing for species and strain-level microbiome analysis. Nat Commun. 2019;10: 5029. doi:10.1038/s41467-019-13036-1
31. Wang M, Tachibana S, Murai Y, Li L, Lau SYL, Cao M, et al. Correction: Corrigendum: Indole-3-Acetic Acid Produced by Burkholderia heleia Acts as a Phenylacetic Acid Antagonist to Disrupt Tropolone Biosynthesis in Burkholderia plantarii. Sci Rep. 2016;6: 26217. doi:10.1038/srep26217
32. Vijay S, Nair RR, Sharan D, Jakkala K, Ajitkumar P. Percoll discontinuous density gradient centrifugation method for the fractionation of the subpopulations of *Mycobacterium smegmatis* and *Mycobacterium tuberculosis* from *in vitro* cultures. MethodsX. 2023;11: 102344. doi:10.1016/j.mex.2023.102344
33. Zhao X, Zhong Y, Zhou W. Molecular mechanisms of mesocotyl elongation induced by brassinosteroid in maize under deep-seeding stress by RNA-sequencing, microstructure observation, and physiological metabolism. Genomics. 2021;113: 3565–3581. doi:10.1016/j.ygeno.2021.08.020
34. Xu F, Chu C, Xu Z. Effects of different fertilizer formulas on the growth of loquat rootstocks and stem lignification. Sci Rep. 2020;10: 1033. doi:10.1038/s41598-019-57270-5
35. Gurrieri L, Merico M, Trost P, Forlani G, Sparla F. Impact of Drought on Soluble Sugars and Free Proline Content in Selected Arabidopsis Mutants. Biology. 2020;9. doi:10.3390/biology9110367

36. Kielkopf CL, Bauer W, Urbatsch IL. Bradford Assay for Determining Protein Concentration. Cold Spring Harb Protoc. 2020;2020: 102269. doi:10.1101/pdb.prot102269
37. Isfan D. Nitrogen physiological efficiency index in some selected spring barley cultivars. Journal of Plant Nutrition. 1990;13: 907–914. doi:10.1080/01904169009364125
38. Novoa R, Loomis RS. Nitrogen and plant production. Plant and Soil. 1981;58: 177–204. doi:10.1007/BF02180053
39. Fageria NK, Baligar VC. Enhancing Nitrogen Use Efficiency in Crop Plants. Advances in Agronomy. Academic Press; 2005. pp. 97–185. doi:10.1016/S0065-2113(05)88004-6
40. Olasupo IO, Liang Q, Zhang C, Islam MS, Li Y, Yu X, et al. Agronomic Biofortification of Cayenne Pepper Cultivars with Plant Growth-Promoting Rhizobacteria and Chili Residue in a Chinese Solar Greenhouse. Microorganisms. 2021;9: 2398. doi:10.3390/microorganisms9112398
41. Aw X, Z L, Wc L, Zh Y. The effect of plant growth-promoting rhizobacteria (PGPR) on arsenic accumulation and the growth of rice plants (Oryza sativa L.). Chemosphere. 2020;242: 125136. doi:10.1016/j.chemosphere.2019.125136
42. Dhar Purkayastha G, Mangar P, Saha A, Saha D. Evaluation of the biocontrol efficacy of a Serratia marcescens strain indigenous to tea rhizosphere for the management of root rot disease in tea. PLoS One. 2018;13: e0191761. doi:10.1371/journal.pone.0191761
43. Ansari FA, Ahmad I. Fluorescent Pseudomonas -FAP2 and Bacillus licheniformis interact positively in biofilm mode enhancing plant growth and photosynthetic attributes. Sci Rep. 2019;9: 4547. doi:10.1038/s41598-019-40864-4
44. del Rosario Cappellari L, Santoro MV, Reinoso H, Travaglia C, Giordano W, Banchio E. Anatomical, morphological, and phytochemical effects of inoculation with plant growth- promoting rhizobacteria on peppermint (Mentha piperita). J Chem Ecol. 2015;41: 149–158. doi:10.1007/s10886-015-0549-y
45. Khoso MA, Wagan S, Alam I, Hussain A, Ali Q, Saha S, et al. Impact of plant growth-promoting rhizobacteria (PGPR) on plant nutrition and root characteristics: Current perspective. Plant Stress. 2024;11: 100341. doi:10.1016/j.stress.2023.100341
46. Guan H, Liu X, Fu Y, Han X, Wang Y, Li Q, et al. The locoweed endophyte Alternaria oxytropis affects root development in Arabidopsis in vitro through auxin signaling and polar transport. J Exp Bot. 2023;74: 931–944. doi:10.1093/jxb/erac430
47. Fan H, Wu J, Liu W, Yuan Y, Hu L, Cai Q. Linkages of plant and soil C:N:P stoichiometry and their relationships to forest growth in subtropical plantations. Plant Soil. 2015;392: 127–138. doi:10.1007/s11104-015-2444-2
48. Mantelin S, Touraine B. Plant growth-promoting bacteria and nitrate availability: impacts on root development and nitrate uptake. Journal of Experimental Botany. 2004;55: 27–34. doi:10.1093/jxb/erh010
49. Sun W, Shahrajabian MH. Biostimulant and Beyond: Bacillus spp., the Important Plant Growth-Promoting Rhizobacteria (PGPR)-Based Biostimulant for Sustainable Agriculture. Earth Syst Environ. 2025 [cited 27 Jan 2025]. doi:10.1007/s41748-024-00552-4
50. Ruiz Herrera LF, Shane MW, López-Bucio J. Nutritional regulation of root development. Wiley Interdiscip Rev Dev Biol. 2015;4: 431–443. doi:10.1002/wdev.183
51. Li X, Zeng R, Liao H. Improving crop nutrient efficiency through root architecture modifications. Journal of Integrative Plant Biology. 2016;58: 193–202. doi:10.1111/jipb.12434
52. Das PP, Singh KR, Nagpure G, Mansoori A, Singh RP, Ghazi IA, et al. Plant-soil-microbes: A tripartite interaction for nutrient acquisition and better plant growth for sustainable agricultural practices. Environ Res. 2022;214: 113821. doi:10.1016/j.envres.2022.113821
53. Egamberdiyeva D. The effect of plant growth promoting bacteria on growth and nutrient uptake of maize in two different soils. Applied Soil Ecology. 2007;36: 184–189. doi:10.1016/j.apsoil.2007.02.005
54. Krey T, Caus M, Baum C, Ruppel S, Eichler-Löbermann B. Interactive effects of plant growth–promoting rhizobacteria and organic fertilization on P nutrition of Zea mays L. and Brassica napus L. Journal of Plant Nutrition and Soil Science. 2011;174: 602–613. doi:10.1002/jpln.200900349
55. Wei T, Wang Y, Xie Z, Guo D, Chen C, Fan Q, et al. Enhanced ROS scavenging and sugar accumulation contribute to drought tolerance of naturally occurring autotetraploids in Poncirus trifoliata. Plant Biotechnol J. 2019;17: 1394–1407. doi:10.1111/pbi.13064
56. Selvarajan D, Mohan C, Dhandapani V, Nerkar G, Jayanarayanan AN, Vadakkancherry Mohanan M, et al. Differential gene expression profiling through transcriptome approach of Saccharum spontaneum L. under low temperature stress reveals genes potentially involved in cold acclimation. 3 Biotech. 2018;8: 195. doi:10.1007/s13205-018-1194-2
57. Janská A, Marsík P, Zelenková S, Ovesná J. Cold stress and acclimation - what is important for metabolic adjustment?

Plant Biol (Stuttg). 2010;12: 395–405. doi:10.1111/j.1438-8677.2009.00299.x
58. Batool T, Ali S, Seleiman MF, Naveed NH, Ali A, Ahmed K, et al. Plant growth promoting rhizobacteria alleviates drought stress in potato in response to suppressive oxidative stress and antioxidant enzymes activities. Sci Rep. 2020;10: 16975. doi:10.1038/s41598-020-73489-z
59. Bruno B, Karthik C, Ma Y, Kadirvelu K, Freitas H, Rajkumar M. Amelioration of chromium and heat stresses in Sorghum bicolor by Cr6+ reducing-thermotolerant plant growth promoting bacteria. Chemosphere. 2020;244: 125521. doi:10.1016/j.chemosphere.2019.125521
60. Patani A, Prajapati D, Ali D, Kalasariya H, Yadav VK, Tank J, et al. Evaluation of the growth-inducing efficacy of various Bacillus species on the salt-stressed tomato (Lycopersicon esculentum Mill.). Front Plant Sci. 2023;14. doi:10.3389/fpls.2023.1168155
61. Zhuoma P, Tondrob D, Qunpei T, Fu J, Dan S. Muti-omics revealed the mechanisms of MT-conferred tolerance of Elymus nutans Griseb. to low temperature at XiZang. BMC Plant Biology. 2024;24: 901. doi:10.1186/s12870-024-05583-7
62. Long J, Gao X, Miao Y. Effects of environmental factors on the phenotypic traits and seed element accumulation of wild Elymus nutans in Tibet. Sci Rep. 2025;15: 1838. doi:10.1038/s41598-025-85415-2
63. Theocharis A, Clément C, Barka EA. Physiological and molecular changes in plants grown at low temperatures. Planta. 2012;235: 1091–1105. doi:10.1007/s00425-012-1641-y
64. Yamori N, Levine CP, Mattson NS, Yamori W. Optimum root zone temperature of photosynthesis and plant growth depends on air temperature in lettuce plants. Plant Mol Biol. 2022;110: 385–395. doi:10.1007/s11103-022-01249-w
65. Luo H, Xu H, Chu C, He F, Fang S. High Temperature can Change Root System Architecture and Intensify Root Interactions of Plant Seedlings. Front Plant Sci. 2020;11. doi:10.3389/fpls.2020.00160
66. Bruno LB, Karthik C, Ma Y, Kadirvelu K, Freitas H, Rajkumar M. Amelioration of chromium and heat stresses in *Sorghum bicolor* by Cr6+ reducing-thermotolerant plant growth promoting bacteria. Chemosphere. 2020;244: 125521. doi:10.1016/j.chemosphere.2019.125521
67. Zhao X, Yuan X, Xing Y, Dao J, Zhao D, Li Y, et al. A meta-analysis on morphological, physiological and biochemical responses of plants with PGPR inoculation under drought stress. Plant, Cell & Environment. 2023;46: 199–214. doi:10.1111/pce.14466
68. Franco-Franklin V, Moreno-Riascos S, Ghneim-Herrera T. Are Endophytic Bacteria an Option for Increasing Heavy Metal Tolerance of Plants? A Meta-Analysis of the Effect Size. Front Environ Sci. 2021;8. doi:10.3389/fenvs.2020.603668

Table 1 Growth promoting characteristics of four PGPR strains

Strains	Nitrogen fixation capacity ($\mu g\ mL^{-1}$)	Inorganic phosphorus solubilization capacity ($\mu g\ mL^{-1}$)	Organic phosphorus solubilization capacity ($\mu g\ mL^{-1}$)	Potassium-solubilising capacity ($\mu g\ mL^{-1}$)	IAA contents ($\mu g\ mL^{-1}$)
S1	5.10±0.07	176.2±62.46	24.73±15.91	4.42±0.34	596.87±63.7
S2	-	136.32±68.19	79.85±5.34	11.42±0.59	197.10±22.80
S3	2.08±0.06	250.24±30.11	44.52±26.51	8.18±0.42	193.32±7.17
S4	5.06±0.02	172.09±31.22	47.34±4.24	6.05±0.35	168.77±21.35
S5	5.57±0.03	311.95±50.06	49.46±5.61	4.59±0.44	166.64±16.88

Table 2 The main effects of temperature (T), PGPR, and fertilizers (NPK) on *Elymus nutans Griseb*. The values in the table represent the statistical significance (*p* - values) of the effects of different sources of variation on each trait. PT: plant height; DW: dry weight, Chl: plant chlorophyll; RL: root length; RB: number of root branches; RSA: root surface area; RD: root average diameter; SS: soluble sugars; SP: soluble proteins.

**Source of variation**	**PT**	**DW**	**Chl**	**RL**	**RB**	**RSA**	**RD**	**SS**	**SP**
**T**	<0.001	<0.001	<0.001	<0.001	0.076	<0.001	<0.001	<0.001	<0.001
**NPK**	<0.001	<0.001	<0.001	<0.001	<0.001	<0.001	0.847	<0.001	<0.001
**PGPR**	<0.001	<0.001	<0.001	<0.001	<0.001	0.007	<0.001	<0.001	<0.001
**T* NPK**	0.333	0.007	<0.001	0.429	0.893	0.012	0.015	<0.001	<0.001
**T* PGPR**	0.629	0.028	<0.001	<0.001	<0.001	0.392	<0.001	<0.001	<0.001
**NPK* PGPR**	0.386	0.007	<0.001	0.174	0.949	0.728	<0.001	<0.001	<0.001
**T* NPK* PGPR**	0.290	0.026	<0.001	0.020	0.898	0.010	0.181	<0.001	<0.001

Note：When $p < 0.05$, it shows significant impact. When $p < 0.01$, it signifies an extremely remarkable influence.

Fig.1. General location and climatic parameters of three sampling sites. Three randomly selected areas where *Elymus nutans Griseb* was growing vigorously. The map, base map, shape files, and map data were obtained from the National Geographic Information Public Service Platform "TianDiTu" (https://cloudcenter.tianditu.gov.cn/dataSource), the specific URL of the web page for retrieving this data is: https: //cloudcenter.tianditu.gov.cn/administrativeDivision/ . The map base layer has been reviewed and approved with the approval number GS (2024) 0650. The base map is unmodified.

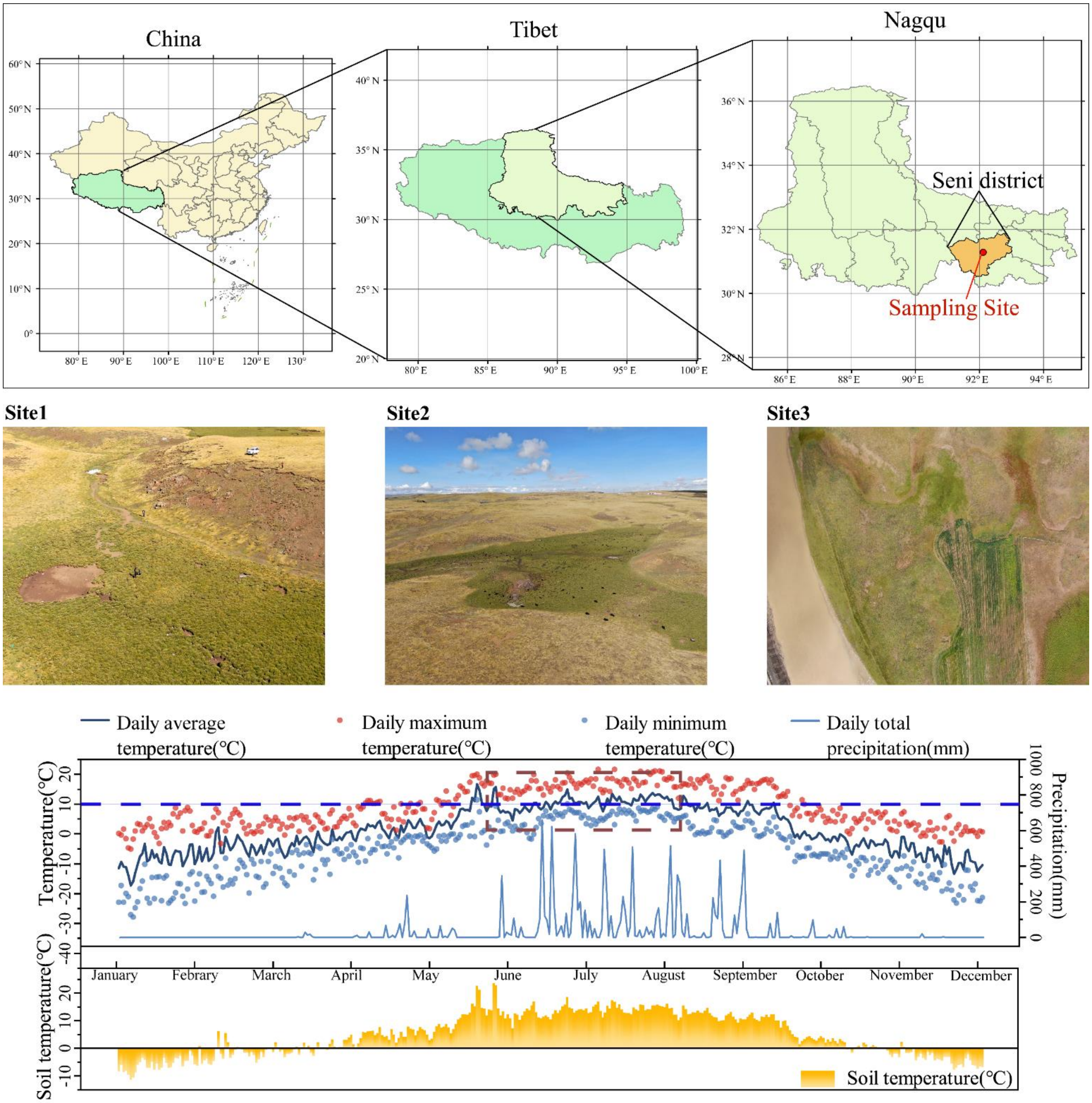

Fig.2. The effects of different treatments on the growth of *En*G at different temperatures. T1(addition of PGPR and no addition of NPK fertilizers), T2 (neither PGPR nor NPK fertilizers), T3 (addition of PGPR and NPK fertilizers), and T4 (no addition of PGPR, but addition of NPK fertilizers).

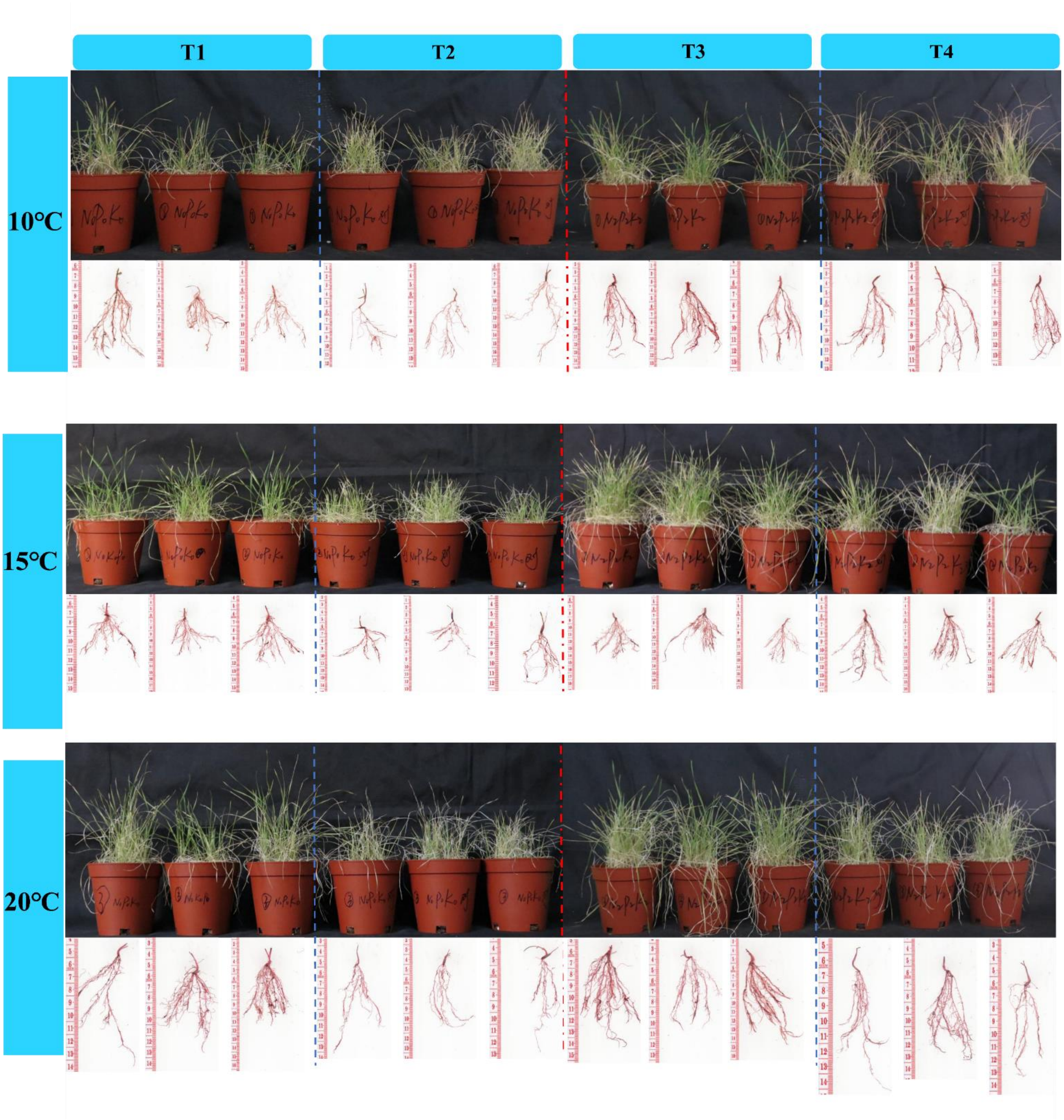

Fig.3. Effect of different treatments on plant height (PT), dry weight (DW), and plant chlorophyll (Chl)content. Lowercase letters indicate differences between treatments and uppercase letters indicate differences between temperatures.

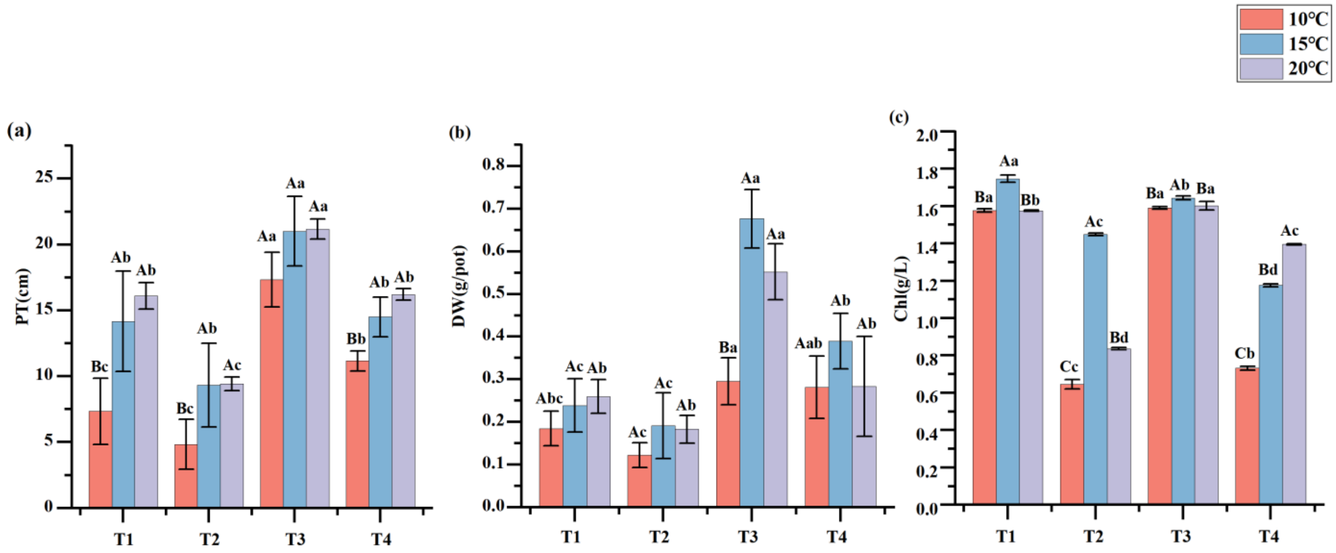

Fig.4. Effect of different treatments on root length (RL), number of root branches (RB), root surface area (RSA), and root average diameter (RD). Lowercase letters indicate differences between treatments and uppercase letters indicate differences between temperatures.

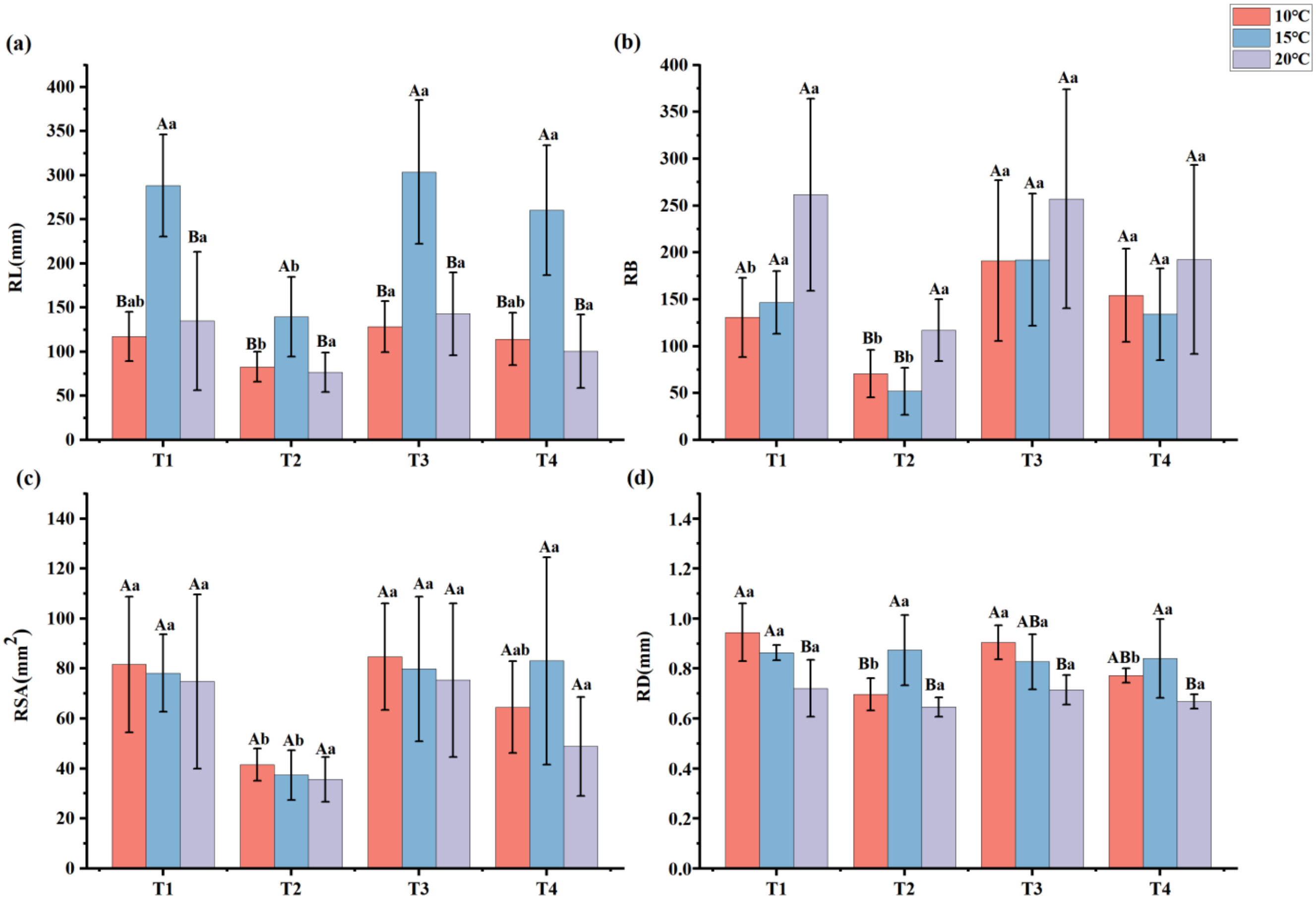

Fig.5. Effect of different treatments on soluble sugars (SS), and soluble proteins (SP)content. Lowercase letters indicate differences between treatments and uppercase letters indicate differences between temperatures.

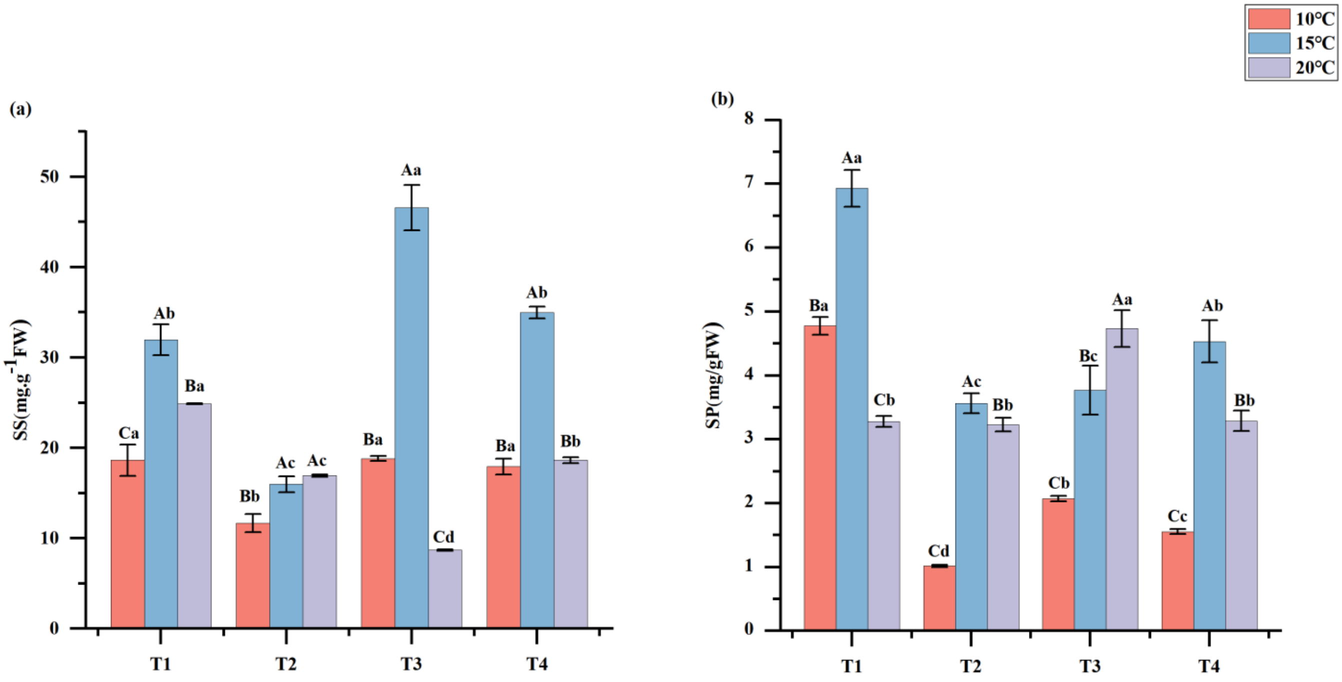

Fig.6. Effect of different treatments (T3 and T4) on physiological efficiency of nitrogen (NPE), phosphorus (PPE), potassium (KPE), agronomic efficiency of nitrogen (NAE), phosphorus (PAE) and potassium (KAE), and apparent recovery efficiency of nitrogen (NRE), phosphorus (PRE) and potassium (KRE) content. * means $p < 0.05$, ** means $p < 0.01$, *** means $p < 0.001$, ns means no significant difference.

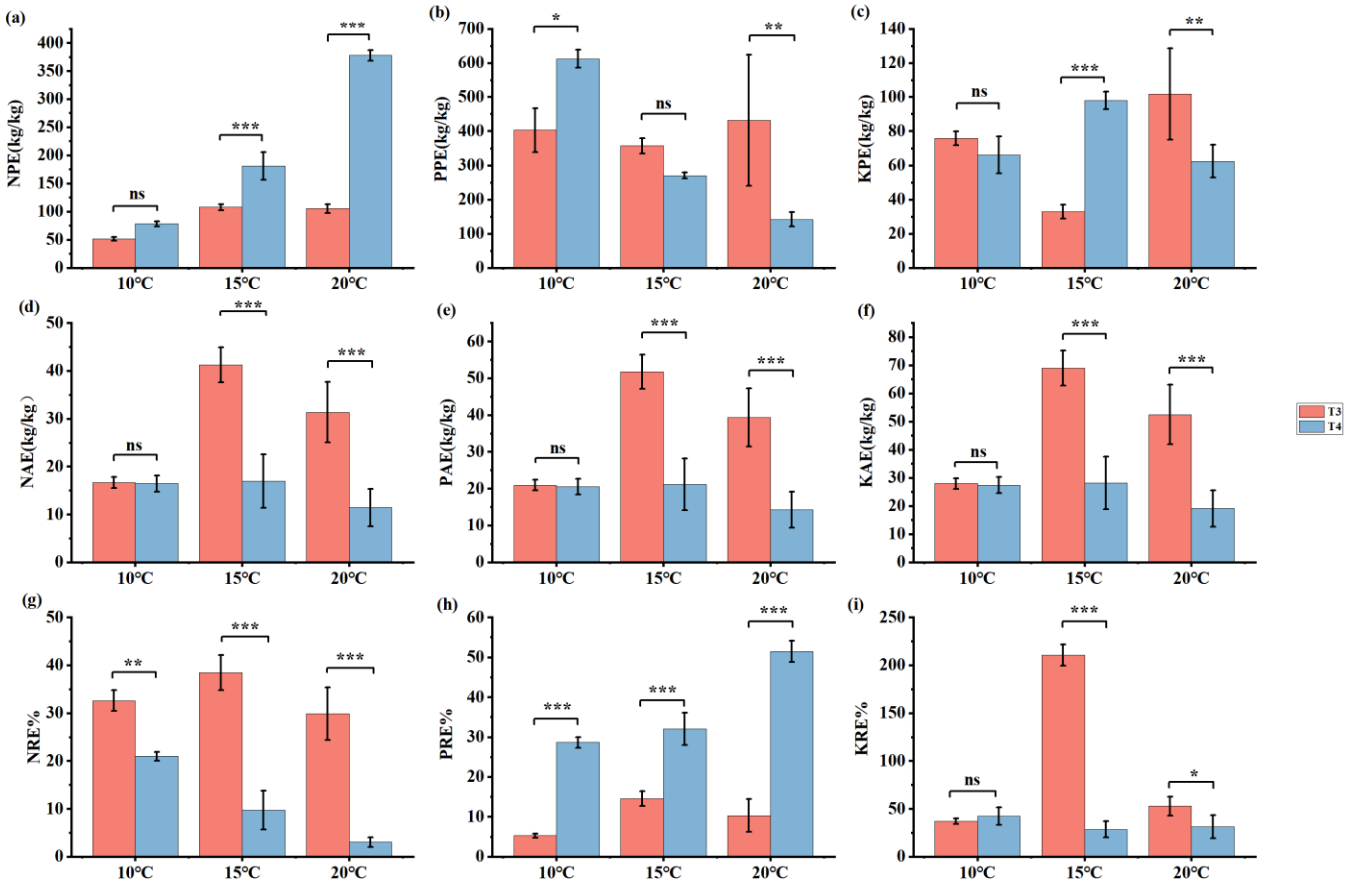

Fig.7. (a) Spearman's correlation between the relative abundance of PE, AE, and RE, and Plant growth factors. *, **, and *** represent significant correlations at the 0.05, 0.01, and 0.001 levels. (b) Mantel test between nutrient utilization and indicators of plant growth. PT, plant height; DW, dry weight; Chl, plant chlorophyll; RL, root length; RB, number of root branches; RSA, root surface area; RD, root average diameter; SS, soluble sugars; SP, soluble proteins.

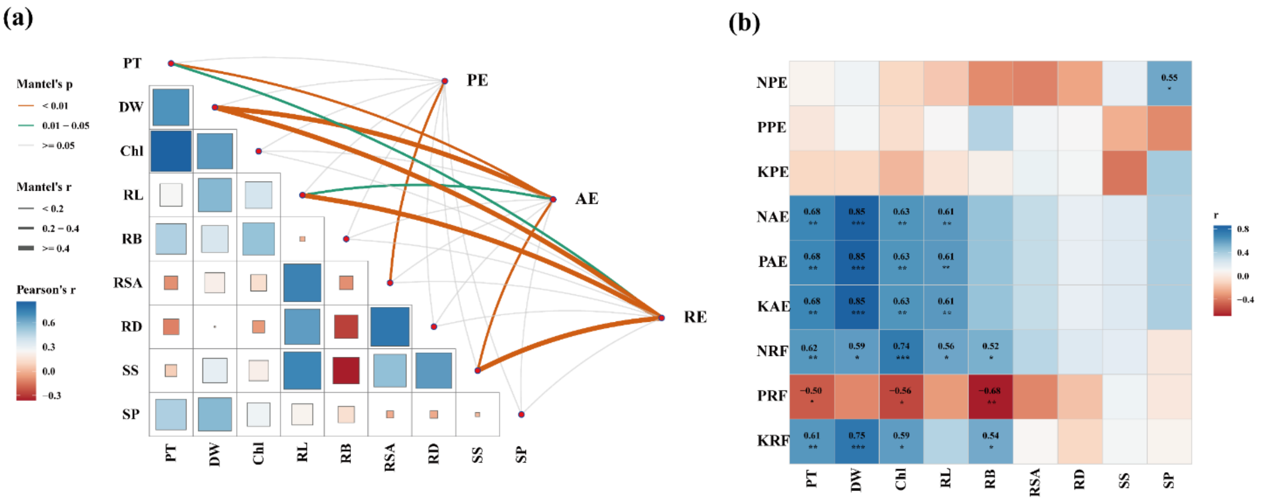

Click here to access/download
Supporting Information - Compressed/ZIP File Archive
Base Map Data Source.zip

Effect of PGPR on growth and nutrient utilization of *Elymus nutans* Griseb at different temperatures

Linling Ran [a,1], Haoyang Wu [a], Fei Xia [b,c,1], Yunyin Xue [d], Wei Wei [b,c], Junqiang Wang [a,*], Jinglong Wang[b,c], Shanshan Zhao [a], Shuang Yan [a], Hao Shi[e], Shaikun Zheng[e], Yu Zhang [a], Xiaoqin Qiu [a]

[a] Key Laboratory of Southwest China Wildlife Resource Conservation (Ministry of Education). China West Normal University, Nanchong, 637009, China

[b] State Key Laboratory of Highland Barley and Yak Germplasm Resources and Genetic Improvement, Lhasa, 850000, China

[c] Institute of Pratacultural Science, Tibet Academy of Agriculture and Animal Husbandry Science, Lhasa, 850000, China

[d] School of Geography and Tourism, Shaanxi Normal University, Xi'an, 710119, China,

[e] College of Forestry, Gansu Agricultural University, Lanzhou, 730070, China

[*] Corresponding author at: Key Laboratory of Southwest China Wildlife Resource Conservation (Ministry of Education). China West Normal University, Nanchong,637009, China.

[1] These authors contributed equally to this work and should be considered co-first authors.

Tel: 0086/457/17390270491.

E-mail address: wangjunq0303@163.com (J.-Q. Wang).

Abstract

In order to show the growth-promoting effects of PGPR (S1, S2, S3, S4 and S5) isolated from the rhizosphere soil of *Elymus nutans* Griseb (*En*G) on the Tibetan Plateau. In this study, we simulated three temperature gradients (10°C, 15°C, and 20°C) during the growing season of *Elymus nutans* Griseb on the Tibetan Plateau, and set up four incubation substrate treatments:T1(addition of PGPR and no addition of NPK fertilizers), T2 (neither PGPR nor NPK fertilizers), T3 (addition of PGPR and NPK fertilizers), and T4 (no addition of PGPR, but addition of NPK fertilizers), to explore the effects of PGPR on the growth and nutrient (NPK) utilization of *Elymus nutans* Griseb at different temperatures. The results showed that compared with T2, the plant height (PT) of T1 increased by 51.72% to 70.67%, the dry weight (DW) increased by 24.99% to 51.25%, the soluble sugar (SS) and soluble protein (SP) content significantly increased by 59.37% and 369.66% at 10 °C respectively (p<0.05), and significantly increased by 100.17% and 94.5% at 15 °C respectively (p<0.05). Compared with T4, T3 significantly decreased the physiological efficiency of N (NPE) at 15 °C and 20 °C by 40.43% and 72.11%, respectively. In summary, our results showed that this PGPR can promote the growth of *Elymus nutans* Griseb and improve its nutrient utilization efficiency.

Keywords: Plant Growth-Promoting Rhizobacteria (PGPR); Nutrient Uptake; Temperature Effects; *Elymus nutans* Griseb; Resistance

1. Introduction

Under natural conditions, plants perpetually engage in intricate relationships with a microbial community known as the phytomicrobiome [1]. Certain soil microorganisms contribute to the enhancement of plant growth and progression [2]. Plant growth-promoting rhizobacteria (PGPR), a type

of beneficial bacteria found in nature, inhabit plant roots and enhance plant development [3]. PGPR encompasses numerous soil bacteria species that promote the growth of host plants when cultivated in their presence [4]. These microbes populate the root zone of plants and boost plant growth either directly or indirectly [5]. Wu [6] showed that the application of *Bacillus megaterium* and *Bacillus mucilaginous* as microbial inoculants enhanced plant growth and enhanced the plant's uptake of nutrients (total N, P, and K). These microbes play essential roles in the solubilization of P and K and nitrogen fixation. They transform insoluble P and K in the soil into plant-accessible forms via acidolysis, chelation, and exchange reactions, which in turn stimulate plant growth [7]. In addition, PGPR can regulate phytohormone levels in plants and thereby influence plant growth [8].

Plants face diverse environmental challenges that substantially hamper their growth and progression, potentially causing significant crop losses in farming. Cold temperatures represent a key non-biological element that restricts the growth and output of plants [9]. There is extensive documentation that suitable management techniques, encompassing the use of fertilizers and external protective compounds, can reduce the harmful influence of cold stress on plant growth [10]. PGPR can enhance plant nutrient uptake and accessibility via biochemical and physiological processes [11], and also enhance plant stress tolerance through systemic resistance induction, producing phytohormones, solubilizing nutrients [12,13]. Nevertheless, there are significant differences in temperature tolerance among different PGPR strains, and these differences directly affect their efficacy in plant growth [14]. Therefore, it is crucial to choose PGPR strains that are suitable for a particular forage and its environment.

Elymus nutans Griseb (*En*G) serves as a native and widespread grazing grass commonly found in the highland pastures of the Qinghai-Tibetan Plateau in China [15]. As an excellent pasture grass in the Qinghai-Tibetan Plateau region in China, it has strong resistance and tolerance to cold, rich nutrients, good palatability, high yield, and tolerance to desert areas among others. It can also prevent winds and fix sands,

conserve soil and water, and accelerate the process of ecological restoration of the grassland, which can effectively reduce the economic losses caused by natural disasters [16]. Thus, it is widely planted in the restoration of degraded alpine meadows on the Qinghai - Tibet Plateau [17].

The growth of *En*G relies on favorable relationships between its roots and microbes, which are essential for nutrient uptake and enhancing growth in its habitat [18]. Therefore, investigating the PGPR inhabiting the rhizosphere of *En*G on the Qinghai-Tibetan Plateau is crucial. Several studies demonstrate that PGPR can fix N, solubilize P, detoxify K, and secrete indole-3-acetic acid (IAA) to promote growth [19–22]. Nonetheless, few studies examined the effects of temperature on PGPR-mediated effects or PGPR inoculum colonization [23]. To systematically elucidate the growth-promoting mechanism of PGPR at different temperatures and to enhance the yield of *En*G, we aimed to: (1) investigate the effects of PGPR on the growth and nutrient utilization of *En*G and (2) on *En*G at different temperatures. The findings of this study help identify valuable microbial resources and provide foundational insights, thus supporting the development and application of PGPR as a biofertilizer to enhance pasture productivity on the Tibetan Plateau.

2. Materials and methods

2.1. Sources of PGPR

The sampling area is located in Nagqhu Town, Seni District, Nagqhu City, Tibet Autonomous Region, with geographic coordinates of 31°26′N latitude and 92°07′E longitude (Fig. 1), and an average elevation of 4450 m. Three sampling sites with good growth and disease-free *En*G were randomly selected, and the rhizosphere soil of the *En*G was collected using root shaking method, with five replicate mixes randomly sampled at each site. And the rhizosphere soil, together with the fine roots, were put into sterile self-sealing bags and stored at low temperature (4°C) to be brought back to the laboratory. The collected soil samples

were prepared into 10^{-4} and 10^{-5} soil suspensions and 100 μL of soil suspension was aspirated and spread evenly on beef paste peptone agar plates for bacterial purification and isolation of bacteria.

2.2. Determination of the growth-promoting ability of PGPR and screening of high-quality bacterial strains

Inoculate the purified strains onto Nitrogen free medium (NFM), Pikovskaya medium, Bacterial Organophosphorus medium, Potassium Bacteria medium and King's B medium respectively to determine whether the strains have growth - promoting abilities. Among them, 273 strains can grow on the selective medium. The ability of the isolates to grow on NFM agar and their nitrogen-fixing capacity was evaluated using the acetylene reduction method [24]. Strains' phosphorus solubilizing capacity was tested with Mo blue colorimetry [25]. The strains were cultured in 50 mL King's broth at 28 °C with shaking (180 rpm) for 3 days. IAA was extracted from the culture filtrate using ethyl acetate, with each extraction done three times [26]. The IAA content was measured using high-performance liquid chromatography [27]. The KSB-containing solution was diluted to 50 mL and centrifuged at 500 rpm for 10 minutes to eliminate solids. Subsequently, a 10 mL aliquot was further centrifuged at 10,000 rpm for 10 minutes, and the K in the supernatant was quantified using flame spectrophotometry [28]. Five excellent strains (S1, S2, S3, S4 and S5) with high growth promoting ability were screened by the determination of growth-promoting ability. All five strains solubilized inorganic and organic phosphates, decomposed K, and secreted IAA. In addition, S1, S3, S4, and S5 fixed N (Table 1).

2.3. Identification of PGPR strains

Five strains (S1, S2, S3, S4, and S5) were inoculated in LB liquid medium for incubation at 37 °C under shaking (160 rpm) for 24 h. Bacterial genomic DNA was extracted from these strains using a commercial kit (Tiangen Biochemical Technology, Beijing, China). The 16S rRNA gene was polymerase chain reaction (PCR) amplified with the primer set 27 F (5′-AGAGTTTGATCCTGGCTCAG-3′) and

1492R (5′-GGTTACCTTGTTACGACTT-3′) as described by Galkiewicz and Kellogg (2008) [29]. PCR was executed in a 50 μL volume, comprising 1 μL of 5 μM forward primer, 1 μL of 5 μM reverse primer, 25 μL of 2×Taq PCR MasterMix, 2 μL of DNA template, and double-distilled water. The PCR thermal cycling parameters were set to an initial denaturation at 95 °C for 5 minutes, followed by 30 cycles of 95 °C for 30 seconds, 57 °C for 60 seconds, and 72 °C for 30 seconds, with a concluding extension phase at 72 °C for 10 minutes. The PCR results were validated through 1% agarose gel electrophoresis and subsequently subjected to sequencing via the dideoxy chain-termination approach at Sangon Biotech Co., Ltd. (Shanghai, China) [30]. The sequences of the 16S rRNA genes from the five bacterial strains have been submitted and archived in the GenBank repository, and corresponding accession numbers have been assigned: PQ351188, PQ351189, PQ351190, PQ351191, and PQ351192.

2.4 Mixed bacterial solution and incubation substrate preparation

The compatibility between excellent strains (S1-S5) was verified by the plate confrontation method [31]. The excellent strains (S1 - S5) were inoculated into sterilized LB liquid medium respectively, and activated and cultured in a constant temperature shaker at 150 r/min and 30 °C for 12 h. The OD_{600} value of each activated bacterial solution was adjusted to 0.6. And then transferred to the fresh LB medium, 150r/min, 30°C constant temperature shaker to expand the culture for 16h, after doubling the bacterial solution after centrifugation to remove the supernatant, to the bacterial body of the re-suspension of sterile water, fully mixed and made into a single bacterial agent. After sufficient mixing and make the single bacterial agent with OD_{600} value of 0.6 (the concentration of the single bacterial agent is about 1×10^8CFU/ml). Equal amount of mixing to prepare a composite bacterial agent [32].

The culture substrate was formulated with reference to the soil characteristics of the sampling sites, which was composed of a blend of peat soil, vermiculite, perlite, and coir in a ratio of 4:1:1:1 (v/v), with

50 g per bag, which was then sterilized at 121 °C for 30 minutes. Subsequently, 78.2 g of the culture medium was combined with 1% (volume/volume) PGPR bacterial mixture and transferred into a 660 mL plastic container (70 × 95 × 98 mm) [33]. A cup with an equal volume of sterile water was used as the control.

2.5 Incubation experiment

The four treatments set up in this study were T1(addition of PGPR and no addition of NPK fertilizers), T2 (neither PGPR nor NPK fertilizers), T3 (addition of PGPR and NPK fertilizers), and T4 (no addition of PGPR, but addition of NPK fertilizers) at three replicates. The NPK represents applied rates of 150 mg kg^{-1}, 120 mg kg^{-1}, and 90 mg kg^{-1} of urea (N,46.65%), superphosphate (P_2O_5,12%) and potassiumchloride (K_2O,60%), respectively. Furthermore, according to the climatic temperature during the growing season (June, July, and August) of *En*G on the Tibetan Plateau (Fig. 1), this study was set up with temperature gradients of 10 °C, 15 °C, and 20 °C. Incubation was carried out in three incubators with a light (16 h)/dark (8 h) cycle (light at 350 μEm^{-2} s^{-1}) and 60% humidity. Plant seeds (wild *En*G seeds collected in Nagqhu, Tibet) were sterilized on the surface with a 1:1 mixture of 30% hydrogen peroxide and 70% ethanol for 10 minutes and cleaned repeatedly with demineralized sterile water. Thirty-five *En*G seeds were planted in each cup. In order to better simulate the plant growth cycle in alpine meadows, plant growth indicators were measured after 120 days of plant growth (based on the local plant growth season).

2.6 Indicators measurement

2.6.1 Dry weight (DW), plant height (PT), and root system assay

A random sample of 15 plants per pot was taken to determine plant height (PT). After drying at 65 °C for 24 h, the dry weight (DW) was noted. Root length (RL), the count of root branches (RB), root surface area (RSA), and the average root diameter (RD) were all assessed using an LA2400 root scanner (Jeili

Electronics Trading Co., Ltd, Shanghai, China) [34].

2.6.2 Plant chlorophyll (Chl), soluble sugars (SS), and soluble proteins (SP) content assay

To measure the chlorophyll (Chl) content, 0.1 g of fresh leaf tissue from each seedling was mixed with a bit of quartz sand, ground in 80% acetone, and then centrifuged at 9000 × g and 4 °C for 10 minutes. The supernatant's absorbance was read at 440, 663, and 645 nm to assess Chl a, b, and carotenoid levels, respectively. The total Chl content was computed using Li [18]. The soluble sugar (SS) content was assessed following the method of [35]. Soluble protein (SP) content was determined using Bradford's method for staining with Caulmer Brilliant Blue [36].

2.6.3 Plant nutrient use efficiency

The physiological efficiency (PE) of N (NPE), P (PPE), and K (KPE), i.e., the kg plant yield N, P, and K uptake, was calculated according to [37]. The agronomic efficiencies (AE) of N (NAE), P (PAE), and K (KAE), i.e., the yield increase for each kg N, P, K applied, was calculated according to Novoa and Loomis [39]. The apparent recovery efficiency (RE) of N (NRE), P (PRE), and K (KRE), i.e., plant N, P, and K uptake (kg ha^{-1}) per kg of N, P, and K applied, was calculated according to Fageria and Baligar [40].

2.7 Statistical analyses

Statistical analyses were performed using SPSS software (version 27.0; SPSS Inc., Chicago, IL, USA). In the context of our research, prior to conducting parametric tests on each dataset, we initiated a preliminary assessment using the Shapiro-Wilk test to ascertain the conformity of the data to a normal distribution pattern. Mean differences were determined using the least significant difference method and the Tukey test at a significance level of 5%. Statistical significance is reported at $p < 0.05$. All visualizations were produced using R 4.4.1. The relationship between the growth indicators of *En*G and nutrient utilization was analyzed through Mantel analysis.

3. Result

*3.1 Effects of PGPR on En*G *growth characteristics at different temperatures*

Substrate with PGPR microbial inoculants influenced the growth characteristics of *En*G (Fig.2). Compared to T2, treatment T1 increased PT by 51.72% (10 ℃) to 70.67% (20 ℃), and increased DW, accounting for 24.99% (15 ℃) to 51.25% (10 ℃) (Fig.3). Compared to T4, treatment T3 significantly increased PT by 30.66% (20 ℃) to 55.22% (10 ℃), and significantly increased DW, accounting for 4.86 % (10 ℃) to 94.71% (20 ℃) ($p<0.05$) (Fig.3a and 3c). Treatments T1 and T3 significantly increased the Chl of *EnG* compared to T2 and T4 ($p<0.05$) (Fig.3c).

*3.2 Effects of PGPR on En*G *root growth at different temperatures*

The effects of PGPR on the roots of *En*G are shown in Fig.4. Compared to T2, the T1 significantly increased RSA of *En*G by 96.32% and 109.31% at 10 °C and 15 °C, respectively ($p<0.05$) (Fig.4). Compared with the T4 treatment, the T3 treatment increased the RL, RB, RSA, and RD of *En*G. Furthermore, the RL of *En*G was significantly higher at 15 °C than at 20 °C ($p<0.05$) (Fig.4a).

*3.3 Effects of PGPR on SS and SP of En*G *at different temperatures*

Compared to treatment T2, T1 significantly increased the SS and SP of *En*G by 59.37% and 369.66% at 10 °C, by 100.17% and 94.5% at 15 °C, respectively ($p<0.05$) (Fig.5). Furthermore, the content of SS at 10 °C under the T3 treatment was significantly higher than that of SS at 20 °C (Fig. 5a). The contents of SS and SP at 10 °C under T1 were significantly higher than at 20 °C ($p< 0.05$) (Fig.5b).

*3.4 Effects of PGPR on En*G *nutrients use efficiency indexes at different temperatures*

Compared with treatment T4, T3 significantly decreased NPE of *En*G at 15°C and 20°C by 40.43% and 72.11%, respectively ($p<0.001$) (Fig.6a). However, compared with T4, T3 significantly decreased the PRE of *En*G ($p<0.001$) (Fig.6h). Furthermore, the T3 treatment significantly increased the NAE, PAE,

KAE, NRE, and KRE of *En*G at 15°C and 20°C ($p<0.001$) (Fig.6).

3.5 Relationship between PGPR and growth indicators of EnG

The PGPR significantly affected PT, DW, Chl, RL, RSA, RD, SS, and SP (Table 2). The interaction between temperature and PGPR significantly affected the RL of *En*G (Table 2) ($p<0.001$). In addition, RL was significantly and positively correlated with AE (Fig.7b) ($p<0.05$). PT and DW were highly significantly correlated with NAE, PAE, and KAE (Fig. 7a) ($p<0.001$), and PT and DW were significantly correlated with the apparent NRE, PRE, and KRE (Fig.7a) ($p<0.05$). In addition, RL was significantly and positively correlated with AE (Fig.7) ($p<0.05$).

4. Discussion

*4.1 The effects of PGPR on En*G *growth*

The plant microbiome has been recognized as a key plant characteristic influencing crop yield. Olasupo et al. [41] applied various bacillus treatments to peppers, resulting in a 27%–36% boost in biomass. Similarly, Aw et al. [42] isolated three As-resistant PGPR strains to verify their roles in promoting rice growth. In the present study, compared to the T2 treatment, the T1 treatment increased the PT and DW of *En*G (Fig.3). These findings corroborated the efficacy of PGPR in augmenting the growth of *En*G. Furthermore, the T3 treatment significantly increased the PT and DW of *En*G compared with T4 ($p<0.05$) (Fig.3a and 3c). There was also a significant correlation between PT, DW, AE, and RE (Fig.7a) ($p<0.05$). This correlation underscores the role of the culture substrate with PGPR in facilitating *En*G nutrient uptake. Consequently, the application of PGPR can improve soil nutrient use effectively and reduce the amount of chemical fertilizer applied and the negative effects of excessive fertilization [42], thereby, *En*G biomass can be increased, and economic and ecological development can be achieved.

Plant growth requires the accumulation of photochemical products and is directly linked to the

photosynthetic rate [43]. del Rosario Cappellari [45] evaluated the growth characteristics and Chl levels in peppermint (*Mentha piperita*) seedlings exposed to PGPR strains, noting that the treated plants demonstrated improved values for these metrics compared to the untreated controls. Consistent with these findings, our T1 treatment significantly increased the Chl content of *En*G compared with T2 ($p<0.05$) (Fig.3c). This indicates that the culture substrate with PGPR had a certain promoting effect on the photosynthesis of *En*G.

Furthermore, we found that treatment T1 significantly increased the RSA of *En*G at 15 °C by 109.31% compared with treatment T2 ($p<0.05$) (Fig.4). Earlier research implied that the impact of PGPR on root growth is connected to the synthesis of IAA, a substance known to advance root expansion and enhancement, augment the area of the root surface, and stimulate root metabolic processes [19]. Consistent with these findings, the culture substrate containing PGPR elicited IAA production (Table 1). The expansion of the root system surface area augments the nutrient absorption area, which is beneficial for facilitating the uptake of nutrients and water [45]. It is worth noting that there was a significant correlation between RSA levels and PE (Fig.7) ($p<0.01$). This indicates that the PGPR is capable of enhancing the nutrient uptake efficiency of *En*G by promoting root growth [46].

*4.2 The effect of PGPR on the nutrient utilization of En*G

N, P, and K are fundamental chemical elements essential for plant development, contributing significantly to plant tissue formation and physiological processes [47]. The effect of PGPR on plant nutrition stems from its impact on nutrient absorption by plants [48]. Wu et al. [6] found that Bacillus megaterium and Bacillus mucilaginous inoculants not only boosted plant growth but also enhanced the plants' nutrient absorption capabilities, particularly in terms of total N, P, and K. In this study, the T3 treatment significantly increased the NAE, PAE, and KAE of *En*G at 15°C and 20°C. This indicates that the PGPR is capable of enhancing the nutrient uptake efficiency of *En*G, reducing the dependence on

chemical fertilizers, and thereby promoting sustainable agricultural development [49]. In addition, the root system of plants performs several functions, including nutrient acquisition, water uptake, stability, and symbiotic relationships with beneficial soil microorganisms, which collectively enhance nutrient absorption efficiency [50]. In the present study, the AE and RE were significantly correlated with RL (Fig. 7a). In addition, NAE, PAE, KAE, and NRE were significantly positively correlated with RL (Fig.7b).These findings affirm that efficient nutrient uptake also relies significantly on the root system's capacity to permeate the soil [51].

Compared with treatment T4, T3 significantly decreased the NPE of *En*G at 20 °C by 72.11% (p<0.001) (Fig. 6a). This may be due to the fact that PGPR transform unavailable nutrient forms for plants into available forms [52], which reduces the plants' need for fertilizer. PGPR plays a significant role in agriculture by facilitating the circulation of plant nutrients and reducing the reliance on chemical fertilizers [53]. Our findings offer microbial resources and foundational data for the development and application of PGPR as biofertilizers; however, compared with t T4, the T3 treatment significantly decreased the PRE of *En*G (p<0.001) (Fig.6h). This aligns with previous research indicating that the use of PGPR can enhance P mobilization and supply to crops in soils deficient in P [54]. Therefore, we propose that the culture substrate with PGPR promotes nutrient uptake by *En*G and can help reduce the use of chemical fertilizers in agriculture.

4.3 The effects of PGPR at different temperatures

Under low-temperature stress conditions, plants exhibit a heightened accumulation of SS such as sucrose, glucose, fructose, ribose, and trehalose [55]. These sugars are capable of neutralizing free radicals and indirectly stimulating protein synthesis, which in turn enhances cold resistance [56]. SP are also pivotal, serving as osmoregulators and supporting low-temperature tolerance in plants [57]. Previous studies show that the contents of SP increased up to 68% in plants treated with PGPR [58]. Additionally,

the SS content was significantly elevated in PGPR-treated plants in a previous study, and it was approximately double that of the untreated controls [55]. Our findings corroborate these observations as compared with treatment T2, T1 significantly increased the SS and SP content of *En*G by 59.37% and 369.66% at 10 °C (Fig. 5). This suggests that the physiological and biochemical status of *En*G significantly improved under the influence of PGPR [59]. Thus, inoculating plants with PGPR can be a productive method to foster growth by enhancing their tolerance to various abiotic stresses [60]. It is worth noting that the content of SS and SP under the T1 and T3 treatment was significantly higher at 10 °C than at 20 °C ($p < 0.05$) (Fig. 5b). This is because low temperatures may induce changes in the activity of enzymes related to the synthesis of soluble sugars (SS). At 10 °C, these enzymes may be more inclined to break down and convert stored complex carbohydrates, such as starch, into soluble sugars. This is due to the fact that upon sensing the low - temperature environment, plants initiate a series of physiological adaptation mechanisms. They transform less mobile nutrient forms, like starch, into soluble sugars that are more easily transported and utilized, so as to maintain the osmotic balance and energy supply of cells [61,62].

Temperature plays a key role in restricting the distribution and output of plants [63]. It is a critical environmental factor that profoundly influences plant root growth, shaping root system architecture and influencing nutrient uptake and overall plant health [64]. In this study, the interaction between temperature and the application of PGPR inoculum significantly affected the root length of *En*G ($p<0.001$) (Table 2). This can be attributed to the combined effects of temperature on root development and the ability of PGPR to modulate plant hormone levels and improve nutrient and water uptake [65]. Consequently, the RL of *En*G was significantly higher at 15 °C than at 20 °C ($p<0.05$) (Fig.4a). However, under the influence of PGPR, the *En*G was more productive after inoculation with PGPR under temperature stress than under normal temperatures, in line with the results of L. B. Bruno [67]. An augmented plant biomass, stemming from nutrient accumulation, offers the most compelling evidence that PGPR promotes plant growth [67].

Therefore, PGPR can stimulate plant growth and enhance the plants' resistance to unfavorable conditions by intensifying physiological activity and regulating nutrient equilibrium [68]. This will provide favorable resources for the restoration of forage affected by extreme weather conditions in the Qinghai-Tibetan Plateau.

5. Conclusions

In this investigation, five distinct plant growth-promoting rhizobacteria (PGPR) strains (S1, S2, S3, S4, and S5) were isolated and characterized for their potent growth-enhancing capabilities. Subsequent pot experiments confirmed that these PGPR strains significantly bolstered the growth of *Elymus nutans* Griseb (*En*G), concurrently enhancing its nutrient utilization efficiency and cold tolerance. The presence of PGPR in the substrate notably elevated *En*G's plant height and dry weight, as well as its concentrations of soluble sugars and proteins, when compared to substrates devoid of PGPR. Furthermore, substrates amended with both PGPR and fertilizer demonstrated a marked increase in *En*G's apparent nutrient recovery efficiency, suggesting that PGPR strains are efficacious in optimizing plant nutrient uptake, thereby mitigating the necessity for excessive fertilizer application. These outcomes furnish valuable strain resources and foundational data for the formulation of specialized microbial fertilizers tailored for the alpine meadows of the Qinghai-Tibet Plateau.

Author contributions

All authors listed have made a substantial, direct, and intellectual contribution to the work and approved it for publication.

Funding

This work was financially supported by the Science and Technology Program of Tibet Autonomous Region (XZ202201ZY0005N), Sichuan Natural Science Foundation Project (2024NSFSC2074), China

Forestry and Grassland Reform and Development Fund (GZFCG2023-17620), and Innovation Team Funds of China West Normal University (KCXTD2023-5). We also thank the anonymous reviewers for providing critical comments and suggestions that improved the manuscript.

Conflict of interest

The authors declare that the research was conducted in the absence of any commercial or financial relationships that could be construed as a potential conflict of interest.

Reference

1. Raina J-B, Eme L, Pollock FJ, Spang A, Archibald JM, Williams TA. Symbiosis in the microbial world: from ecology to genome evolution. Biol Open. 2018;7: bio032524. doi:10.1242/bio.032524
2. Smith D, Praslickova D, Ilangumaran G. Inter-organismal signaling and management of the phytomicrobiome. Frontiers in Plant Science. 2015;6. doi:10.3389/fpls.2015.00722
3. FAN X-H, ZHANG S-A, MO X-D, LI Y-C, FU Y-Q, Liu Z. Effect of PGPR and N Source on Plant Growth and N, P Uptake by Tomato Grown in Calcareous Soils. Pedosphere. 2017;27. doi:10.1016/S1002-0160(17)60379-5
4. Vessey JK. Plant growth promoting rhizobacteria as biofertilizers. Plant and Soil. 2003;255: 571–586. doi:10.1023/A:1026037216893
5. Santoyo G, Urtis-Flores CA, Loeza-Lara PD, Orozco-Mosqueda MDC, Glick BR. Rhizosphere Colonization Determinants by Plant Growth-Promoting Rhizobacteria (PGPR). Biology (Basel). 2021;10: 475. doi:10.3390/biology10060475
6. Wu SC, Cao ZH, Li ZG, Cheung KC, Wong MH. Effects of biofertilizer containing N-fixer, P and K solubilizers and AM fungi on maize growth: a greenhouse trial. Geoderma. 2005;125: 155–166. doi:10.1016/j.geoderma.2004.07.003
7. Elhaissoufi W, Ghoulam C, Barakat A, Zeroual Y, Bargaz A. Phosphate bacterial solubilization: A key rhizosphere driving force enabling higher P use efficiency and crop productivity. Journal of Advanced Research. 2022;38: 13–28. doi:10.1016/j.jare.2021.08.014
8. El-Tohamy W, El-Abagy H, El-Greadly N, Gruda N. Hormonal changes, growth and yield of tomato plants in response to chemical and bio-fertilization application in sandy soils. Journal of Applied Botany and Food Quality. 2008;82.
9. Zhang H, Zhu J, Gong Z, Zhu J-K. Abiotic stress responses in plants. Nat Rev Genet. 2022;23: 104–119. doi:10.1038/s41576-021-00413-0
10. Baum C. Interactive effects of plant growth-promoting rhizobacteria and organic fertilization on P nutrition of Zea mays L. and Brassica napus L. Journal of Plant Nutrition and Soil Science. 2011 [cited 12 Jul 2024]. Available: https://www.academia.edu/20876205/Interactive_effects_of_plant_growth_promoting_rhizobacteria_and_organic_fertilization_on_P_nutrition_of_Zea_mays_L_and_Brassica_napus_L
11. Jha Y, Mohamed HI. Inoculation with Lysinibacillus fusiformis Strain YJ4 and Lysinibacillus sphaericus Strain YJ5 Alleviates the Effects of Cold Stress in Maize Plants. Gesunde Pflanzen. 2023;75: 77–95. doi:10.1007/s10343-022-00666-7
12. Kumari R, Pandey E, Bushra S, Faizan S, Pandey S. Plant Growth Promoting Rhizobacteria (PGPR) induced protection: A plant immunity perspective. Physiol Plant. 2024;176: e14495. doi:10.1111/ppl.14495
13. Rizvi A, Chandrawal R, Khan MohdH, Ahmed B, Umar S, Khan MohdS. Microbiological Control of Xanthomonas Induced Bacterial Leaf Streak Disease of Wheat via Phytocompounds and ROS Processing Enzymes Produced Under Biotic Stress. J Plant Growth Regul. 2024;43: 601–623. doi:10.1007/s00344-023-11119-4
14. Ahmad M, Imtiaz M, Nawaz MS, Mubeen F, Sarwar Y, Hayat M, et al. Thermotolerant PGPR consortium B3P

modulates physio-biochemical and molecular machinery for enhanced heat tolerance in maize during early vegetative growth. Annals of Microbiology. 2023;73: 34. doi:10.1186/s13213-023-01736-5
15. Guo J, Bowatte S, Hou F. Diversity of endophytic bacteria and fungi in seeds of Elymus nutans growing in four locations of Qinghai Tibet Plateau, China. Plant Soil. 2021;459: 49–63. doi:10.1007/s11104-020-04608-y
16. Long J, Liu D, Qiao W, Wang Y, Miao Y, Baosai H. Response of Elymus nutans Griseb. seedling physiology and endogenous hormones to drought and salt stress. Sci Rep. 2024;14: 17810. doi:10.1038/s41598-024-68894-7
17. Quan X, Qiao Y, Chen M, Duan Z, Shi H. Comprehensive evaluation of the allelopathic potential of Elymus nutans. Ecology and Evolution. 2021;11: 12389–12400. doi:10.1002/ece3.7982
18. Li M, Yang J, Wang X, Li D, Zhang C, Tian Z, et al. Transcriptome profiles identify the common responsive genes to drought stress in two *Elymus* species. Journal of Plant Physiology. 2020;250: 153183. doi:10.1016/j.jplph.2020.153183
19. Agarwal P, Singh PC, Chaudhry V, Shirke PA, Chakrabarty D, Farooqui A, et al. PGPR-induced OsASR6 improves plant growth and yield by altering root auxin sensitivity and the xylem structure in transgenic Arabidopsis thaliana. J Plant Physiol. 2019;240: 153010. doi:10.1016/j.jplph.2019.153010
20. Gul F, Khan IU, Rutherford S, Dai Z-C, Li G, Du D-L. Plant growth promoting rhizobacteria and biochar production from Parthenium hysterophorus enhance seed germination and productivity in barley under drought stress. Front Plant Sci. 2023;14. doi:10.3389/fpls.2023.1175097
21. Landa BB, Montes-Borrego M, Navas-Cortés JA. Use of PGPR for Controlling Soilborne Fungal Pathogens: Assessing the Factors Influencing Its Efficacy. In: Maheshwari DK, editor. Bacteria in Agrobiology: Disease Management. Berlin, Heidelberg: Springer; 2013. pp. 259–292. doi:10.1007/978-3-642-33639-3_10
22. Singh R, Jha P. The PGPR Stenotrophomonas maltophilia SBP-9 Augments Resistance against Biotic and Abiotic Stress in Wheat Plants. Frontiers in Microbiology. 2017;8: 1945. doi:10.3389/fmicb.2017.01945
23. Zhang X, Yang Z, Wang L, Yue Y, Wang L, Xiulian Yang. The effects of plant growth-promoting rhizobacteria on plants under temperature stress:A meta-analysis. Rhizosphere. 2023;28: 100788. doi:10.1016/j.rhisph.2023.100788
24. Hardy RWF, Holsten RD, Jackson EK, Burns RC. The Acetylene-Ethylene Assay for N_2 Fixation: Laboratory and Field Evaluation. Plant Physiology. 1968;43: 1185–1207.
25. Murphy J, Riley JP. A modified single solution method for the determination of phosphate in natural waters. Analytica Chimica Acta. 1962;27: 31–36. doi:10.1016/S0003-2670(00)88444-5
26. Nomura H, Komori T, Uemura S, Kanda Y, Shimotani K, Nakai K, et al. Chloroplast-mediated activation of plant immune signalling in Arabidopsis. Nat Commun. 2012;3: 926. doi:10.1038/ncomms1926
27. Wang M, Tachibana S, Murai Y, Li L, Lau SYL, Cao M, et al. Indole-3-Acetic Acid Produced by Burkholderia heleia Acts as a Phenylacetic Acid Antagonist to Disrupt Tropolone Biosynthesis in Burkholderia plantarii. Sci Rep. 2016;6: 22596. doi:10.1038/srep22596
28. Weselowski B, Nathoo N, Eastman AW, MacDonald J, Yuan Z-C. Isolation, identification and characterization of Paenibacillus polymyxa CR1 with potentials for biopesticide, biofertilization, biomass degradation and biofuel production. BMC Microbiol. 2016;16: 244. doi:10.1186/s12866-016-0860-y
29. Galkiewicz JP, Kellogg CA. Cross-kingdom amplification using bacteria-specific primers: complications for studies of coral microbial ecology. Appl Environ Microbiol. 2008;74: 7828–7831. doi:10.1128/AEM.01303-08
30. Johnson JS, Spakowicz DJ, Hong B-Y, Petersen LM, Demkowicz P, Chen L, et al. Evaluation of 16S rRNA gene sequencing for species and strain-level microbiome analysis. Nat Commun. 2019;10: 5029. doi:10.1038/s41467-019-13036-1
31. Wang M, Tachibana S, Murai Y, Li L, Lau SYL, Cao M, et al. Correction: Corrigendum: Indole-3-Acetic Acid Produced by Burkholderia heleia Acts as a Phenylacetic Acid Antagonist to Disrupt Tropolone Biosynthesis in Burkholderia plantarii. Sci Rep. 2016;6: 26217. doi:10.1038/srep26217
32. Vijay S, Nair RR, Sharan D, Jakkala K, Ajitkumar P. Percoll discontinuous density gradient centrifugation method for the fractionation of the subpopulations of *Mycobacterium smegmatis* and *Mycobacterium tuberculosis* from *in vitro* cultures. MethodsX. 2023;11: 102344. doi:10.1016/j.mex.2023.102344
33. Zhao X, Zhong Y, Zhou W. Molecular mechanisms of mesocotyl elongation induced by brassinosteroid in maize under deep-seeding stress by RNA-sequencing, microstructure observation, and physiological metabolism. Genomics. 2021;113: 3565–3581. doi:10.1016/j.ygeno.2021.08.020
34. Xu F, Chu C, Xu Z. Effects of different fertilizer formulas on the growth of loquat rootstocks and stem lignification. Sci Rep. 2020;10: 1033. doi:10.1038/s41598-019-57270-5
35. Gurrieri L, Merico M, Trost P, Forlani G, Sparla F. Impact of Drought on Soluble Sugars and Free Proline Content in Selected Arabidopsis Mutants. Biology. 2020;9. doi:10.3390/biology9110367

36. Kielkopf CL, Bauer W, Urbatsch IL. Bradford Assay for Determining Protein Concentration. Cold Spring Harb Protoc. 2020;2020: 102269. doi:10.1101/pdb.prot102269
37. Isfan D. Nitrogen physiological efficiency index in some selected spring barley cultivars. Journal of Plant Nutrition. 1990;13: 907–914. doi:10.1080/01904169009364125
38. Novoa R, Loomis RS. Nitrogen and plant production. Plant and Soil. 1981;58: 177–204. doi:10.1007/BF02180053
39. Fageria NK, Baligar VC. Enhancing Nitrogen Use Efficiency in Crop Plants. Advances in Agronomy. Academic Press; 2005. pp. 97–185. doi:10.1016/S0065-2113(05)88004-6
40. Olasupo IO, Liang Q, Zhang C, Islam MS, Li Y, Yu X, et al. Agronomic Biofortification of Cayenne Pepper Cultivars with Plant Growth-Promoting Rhizobacteria and Chili Residue in a Chinese Solar Greenhouse. Microorganisms. 2021;9: 2398. doi:10.3390/microorganisms9112398
41. Aw X, Z L, Wc L, Zh Y. The effect of plant growth-promoting rhizobacteria (PGPR) on arsenic accumulation and the growth of rice plants (Oryza sativa L.). Chemosphere. 2020;242: 125136. doi:10.1016/j.chemosphere.2019.125136
42. Dhar Purkayastha G, Mangar P, Saha A, Saha D. Evaluation of the biocontrol efficacy of a Serratia marcescens strain indigenous to tea rhizosphere for the management of root rot disease in tea. PLoS One. 2018;13: e0191761. doi:10.1371/journal.pone.0191761
43. Ansari FA, Ahmad I. Fluorescent Pseudomonas -FAP2 and Bacillus licheniformis interact positively in biofilm mode enhancing plant growth and photosynthetic attributes. Sci Rep. 2019;9: 4547. doi:10.1038/s41598-019-40864-4
44. del Rosario Cappellari L, Santoro MV, Reinoso H, Travaglia C, Giordano W, Banchio E. Anatomical, morphological, and phytochemical effects of inoculation with plant growth- promoting rhizobacteria on peppermint (Mentha piperita). J Chem Ecol. 2015;41: 149–158. doi:10.1007/s10886-015-0549-y
45. Khoso MA, Wagan S, Alam I, Hussain A, Ali Q, Saha S, et al. Impact of plant growth-promoting rhizobacteria (PGPR) on plant nutrition and root characteristics: Current perspective. Plant Stress. 2024;11: 100341. doi:10.1016/j.stress.2023.100341
46. Guan H, Liu X, Fu Y, Han X, Wang Y, Li Q, et al. The locoweed endophyte Alternaria oxytropis affects root development in Arabidopsis in vitro through auxin signaling and polar transport. J Exp Bot. 2023;74: 931–944. doi:10.1093/jxb/erac430
47. Fan H, Wu J, Liu W, Yuan Y, Hu L, Cai Q. Linkages of plant and soil C:N:P stoichiometry and their relationships to forest growth in subtropical plantations. Plant Soil. 2015;392: 127–138. doi:10.1007/s11104-015-2444-2
48. Mantelin S, Touraine B. Plant growth-promoting bacteria and nitrate availability: impacts on root development and nitrate uptake. Journal of Experimental Botany. 2004;55: 27–34. doi:10.1093/jxb/erh010
49. Sun W, Shahrajabian MH. Biostimulant and Beyond: Bacillus spp., the Important Plant Growth-Promoting Rhizobacteria (PGPR)-Based Biostimulant for Sustainable Agriculture. Earth Syst Environ. 2025 [cited 27 Jan 2025]. doi:10.1007/s41748-024-00552-4
50. Ruiz Herrera LF, Shane MW, López-Bucio J. Nutritional regulation of root development. Wiley Interdiscip Rev Dev Biol. 2015;4: 431–443. doi:10.1002/wdev.183
51. Li X, Zeng R, Liao H. Improving crop nutrient efficiency through root architecture modifications. Journal of Integrative Plant Biology. 2016;58: 193–202. doi:10.1111/jipb.12434
52. Das PP, Singh KR, Nagpure G, Mansoori A, Singh RP, Ghazi IA, et al. Plant-soil-microbes: A tripartite interaction for nutrient acquisition and better plant growth for sustainable agricultural practices. Environ Res. 2022;214: 113821. doi:10.1016/j.envres.2022.113821
53. Egamberdiyeva D. The effect of plant growth promoting bacteria on growth and nutrient uptake of maize in two different soils. Applied Soil Ecology. 2007;36: 184–189. doi:10.1016/j.apsoil.2007.02.005
54. Krey T, Caus M, Baum C, Ruppel S, Eichler-Löbermann B. Interactive effects of plant growth–promoting rhizobacteria and organic fertilization on P nutrition of Zea mays L. and Brassica napus L. Journal of Plant Nutrition and Soil Science. 2011;174: 602–613. doi:10.1002/jpln.200900349
55. Wei T, Wang Y, Xie Z, Guo D, Chen C, Fan Q, et al. Enhanced ROS scavenging and sugar accumulation contribute to drought tolerance of naturally occurring autotetraploids in Poncirus trifoliata. Plant Biotechnol J. 2019;17: 1394–1407. doi:10.1111/pbi.13064
56. Selvarajan D, Mohan C, Dhandapani V, Nerkar G, Jayanarayanan AN, Vadakkancherry Mohanan M, et al. Differential gene expression profiling through transcriptome approach of Saccharum spontaneum L. under low temperature stress reveals genes potentially involved in cold acclimation. 3 Biotech. 2018;8: 195. doi:10.1007/s13205-018-1194-2
57. Janská A, Marsík P, Zelenková S, Ovesná J. Cold stress and acclimation - what is important for metabolic adjustment?

Plant Biol (Stuttg). 2010;12: 395–405. doi:10.1111/j.1438-8677.2009.00299.x
58. Batool T, Ali S, Seleiman MF, Naveed NH, Ali A, Ahmed K, et al. Plant growth promoting rhizobacteria alleviates drought stress in potato in response to suppressive oxidative stress and antioxidant enzymes activities. Sci Rep. 2020;10: 16975. doi:10.1038/s41598-020-73489-z
59. Bruno B, Karthik C, Ma Y, Kadirvelu K, Freitas H, Rajkumar M. Amelioration of chromium and heat stresses in Sorghum bicolor by Cr6+ reducing-thermotolerant plant growth promoting bacteria. Chemosphere. 2020;244: 125521. doi:10.1016/j.chemosphere.2019.125521
60. Patani A, Prajapati D, Ali D, Kalasariya H, Yadav VK, Tank J, et al. Evaluation of the growth-inducing efficacy of various Bacillus species on the salt-stressed tomato (Lycopersicon esculentum Mill.). Front Plant Sci. 2023;14. doi:10.3389/fpls.2023.1168155
61. Zhuoma P, Tondrob D, Qunpei T, Fu J, Dan S. Muti-omics revealed the mechanisms of MT-conferred tolerance of Elymus nutans Griseb. to low temperature at XiZang. BMC Plant Biology. 2024;24: 901. doi:10.1186/s12870-024-05583-7
62. Long J, Gao X, Miao Y. Effects of environmental factors on the phenotypic traits and seed element accumulation of wild Elymus nutans in Tibet. Sci Rep. 2025;15: 1838. doi:10.1038/s41598-025-85415-2
63. Theocharis A, Clément C, Barka EA. Physiological and molecular changes in plants grown at low temperatures. Planta. 2012;235: 1091–1105. doi:10.1007/s00425-012-1641-y
64. Yamori N, Levine CP, Mattson NS, Yamori W. Optimum root zone temperature of photosynthesis and plant growth depends on air temperature in lettuce plants. Plant Mol Biol. 2022;110: 385–395. doi:10.1007/s11103-022-01249-w
65. Luo H, Xu H, Chu C, He F, Fang S. High Temperature can Change Root System Architecture and Intensify Root Interactions of Plant Seedlings. Front Plant Sci. 2020;11. doi:10.3389/fpls.2020.00160
66. Bruno LB, Karthik C, Ma Y, Kadirvelu K, Freitas H, Rajkumar M. Amelioration of chromium and heat stresses in *Sorghum bicolor* by Cr6+ reducing-thermotolerant plant growth promoting bacteria. Chemosphere. 2020;244: 125521. doi:10.1016/j.chemosphere.2019.125521
67. Zhao X, Yuan X, Xing Y, Dao J, Zhao D, Li Y, et al. A meta-analysis on morphological, physiological and biochemical responses of plants with PGPR inoculation under drought stress. Plant, Cell & Environment. 2023;46: 199–214. doi:10.1111/pce.14466
68. Franco-Franklin V, Moreno-Riascos S, Ghneim-Herrera T. Are Endophytic Bacteria an Option for Increasing Heavy Metal Tolerance of Plants? A Meta-Analysis of the Effect Size. Front Environ Sci. 2021;8. doi:10.3389/fenvs.2020.603668

Table 1 Growth promoting characteristics of four PGPR strains

Strains	Nitrogen fixation capacity (μg mL^{-1})	Inorganic phosphorus solubilization capacity (μg mL^{-1})	Organic phosphorus solubilization capacity (μg mL^{-1})	Potassium-solubilising capacity (μg mL^{-1})	IAA contents (μg mL^{-1})
S1	5.10±0.07	176.2±62.46	24.73±15.91	4.42±0.34	596.87±63.7
S2	-	136.32±68.19	79.85±5.34	11.42±0.59	197.10±22.80
S3	2.08±0.06	250.24±30.11	44.52±26.51	8.18±0.42	193.32±7.17
S4	5.06±0.02	172.09±31.22	47.34±4.24	6.05±0.35	168.77±21.35
S5	5.57±0.03	311.95±50.06	49.46±5.61	4.59±0.44	166.64±16.88

Table 2 The main effects of temperature (T), PGPR, and fertilizers (NPK) on *Elymus nutans Griseb*. The values in the table represent the statistical significance (p - values) of the effects of different sources of variation on each trait. PT: plant height; DW: dry weight, Chl: plant chlorophyll; RL: root length; RB: number of root branches; RSA: root surface area; RD: root average diameter; SS: soluble sugars; SP: soluble proteins.

**Source of variation**	**PT**	**DW**	**Chl**	**RL**	**RB**	**RSA**	**RD**	**SS**	**SP**
**T**	<0.001	<0.001	<0.001	<0.001	0.076	<0.001	<0.001	<0.001	<0.001
**NPK**	<0.001	<0.001	<0.001	<0.001	<0.001	<0.001	0.847	<0.001	<0.001
**PGPR**	<0.001	<0.001	<0.001	<0.001	<0.001	0.007	<0.001	<0.001	<0.001
**T* NPK**	0.333	0.007	<0.001	0.429	0.893	0.012	0.015	<0.001	<0.001
**T* PGPR**	0.629	0.028	<0.001	<0.001	<0.001	0.392	<0.001	<0.001	<0.001
**NPK* PGPR**	0.386	0.007	<0.001	0.174	0.949	0.728	<0.001	<0.001	<0.001
**T* NPK* PGPR**	0.290	0.026	<0.001	0.020	0.898	0.010	0.181	<0.001	<0.001

Note： When $p < 0.05$, it shows significant impact. When $p < 0.01$, it signifies an extremely remarkable influence.

Fig.1. General location and climatic parameters of three sampling sites. Three randomly selected areas where *Elymus nutans Griseb* was growing vigorously. The map, base map, shape files, and map data were obtained from the National Geographic Information Public Service Platform "TianDiTu" (https://cloudcenter.tianditu.gov.cn/dataSource), the specific URL of the web page for retrieving this data is: https: //cloudcenter.tianditu.gov.cn/administrativeDivision/ . The map base layer has been reviewed and approved with the approval number GS (2024) 0650. The base map is unmodified.

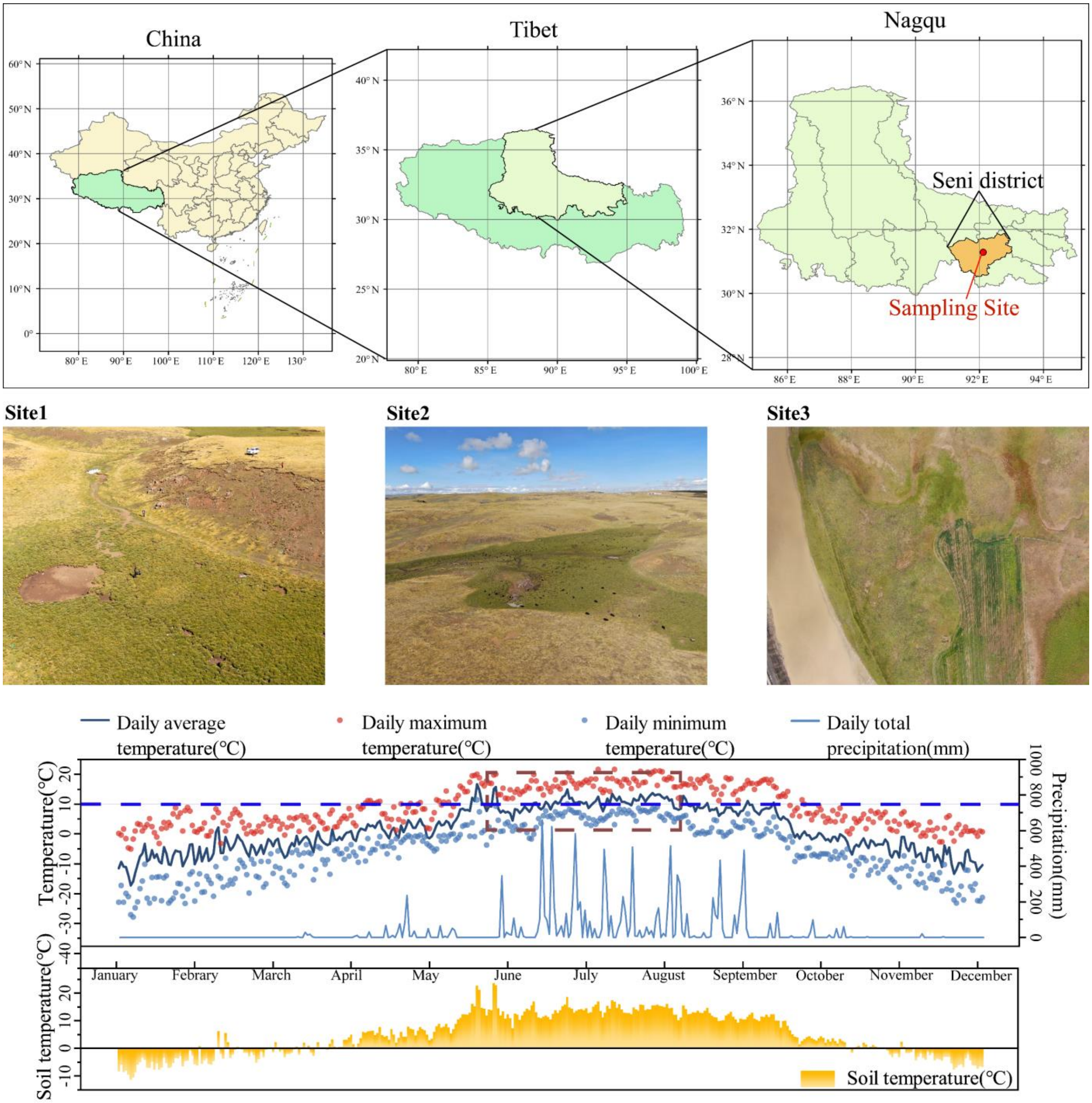

Fig.2. The effects of different treatments on the growth of *En*G at different temperatures. T1(addition of PGPR and no addition of NPK fertilizers), T2 (neither PGPR nor NPK fertilizers), T3 (addition of PGPR and NPK fertilizers), and T4 (no addition of PGPR, but addition of NPK fertilizers).

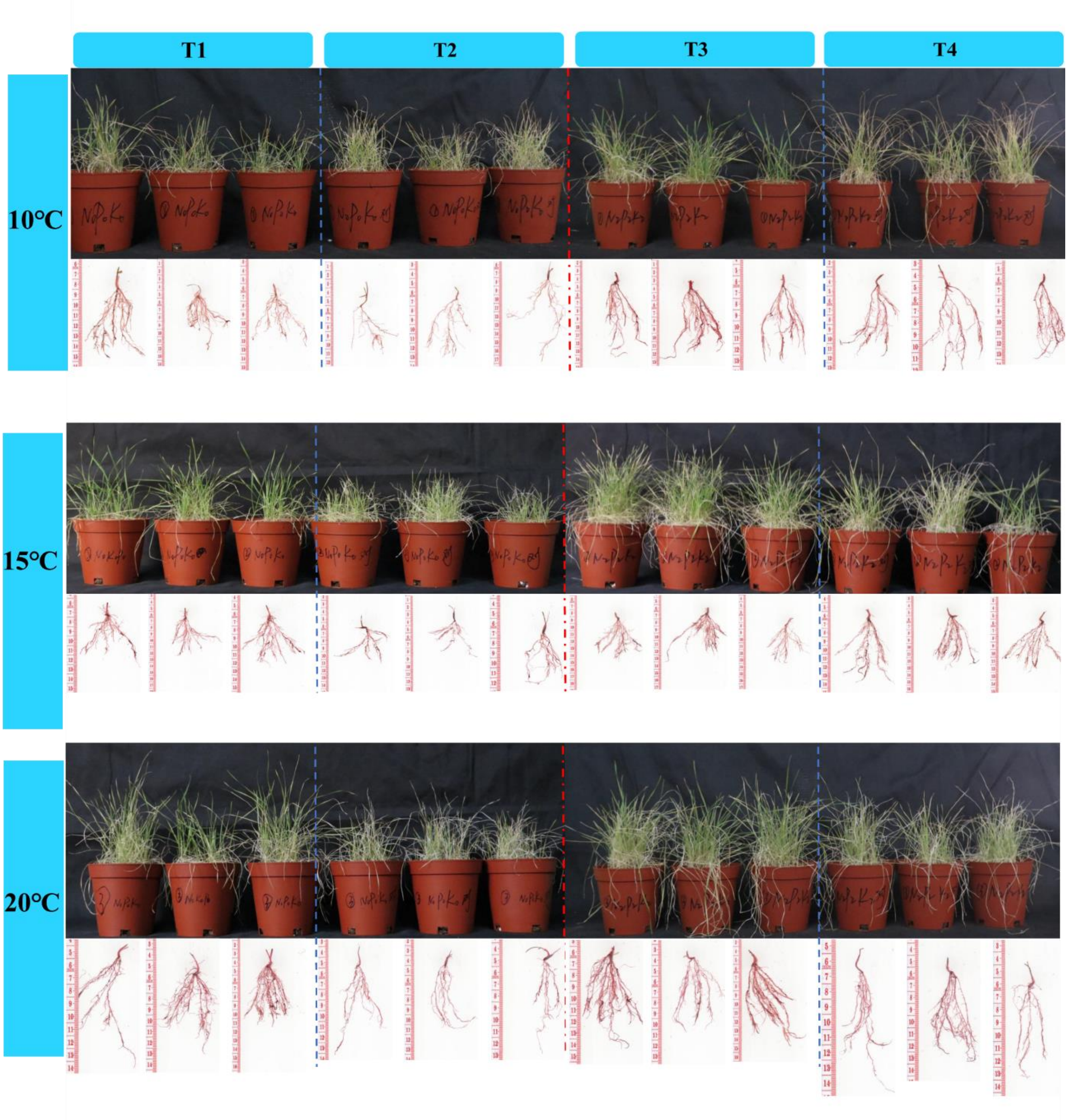

Fig.3. Effect of different treatments on plant height (PT), dry weight (DW), and plant chlorophyll (Chl)content. Lowercase letters indicate differences between treatments and uppercase letters indicate differences between temperatures.

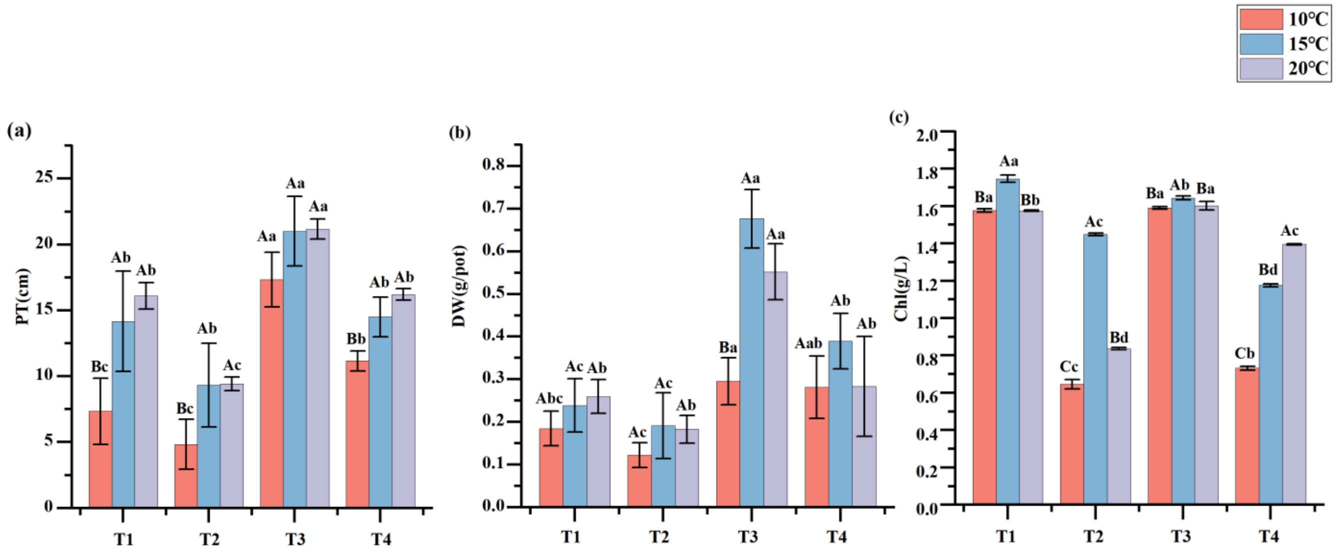

Fig.4. Effect of different treatments on root length (RL), number of root branches (RB), root surface area (RSA), and root average diameter (RD). Lowercase letters indicate differences between treatments and uppercase letters indicate differences between temperatures.

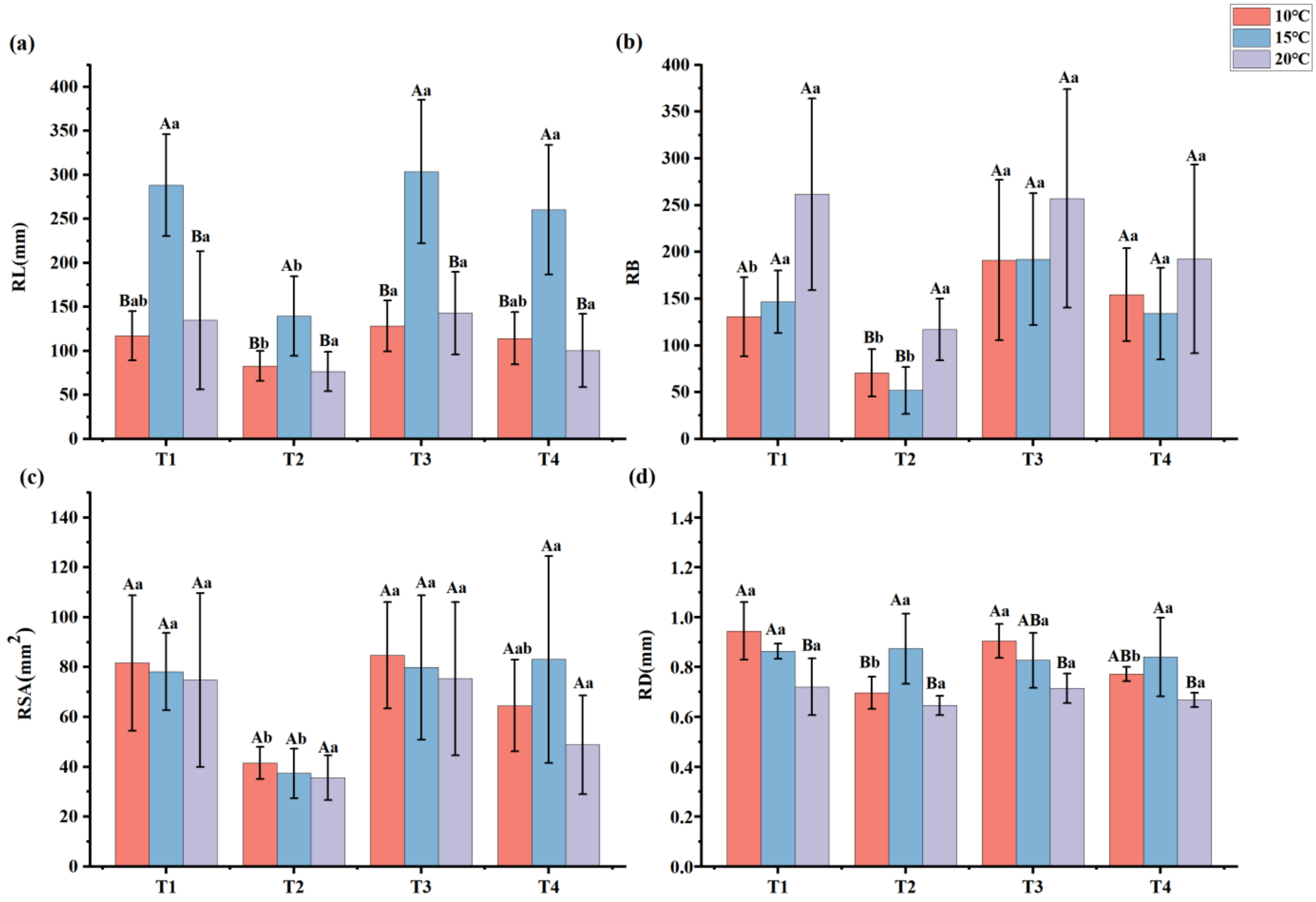

Fig.5. Effect of different treatments on soluble sugars (SS), and soluble proteins (SP)content. Lowercase letters indicate differences between treatments and uppercase letters indicate differences between temperatures.

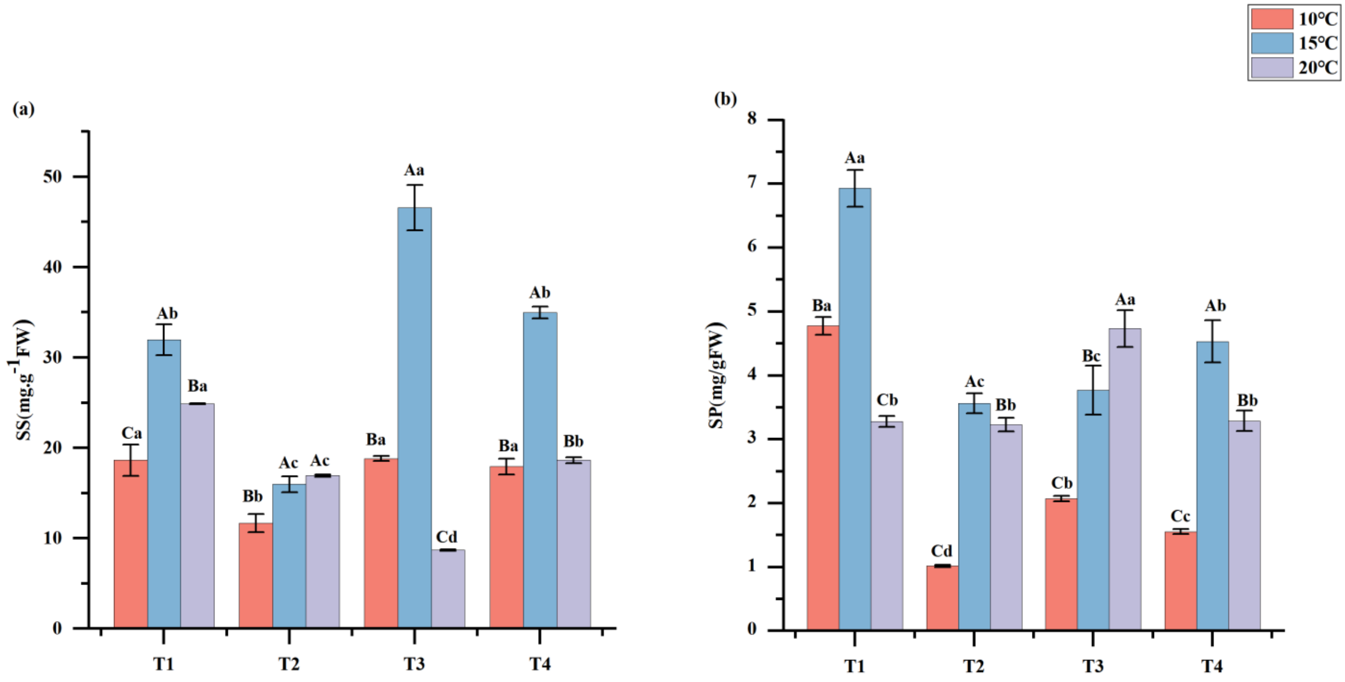

Fig.6. Effect of different treatments (T3 and T4) on physiological efficiency of nitrogen (NPE), phosphorus (PPE), potassium (KPE), agronomic efficiency of nitrogen (NAE), phosphorus (PAE) and potassium (KAE), and apparent recovery efficiency of nitrogen (NRE), phosphorus (PRE) and potassium (KRE) content. * means $p < 0.05$, ** means $p < 0.01$, *** means $p < 0.001$, ns means no significant difference.

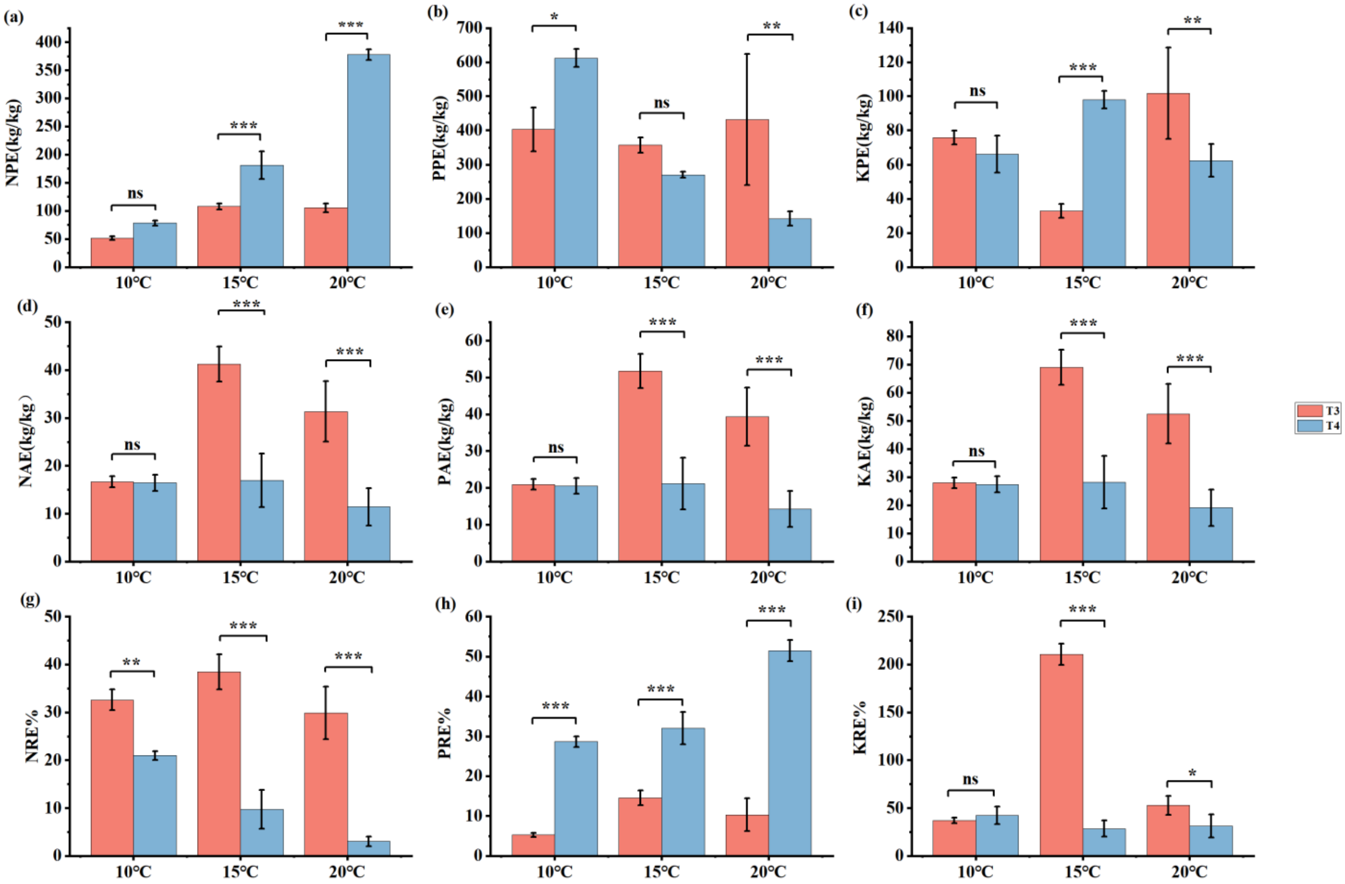

Fig.7. (a) Spearman's correlation between the relative abundance of PE, AE, and RE, and Plant growth factors. *, **, and *** represent significant correlations at the 0.05, 0.01, and 0.001 levels. (b) Mantel test between nutrient utilization and indicators of plant growth. PT, plant height; DW, dry weight; Chl, plant chlorophyll; RL, root length; RB, number of root branches; RSA, root surface area; RD, root average diameter; SS, soluble sugars; SP, soluble proteins.

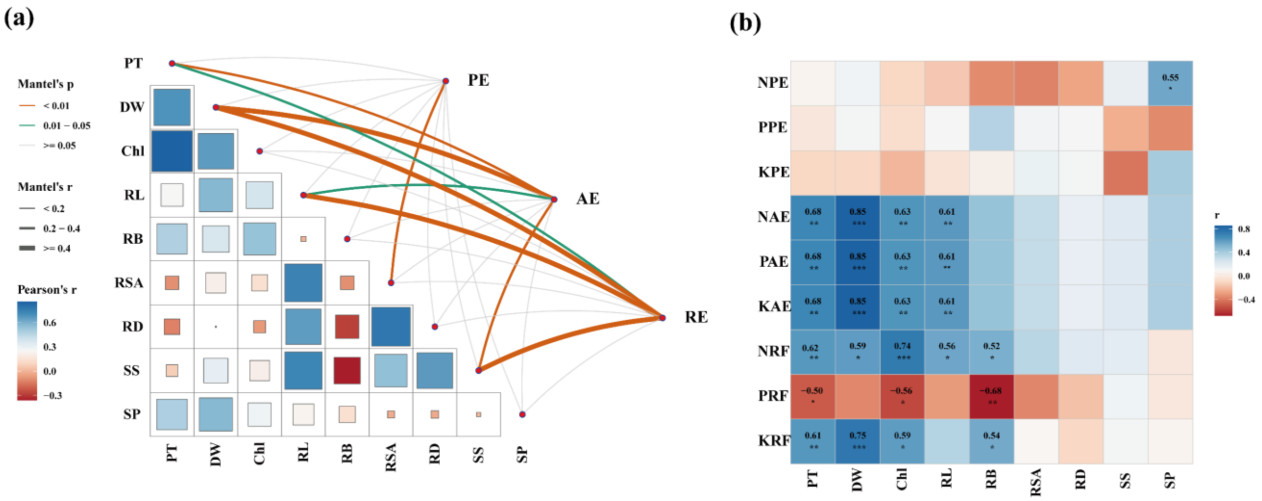

Response to Reviewers

Manuscript Number: **PONE-D-24-56644**

Mechanisms of growth promotion and resistance of PGPR to *Elymus nutans* Griseb at different temperatures

Dear Editor and Reviewers,

Thank you very much for your comments and professional advice. These opinions help to improve academic rigor of our article. Based on your suggestion and request we have made corrected modifications on the revised manuscript. We hope that our work can be improved again. We would like to show the details as follows:

Journal Requirements:

Via meticulous inspection and comprehensive review, we have ascertained that our manuscript adheres to the stylistic requisites of PLOS ONE, encompassing the stipulations for file naming. We have successfully applied for an ORCID iD and have accomplished its verification within the Editorial Manager. In relation to the matter of our work permit, it should be noted that all our undertakings, spanning experimental procedures and the writing process, were carried out within the confines of the laboratory affiliated with our primary institution, thereby obviating the need for any additional permits. What distinguishes our work is the fact that the funder assumes the role of a co-collaborator in this study. The funder actively participated in the study design, data gathering and analytical processes, as well as in making the decision regarding publication and manuscript compilation. Finally, with respect to the concern surrounding Figure 1, the geographical information map, we hereby affirm that we have refrained from utilizing any copyrighted graphic or textual materials published by third parties. We confirm that all images included in our submission can be published under the CC BY 4.0 license as required by PLOS ONE. The base maps of our geographical information were exclusively derived from publicly accessible information disclosed on the website: https://www.tianditu.gov.cn/, the specific URL of the web page for retrieving this data is: https://cloudcenter.tianditu.gov.cn/dataSource. The map, base map, shape files, and map data in Figure 1 were obtained from the National Geographic Information Public Service Platform "TianDiTu"(https://cloudcenter.tianditu.gov.cn/administrativeDivision/). The data was collected in 2024, the map base layer has been reviewed and approved with the approval number GS (2024) 0650. The TianDiTu website offers a variety of map services and data resources. The base map data downloaded on this platform is in GeoJSON format. We promise that the base map has not been modified. We accessed and utilized the data in strict accordance with the platform's regulations (https://www.tianditu.gov.cn/about/copyright). The software used to create these maps is ArcGIS 10.8. If you need the source files of the map base layers we used, we will also upload them to your journal's system in the form of attachments. Additionally, we collected rhizosphere soil from the high-altitude pastoral areas where Elymus nutans is well adapted for strain isolation and identification, and we also studied the plant-growth-promoting properties of the strains. The general information about the sampling site (geographical coordinates, vegetation type, and climatic characteristics) has been described in detail in the Materials and Methods section. If our modification of the location map does not meet your requirements and the journal's standards, we can omit the location map and provide an alternative figure that only retains the meteorological map. We truly appreciate your invaluable comments and extend our gratitude for your diligent efforts.

Figure 1:

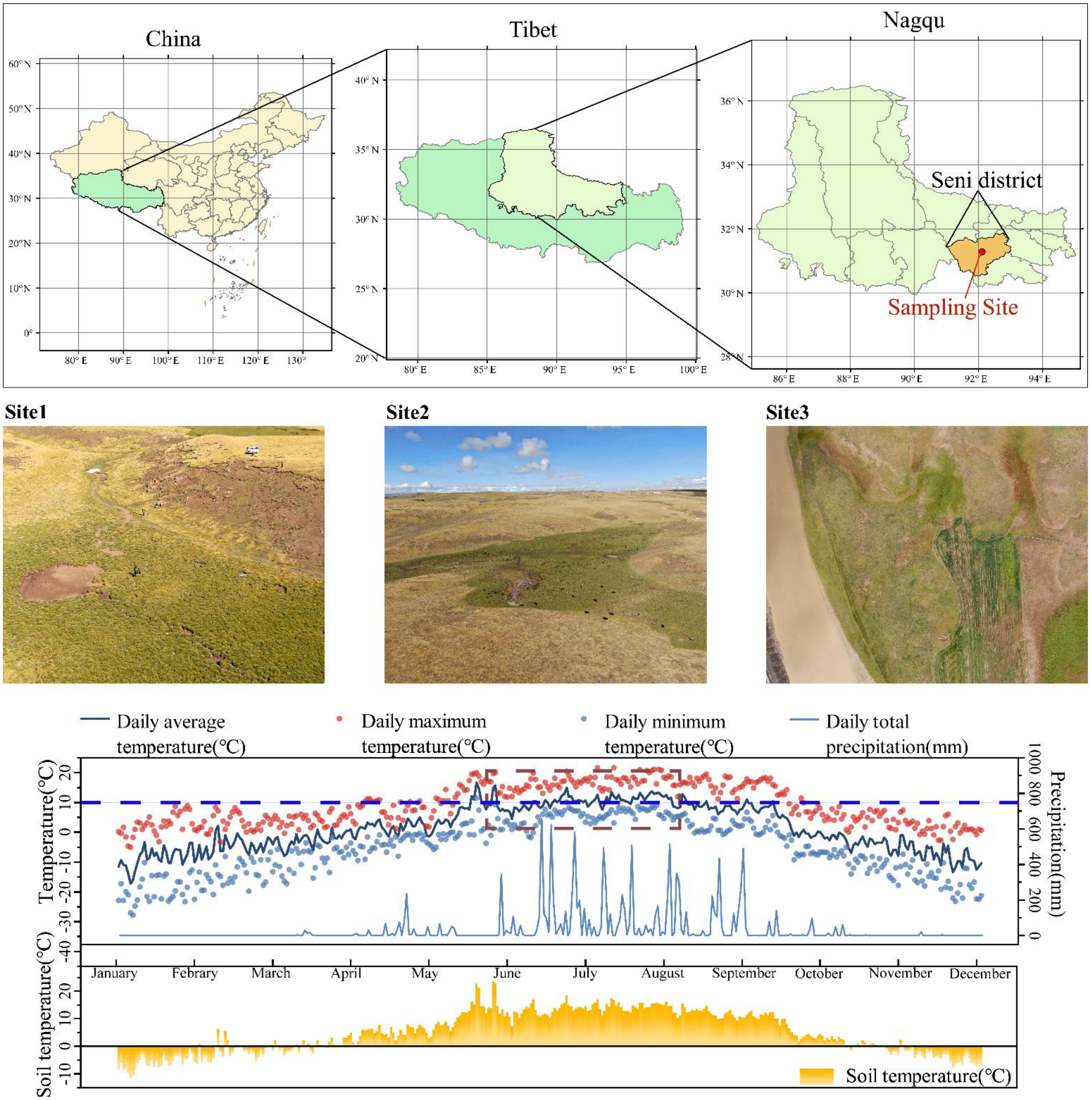
China
Tibet
Nagqu
Seni district
Sampling Site
Site1
Site2
Site3
Daily average temperature(℃)
Daily maximum temperature(℃)
Daily minimum temperature(℃)
Daily total precipitation(mm)
Temperature(℃)
Precipitation(mm)
January
Febrary
March
April
May
June
July
August
September
October
November
December
Soil temperature(℃)

Alternative for Figure 1:

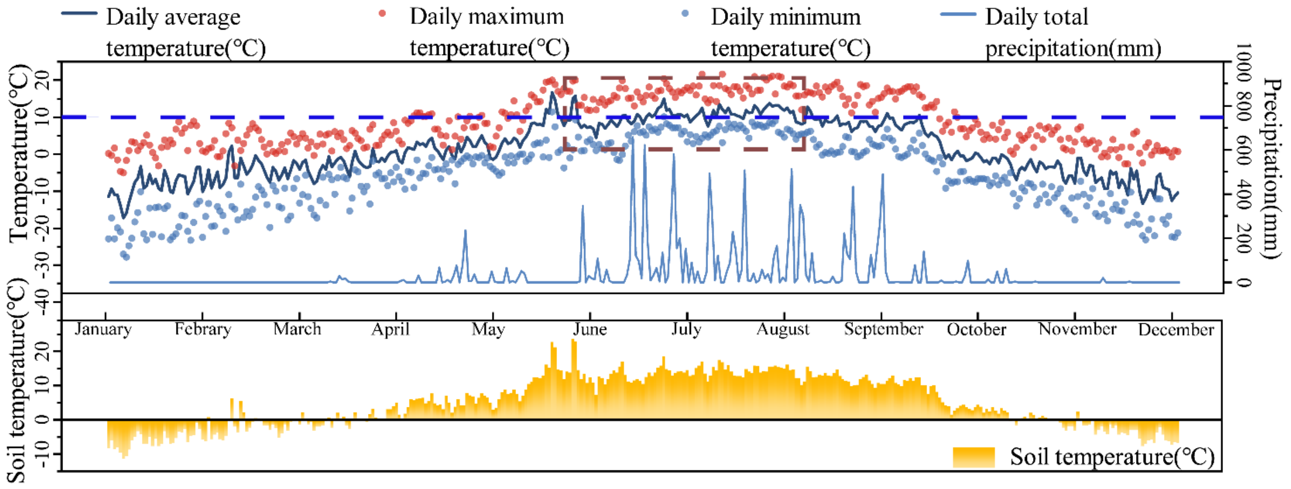

Reviewer #2:

1.This is a valuable study as strains were obtained and characterized from the Tibetan Plateau, a unique and understudied ecosystem. The potential use of plant growth promoting bacteria in pasture production in this ecosystem is an interesting study area. However, it seems to me that the tested conditions (fertilizer scheme, substrate and temperature conditions) are not relevant to the application area, the Tibetan Plateau. Therefore, the results of this study are far less relevant and do not support the stated conclusions.

Answer: Thank you for the reviewer's comments. The fertilizer method of this study refers to the "3414" design, which determines the optimum dosage and ratio of N-P-K fertilizers at the "2" level of N, P and K fertilizers. Its purpose is to explore the impact of PGPR on the nutrient utilization of *Elymus nutans* Griseb. The substrate conditions for this study were formulated with reference to the soil characteristics of the sampling sites. The temperature conditions in the potting experiments in this study were designed based on the temperature gradient during the growing season of *Elymus nutans* Griseb on the Tibetan Plateau. To this we add in materials and methods. In addition, we have added a graph of the annual temperature on the Qinghai - Tibet Plateau to Figure 1, and the temperature range during the growing season of *Elymus nutans* Griseb (June, July, and August) is circled with a red box.

The four treatments set up in this study were T1(addition of PGPR and no addition of NPK fertilizers), T2 (neither PGPR nor NPK fertilizers), T3 (addition of PGPR and NPK fertilizers), and T4 (no addition of PGPR, but addition of NPK fertilizers) at three replicates. The NPK represents applied rates of 150 mg kg^{-1}, 120 mg kg^{-1}, and 90 mg kg^{-1} of urea (N,46.65%), superphosphate (P_2O_5,12%) and potassiumchloride (K_2O,60%), respectively. Furthermore, according to the climatic temperature during the growing season

The culture substrate was formulated with reference to the soil characteristics of the sampling sites, which was composed of a blend of peat soil, vermiculite, perlite, and coir in a ratio of 4:1:1:1 (v/v), with 50 g per bag, which was then sterilized at 121 °C for 30 minutes. Subsequently, 78.2 g of the culture medium was combined with 1% (volume/volume) PGPR bacterial mixture and transferred into a 660 mL plastic container (70 × 95 × 98 mm) [30]. A cup with an equal volume of sterile water was used as the control.

Furthermore, according to the climatic temperature during the growing season (June, July, and August) of *En*G on the Tibetan Plateau (Fig. 1), this study was set up with temperature gradients of 10 °C, 15 °C, and 20 °C. Incubation was carried out in three incubators with a light (16 h)/dark (8 h) cycle (light at 350 μEm^{-2} s^{-1}) and 60% humidity. Plant seeds (wild *En*G seeds collected in Nagqhu, Tibet) were sterilized on the

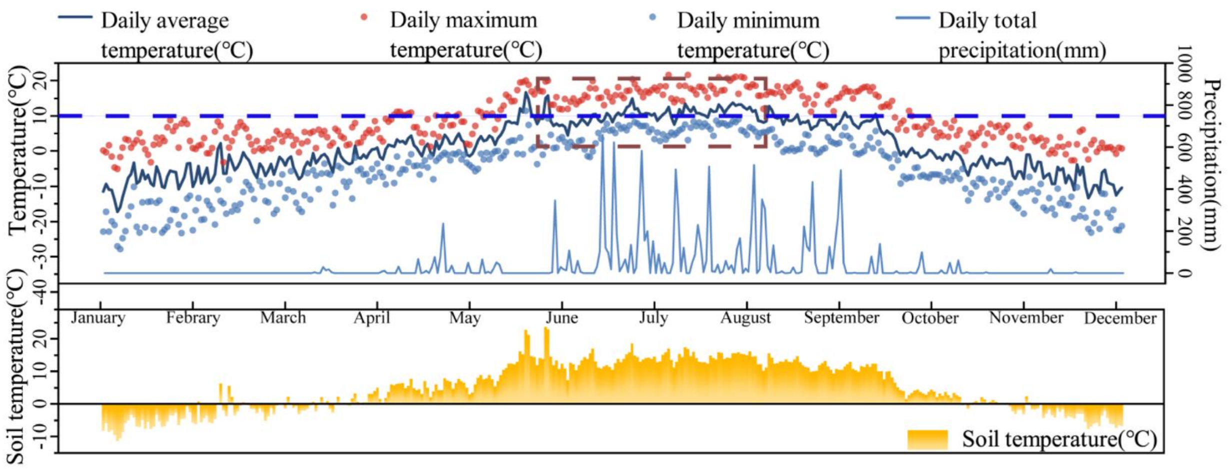

2. The results and discussion are very difficult to understand due to the use of many abbreviations, coded treatments and a lack of structure in the text. Therefore, I was also not able to verify the statistical analyses.

Answer: First of all, I would like to apologize for the use of abbreviations of words and the obscurity of the structure in the article. Perhaps this kind of writing may have a negative impact on the reading experience. During the writing of the article, as some professional terms are rather long, we noted the abbreviations after their full names when they first appeared. These abbreviations were also adopted in accordance with those used in currently published articles. Subsequently, we used these abbreviations throughout the rest of the article. We have also conducted checks on the language logic and structure. We sincerely apologize for any inconvenience caused to you. In addition, regarding the issue of statistical analysis, we have compiled and uploaded the original data for your reference. You can access it in Figshare (https://doi.org/10.6084/m9.figshare.27094078.v1). Thank you again for your valuable comments, and we hope you can forgive any inconvenience.

3. Important information is lacking in Material and methods, e.g. NFM-agar, brief descriptions of methods, the preparation of inoculum (CFU counts etc).

Answer: We supplemented the materials and methods and explained the NFM-agar as well as the concentration of bacterial agent (1×10^{8}CFU/ml).

Inoculate the purified strains onto Nitrogen free medium (NFM), Pikovskaya medium, Bacterial Organophosphorus medium, Potassium Bacteria medium and King's B medium respectively to determine whether the strains have growth - promoting abilities. Among them, 273 strains can grow on the selective medium. The ability of the isolates to grow on NFM agar and their nitrogen-fixing capacity was evaluated using the acetylene reduction method [24]. Strains' phosphorus solubilizing capacity was tested with Mo

bacterial agent with OD_{600} value of 0.6 (the concentration of the single bacterial agent is about 1×10^{8}CFU/ml). Equal amount of mixing to prepare a composite bacterial agent [30].

4. The manuscript does not disclose which five strains out of 273 strains were selected, and based on which criteria these were selected.

Answer: We supplemented materials regarding the origin of the 273 strains and screened for excellent strains by measuring their growth - promoting abilities. Through the determination of growth - promoting capabilities, we separately selected five strains with the highest nitrogen - fixing, phosphate - solubilizing, potassium - releasing, and IAA - secreting abilities for identification. The magnitudes of these abilities are shown in Table 1.

Inoculate the purified strains onto Nitrogen free medium (NFM), Pikovskaya medium, Bacterial Organophosphorus medium, Potassium Bacteria medium and King's B medium respectively to determine whether the strains have growth - promoting abilities. Among them, 273 strains can grow on the selective medium. The ability of the isolates to grow on NFM agar and their nitrogen-fixing capacity was evaluated using the acetylene reduction method [24]. Strains' phosphorus solubilizing capacity was tested with Mo

the supernatant was quantified using flame spectrophotometry [28]. Five excellent strains (S1, S2, S3, S4 and S5) with high growth promoting ability were screened by the determination of growth-promoting ability. All five strains solubilized inorganic and organic phosphates, decomposed K, and secreted IAA. In addition, S1, S3, S4, and S5 fixed N (Table 1).

Reviewer #3:

1. There is no experiment of mechanism of growth promotion and resistance of PGPR (eg. ROS, PO, PPO, PAL activity; amount of MDA, GSH, IAA, antioxidants, etc.) in this study. Here, title of this paper does not represent the results. This research is mainly focused on nutrient uptake efficiency of PGPR in peat soil based substrate with different temperature. In another words, authors should mention what and where is the PGPR mechanism in this study?

Answer: I deleted the word "mechanism" in the title, and changed the title of the manuscript to"Effect of PGPR on growth and cold tolerance of *Elymus nutans* Griseb at different temperatures".

Effect of PGPR on growth and nutrient utilization of *Elymus nutans* Griseb at different temperatures

Reviewer #4:

1. The abstract should be rewritten in a more systematic pattern to improve clarity and readability. Clarify the research aim, emphasizing the focus on temperature effects on PGPR-mediated plant growth and nutrient utilization. Specify the type of pot experiments conducted, including the temperature ranges used. Explain the significance of the treatments (T1, T2, T3, T4) briefly and consistently. Summarize the findings succinctly, focusing on the most significant results.

Answer: Thank you for your valuable comments. We have rewritten the abstract according to your suggestion. The purpose of the study was made clearer and the focus on the effect of temperature on PGPR-mediated plant growth and nutrient utilization was emphasized. The indoor pot experiment, the four treatments, and the temperature gradient settings are also clarified.

Abstract

In order to show the growth-promoting effects of PGPR (S1, S2, S3, S4 and S5) isolated from the rhizosphere soil of *Elymus nutans* Griseb (*En*G) on the Tibetan Plateau. In this study, we simulated three temperature gradients (10°C, 15°C, and 20°C) during the growing season of *Elymus nutans* Griseb on the Tibetan Plateau, and set up four incubation substrate treatments:T1(addition of PGPR and no addition of NPK fertilizers), T2 (neither PGPR nor NPK fertilizers), T3 (addition of PGPR and NPK fertilizers), and T4 (no addition of PGPR, but addition of NPK fertilizers), to explore the effects of PGPR on the growth and nutrient (NPK) utilization of *Elymus nutans* Griseb at different temperatures. The results showed that compared with T2, the plant height (PT) of T1 increased by 51.72% to 70.67%, the dry weight (DW) increased by 24.99% to 51.25%, the soluble sugar (SS) and soluble protein (SP) content significantly increased by 59.37% and 369.66% at 10 °C respectively ($p<0.05$), and significantly increased by 100.17% and 94.5% at 15 °C respectively ($p<0.05$). Compared with T4, T3 significantly decreased the physiological efficiency of N (NPE) at 15 °C and 20 °C by 40.43% and 72.11%, respectively. In summary, our results showed that this PGPR can promote the growth of *Elymus nutans* Griseb and improve its nutrient utilization efficiency.

2. The introduction section needs to incorporate more recent literature to provide a comprehensive background. In introduction section ([12] revealed that specific PGPR strains can impart cold resistance to rice plants, boosting their viability, fertility, and yield related traits and accelerating the progression of their reproductive cycle. Furthermore, variation in temperature tolerance exists among PGPR strains, affecting efficacy and plant health [13].), needs to rewrite.

Answer: We have revised the introductory section and incorporated the most recent literature.

management techniques, encompassing the use of fertilizers and external protective compounds, can reduce the harmful influence of cold stress on plant growth [10]. PGPR can enhance plant nutrient uptake and accessibility via biochemical and physiological processes [11], and also enhance plant stress tolerance through systemic resistance induction, producing phytohormones, solubilizing nutrients [12,13]. Nevertheless, there are significant differences in temperature tolerance among different PGPR strains, and these differences directly affect their efficacy in plant growth [14]. Therefore, it is crucial to choose PGPR strains that are suitable for a particular crop and its environment.

3. In methodology section the sub-heading “Identification of PGPR strains” is written well but its too lengthy, make it

simple and short.

Answer: We have modified and adapted the methodology sections as appropriate and have simplified the identification of PGPR strains section as appropriate.

2.3. Identification of PGPR strains

Five strains (S1, S2, S3, S4, and S5) were inoculated in LB liquid medium for incubation at 37 °C under shaking (160 rpm) for 24 h. Bacterial genomic DNA was extracted from these strains using a commercial kit (Tiangen Biochemical Technology, Beijing, China). The 16S rRNA gene was polymerase chain reaction (PCR) amplified with the primer set 27 F (5′-AGAGTTTGATCCTGGCTCAG-3′) and 1492R (5′-GGTTACCTTGTTACGACTT-3′) as described by Galkiewicz and Kellogg (2008) [27]. PCR was executed in a 50 μL volume, comprising 1 μL of 5 μM forward primer, 1 μL of 5 μM reverse primer, 25 μL of 2×Taq PCR MasterMix, 2 μL of DNA template, and double-distilled water. The PCR thermal cycling parameters were set to an initial denaturation at 95 °C for 5 minutes, followed by 30 cycles of 95 °C for 30 seconds, 57 °C for 60 seconds, and 72 °C for 30 seconds, with a concluding extension phase at 72 °C for 10 minutes. The PCR results were validated through 1% agarose gel electrophoresis and subsequently subjected to sequencing via the dideoxy chain-termination approach at Sangon Biotech Co., Ltd. (Shanghai, China) [28]. The sequences of the 16S rRNA genes from the five bacterial strains have been submitted and archived in the GenBank repository, and corresponding accession numbers have been assigned: PQ351188, PQ351189, PQ351190, PQ351191, and PQ351192.

4. In the result sectionIt is mention that percentage increases in plant traits like PT (plant height) and DW (dry weight). Could these increases be contextualized in terms of their biological or agricultural significance? What do these percentages mean for the practical application of PGPR in farming or plant growth?

Answer: Yes, it is possible to contextualize these increases in terms of biological or agricultural significance. An increase in plant height (PT) often means that the plant can better access sunlight. Taller plants can spread their leaves more widely, increasing the surface area available for photosynthesis. This allows the plant to produce more organic matter and energy, which is essential for its growth and development. Dry weight (DW) is a measure of the total amount of organic matter in a plant, excluding water. An increase in dry weight indicates that the plant has accumulated more biomass, which means it has produced and stored more carbohydrates, proteins, and other essential macromolecules. This is a sign of healthy growth and development. The percentage increases in PT and DW can guide farmers in adjusting their fertilization practices. If PGPR application leads to significant increases in these traits, farmers may be able to reduce the use of chemical fertilizers, which not only saves costs but also reduces environmental pollution.

5. Why were the specific temperature ranges (10 °C, 15 °C, 20 °C) chosen? Could the authors explain the relevance of these temperatures to the natural habitat or agricultural practices of Elymus nutans Griseb (EnG)?

Answer: The temperature conditions in the potting experiments in this study were designed based on the temperature gradient during the growing season of *Elymus nutans* Griseb on the Tibetan Plateau. To this we add in materials and methods. In addition, we have added a graph of the annual temperature on the Qinghai - Tibet Plateau to Figure 1, and

the temperature range during the growing season of *Elymus nutans* Griseb (June, July, and August) is circled with a red box.

Furthermore, according to the climatic temperature during the growing season (June, July, and August) of *En*G on the Tibetan Plateau (Fig. 1), this study was set up with temperature gradients of 10 °C, 15 °C, and 20 °C. Incubation was carried out in three incubators with a light (16 h)/dark (8 h) cycle (light at 350 $\mu Em^{-2} s^{-1}$) and 60% humidity. Plant seeds (wild *En*G seeds collected in Nagqhu, Tibet) were sterilized on the

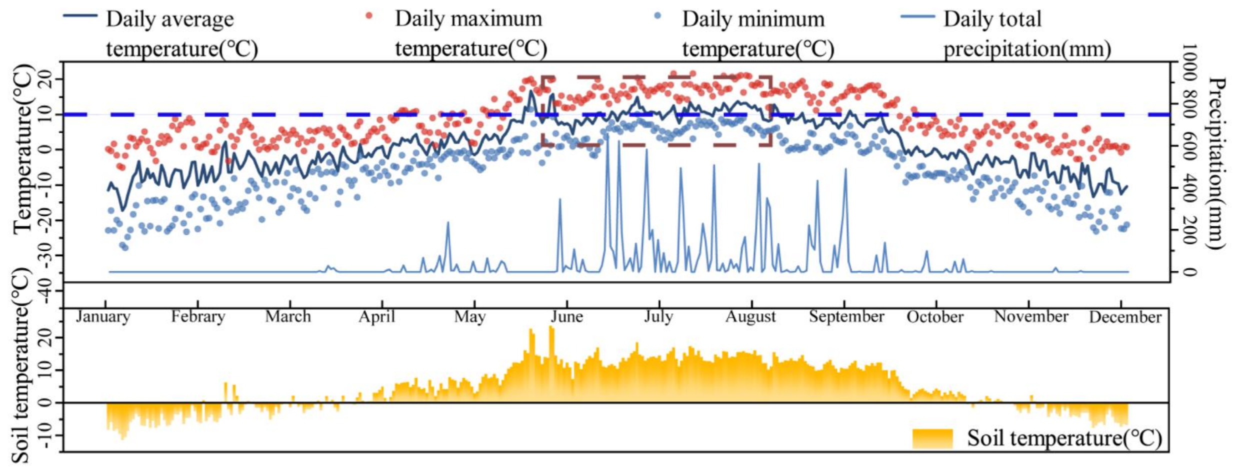

6. There is a significant increase in Chl (chlorophyll content). What is the implication of increased chlorophyll content in terms of plant photosynthetic efficiency or overall health?

Answer: An increase in chlorophyll content is generally a positive sign for plants, contributing to improved photosynthetic efficiency and overall plant health, which are essential for the plant's growth, development, and survival in various environments.

7. The root traits like RSA (root surface area) and RL (root length) are significantly affected by PGPR. Could the authors discuss how these changes in root architecture influence the overall nutrient uptake and drought resistance of EnG?

Answer: The root surface area (RSA) is significantly influenced by PGPR. The increased RSA expands the nutrient absorption area of plants, thereby enhancing their nutrient absorption efficiency. We have also added and discussed this part in the discussion section.

with these findings, the culture substrate containing PGPR elicited IAA production (Table 1). The expansion of the root system surface area augments the nutrient absorption area, which is beneficial for facilitating the uptake of nutrients and water [44]. It is worth noting that there was a significant correlation between RSA levels and PE (Fig.7) ($p<0.01$). This indicates that the PGPR is capable of enhancing the nutrient uptake efficiency of *En*G by promoting root growth [45].

8. The paper mentions that RL was significantly higher at 15 °C than at 20 °C. What could be the physiological reasons

behind this temperature-specific root growth pattern?

Answer: After the addition of mixed bacterial solution to the culture substrate, the root length of *Elymus nutans* Griseb was significantly higher at 15°C than at 20°C. This is because PGPR have different levels of temperature tolerance, and 15°C may be the optimal temperature for this plant. We have also added and discussed this part in the discussion section.

Temperature plays a key role in restricting the distribution and output of plants [60]. Temperature exerts distinct influences on plants [61]. The PGPR have different levels of temperature tolerance, which in turn affects their plant growth-promoting effects and plant growth [62]. The interaction between temperature and the application of PGPR inoculum significantly affected the root length of *En*G ($p<0.001$) (Table 2). The RL of *En*G was significantly higher at 15 °C than at 20 °C ($p<0.05$) (Fig.4a). This could be because 15°C is the optimal temperature for this PGPR. However, under the influence of PGPR, the *En*G [illegible]

9. Why is there a significant increase in SS at 10 °C compared to 20 °C under the T3 treatment? Can the authors provide a rationale for this temperature-dependent response?

Answer: In this study, both the T3 and T1 treatments were added with the mixed bacterial liquid of PGPR, and in both cases, the soluble sugar (SS) content at 10 °C was significantly higher than that at 20 °C. This is because low temperatures may induce changes in the activity of enzymes related to the synthesis of soluble sugars (SS). At 10 °C, these enzymes may be more inclined to break down and convert stored complex carbohydrates, such as starch, into soluble sugars. This is due to the fact that upon sensing the low - temperature environment, plants initiate a series of physiological adaptation mechanisms. They transform less mobile nutrient forms, like starch, into soluble sugars that are more easily transported and utilized, so as to maintain the osmotic balance and energy supply of cells

method to foster growth by enhancing their tolerance to various abiotic stresses [61]. It is worth noting that the content of SS and SP under the T1 and T3 treatment was significantly higher at 10 °C than at 20 °C ($p < 0.05$) (Fig. 5b). This is because low temperatures may induce changes in the activity of enzymes related to the synthesis of soluble sugars (SS). At 10 °C, these enzymes may be more inclined to break down and convert stored complex carbohydrates, such as starch, into soluble sugars. This is due to the fact that upon sensing the low - temperature environment, plants initiate a series of physiological adaptation mechanisms. They transform less mobile nutrient forms, like starch, into soluble sugars that are more easily transported and utilized, so as to maintain the osmotic balance and energy supply of cells [62,63].

10. The increases in NAE (nitrogen agronomic efficiency), PAE (phosphorus agronomic efficiency), and KAE (potassium agronomic efficiency) are significant. Could the authors elaborate on the potential long-term benefits of these increases for sustainable agriculture?

Answer: In this study, the T3 treatment significantly increased the NAE, PAE, and KAE of *En*G at 15°C and 20°C. This indicates that the PGPR is capable of enhancing the nutrient uptake efficiency of *En*G, reducing the dependence on chemical fertilizers, and thereby promoting sustainable agricultural development. We have also added and discussed this part in the discussion section.

Bacillus megaterium and Bacillus mucilaginous inoculants not only boosted plant growth but also enhanced the plants' nutrient absorption capabilities, particularly in terms of total N, P, and K. In this study, the T3 treatment significantly increased the NAE, PAE, and KAE of *En*G at 15°C and 20°C. This indicates that the PGPR is capable of enhancing the nutrient uptake efficiency of *En*G, reducing the dependence on chemical fertilizers, and thereby promoting sustainable agricultural development [48]. In

11. The interaction between temperature and PGPR significantly affects RL. Could the authors discuss the possible mechanisms or environmental factors that might drive this interaction?

Answer: The interaction between temperature and PGPR significantly affects RL. This can be attributed to the combined effects of temperature on root development and the ability of PGPR to modulate plant hormone levels and improve nutrient and water uptake. We have added the possible mechanisms driving this interaction in the discussion.

Temperature plays a key role in restricting the distribution and output of plants [64].It is a critical environmental factor that profoundly influences plant root growth, shaping root system architecture and influencing nutrient uptake and overall plant health [65]. In this study, the interaction between temperature and the application of PGPR inoculum significantly affected the root length of *En*G ($p<0.001$) (Table 2).

This can be attributed to the combined effects of temperature on root development and the ability of PGPR to modulate plant hormone levels and improve nutrient and water uptake[66]. Consequently, the RL of *En*G was significantly higher at 15 °C than at 20 °C ($p<0.05$) (Fig.4a). However, under the influence of

Reviewer #5:

1. Abstract is overly dense and uses unexplained abbreviations (e.g., "SS and SP") which may confuse non-expert readers.

Answer: We are very grateful for the reviewer's suggestions. We have revised the abstract. For soluble sugar (SS) and

soluble protein (SP), we no longer use their abbreviated names in the abstract.

Abstract

In order to show the growth-promoting effects of PGPR (S1, S2, S3, S4 and S5) isolated from the rhizosphere soil of *Elymus nutans* Griseb (*En*G) on the Tibetan Plateau. In this study, we simulated three temperature gradients (10°C, 15°C, and 20°C) during the growing season of *Elymus nutans* Griseb on the Tibetan Plateau, and set up four incubation substrate treatments:T1(addition of PGPR and no addition of NPK fertilizers), T2 (neither PGPR nor NPK fertilizers), T3 (addition of PGPR and NPK fertilizers), and T4 (no addition of PGPR, but addition of NPK fertilizers), to explore the effects of PGPR on the growth and nutrient (NPK) utilization of *Elymus nutans* Griseb at different temperatures. The results showed that compared with T2, the plant height (PT) of T1 increased by 51.72% to 70.67%, the dry weight (DW) increased by 24.99% to 51.25%, the soluble sugar (SS) and soluble protein (SP) content significantly increased by 59.37% and 369.66% at 10 °C respectively ($p<0.05$), and significantly increased by 100.17% and 94.5% at 15 °C respectively ($p<0.05$). Compared with T4, T3 significantly decreased the physiological efficiency of N (NPE) at 15 °C and 20 °C by 40.43% and 72.11%, respectively. In summary, our results showed that this PGPR can promote the growth of *Elymus nutans* Griseb and improve its nutrient utilization efficiency.

2. The broader ecological and economic implications of focusing on Elymus nutans in the introduction are not sufficiently emphasized.

Answer: We have supplemented the broader ecological and economic implications of Elymus nutans in the introduction.

highland pastures of the Qinghai-Tibetan Plateau in China [14]. As an excellent pasture grass in the Qinghai-Tibetan Plateau region in China, it has strong resistance and tolerance to cold, rich nutrients, good palatability, high yield, and tolerance to desert areas among others. It can also prevent winds and fix sands, conserve soil and water, and accelerate the process of ecological restoration of the grassland, which can effectively reduce the economic losses caused by natural disasters [15]. Thus, it is widely planted in the restoration of degraded alpine meadows on the Qinghai - Tibet Plateau [16].

3. Put clear distinction between biological and technical replicates.

Answer: Thank you for your valuable comments. We thoroughly recognize the critical importance of unambiguously

discriminating between biological replicates and technical replicates throughout our research endeavor. Biological replicates, by definition, constitute independent samples that epitomize the inherent biological variability within the target population. In the context of our experimental protocol, we procured five disparate rhizosphere soil samples from diverse geographical loci. Concurrently, technical replicates were instituted with the objective of appraising the precision and reproducibility of our measurement modalities. Specifically, for each individual biological replicate-derived rhizosphere soil sample, we executed a minimum of three replicated measurements. We have already effected the requisite corrections within the "Materials and Methods" section. We are confident that these refinements will augment the clarity and stringency of our research and, optimistically, resolve your concerns.

2.1. Sources of PGPR

The sampling area is located in Nagqhu Town, Seni District, Nagqhu City, Tibet Autonomous Region, with geographic coordinates of 31°26′N latitude and 92°07′E longitude (Fig. 1), and an average elevation of 4450 m. Three sampling sites with good growth and disease-free *En*G were randomly selected, and the rhizosphere soil of the *En*G was collected using root shaking method, with five replicate mixes randomly sampled at each site. And the rhizosphere soil, together with the fine roots, were put into sterile self-sealing bags and stored at low temperature (4°C) to be brought back to the laboratory. The collected soil samples were prepared into 10^{-4} and 10^{-5} soil suspensions and 100 µL of soil suspension was aspirated and spread evenly on beef paste peptone agar plates for bacterial purification and isolation of bacteria.

4. There is a lack of details on how environmental factors like light intensity and humidity were controlled across treatments.

Answer: Thank you for your valuable comments. There is a lack of details on how environmental factors like light intensity and humidity were controlled across treatments. We have made revisions and supplements in the Materials and Methods section.

Furthermore, according to the climatic temperature during the growing season (June, July, and August) of *En*G on the Tibetan Plateau (Fig. 1), this study was set up with temperature gradients of 10 °C, 15 °C, and 20 °C. Incubation was carried out in three incubators with a light (16 h)/dark (8 h) cycle (light at 350 μEm^{-2} s^{-1}) and 60% humidity. Plant seeds (wild *En*G seeds collected in Nagqhu, Tibet) were sterilized on the

5. Statistical methods (e.g., SPSS) are mentioned but without sufficient elaboration on key assumptions (e.g., normality checks).

Answer: In the context of our research, prior to conducting parametric tests on each dataset, we initiated a preliminary assessment using the Shapiro-Wilk test to ascertain the conformity of the data to a normal distribution pattern. This methodological step was integral to ensuring the validity and reliability of our subsequent statistical inferences. Given that parametric tests hinge upon the assumption of normality, a meticulous verification process was imperative. Should the resultant p-value from the Shapiro-Wilk test surpass the 0.05 threshold, we proceeded with the parametric analysis under the presumption of a normally distributed dataset. Conversely, in instances where the p-value fell below this critical value, we explored alternative non-parametric methodologies or implemented data transformation techniques to approximate normality, followed by a re-evaluation of the distributional characteristics. This comprehensive approach safeguarded the robustness of our statistical undertakings and enhanced the overall integrity of our research findings. We have made revisions and supplements in the Materials and Methods section.

2.7 Statistical analyses

Statistical analyses were performed using SPSS software (version 27.0; SPSS Inc., Chicago, IL, USA). In the context of our research, prior to conducting parametric tests on each dataset, we initiated a preliminary assessment using the Shapiro-Wilk test to ascertain the conformity of the data to a normal distribution pattern. Mean differences were determined using the least significant difference method and the Tukey test at a significance level of 5%. Statistical significance is reported at $p < 0.05$. All visualizations were produced using R 4.4.1. The relationship between the growth indicators of *En*G and nutrient utilization was analyzed through Mantel analysis.

6. Limited explanation for unexpected trends, such as the decrease in physiological efficiency of nitrogen (NPE) at higher temperatures in T3 treatment.

Answer: A decrease in the nitrogen physiological efficiency (NPE) under higher temperatures was observed in the T3 treatment. This may be due to the fact that PGPR transform unavailable nutrient forms for plants into available forms, which reduces the plants' need for fertilizer. We have made supplements and elaborations in the discussion section.

Compared with treatment T4, T3 significantly decreased the NPE of *EnG* at 20 °C by 72.11% (p<0.001) (Fig. 6a). This may be due to the fact that PGPR transform unavailable nutrient forms for plants into available forms [52], which reduces the plants' need for fertilizer. PGPR plays a significant role in agriculture by facilitating the circulation of plant nutrients and reducing the reliance on chemical fertilizers

7. You mentioned p-values but could not discuss the biological significance of findings.

Answer: Thank you for pointing out this important aspect. We acknowledge that while we presented the p - values in our study, the discussion of the biological significance of the findings was insufficient, and we sincerely apologize for this oversight. For the statistically significant results indicated by the p-values, we delved into the underlying biological mechanisms. For example, if we found a significant difference in a certain physiological parameter between two experimental groups, we explored how this difference might be related to known biological pathways. We referred to existing literature to explain how the observed changes could affect physiological indicators, which in turn could influence nutrient uptake. A significant change in nutrient uptake efficiency might have an impact on plant growth.

8. Table 2 presents complex statistical results without adequate textual interpretation for non-specialists.

Answer: I deeply apologize that Table 2 showcases intricate statistical outcomes without furnishing sufficient textual elucidations for non - experts. Your constructive feedback is highly appreciated. We've implemented revisions at the relevant locations, and the modified sections are presented as follows.

Table 2 The main effects of temperature (T), PGPR, and fertilizers (NPK) on *Elymus nutans Griseb*. The values in the table represent the statistical significance (p - values) of the effects of different sources of variation on each trait. PT: plant height; DW: dry weight, Chl: plant chlorophyll; RL: root length; RB: number of root branches; RSA: root surface area; RD: root average diameter; SS: soluble sugars; SP: soluble proteins.

Source of variation	PT	DW	Chl	RL	RB	RSA	RD	SS	SP
**T**	<0.001	<0.001	<0.001	<0.001	0.076	<0.001	<0.001	<0.001	<0.001
**NPK**	<0.001	<0.001	<0.001	<0.001	<0.001	<0.001	0.847	<0.001	<0.001
**PGPR**	<0.001	<0.001	<0.001	<0.001	<0.001	0.007	<0.001	<0.001	<0.001
**T* NPK**	0.333	0.007	<0.001	0.429	0.893	0.012	0.015	<0.001	<0.001
**T* PGPR**	0.629	0.028	<0.001	<0.001	<0.001	0.392	<0.001	<0.001	<0.001
**NPK* PGPR**	0.386	0.007	<0.001	0.174	0.949	0.728	<0.001	<0.001	<0.001
**T* NPK* PGPR**	0.290	0.026	<0.001	0.020	0.898	0.010	0.181	<0.001	<0.001

Note： When $p < 0.05$, it shows significant impact. When $p < 0.01$, it signifies an extremely remarkable influence.

9. For figures, legends are overly technical and could be simplified for clarity. The color coding in heatmaps (Figure 7a) is inconsistent, making interpretation less intuitive. Also, text is not legible. In Fig. 2, measuring scales are not legible. Better to use a common scale for all and that should be clearly visible.

Answer: Many thanks for your invaluable insights. First and foremost, I would like to offer my sincere apologies for the details and imperfections of the images in this manuscript. Concerning the overly technical nature of the image legends, we have made every effort to streamline them. In Figure 7, the inconsistent color - coding is deliberately designed to differentiate between the correlation in the heat - map section and the nutrient utilization status.

Moreover, in response to issues such as unclear fonts in the images, we have implemented measures to enhance the image quality. Finally, we deeply regret the problem of the measurement scale in Figure 2. This figure is composed of multiple sub - images. During the shooting process, due to a variety of factors, we were unable to preserve images with ultra - high standards and quality. Although the images we employed may not have optimal clarity, we can assure you with full

confidence that the data are authentic and have been rigorously verified.

10. Improve discussion: there are repetitive points about the benefits of PGPR, leading to redundancy. Alternative mechanisms for PGPR effects (e.g., microbial community shifts) are not discussed.

Answer: We refined the discussion and explored alternative mechanisms for the effects of PGPR. In light of the issues concerning language redundancy within the discussion, we have effected amendments in accordance with the requisites put forward by the reviewers. Considering that the ensuing phase of this research endeavor remains unfinished, there is still ample scope for exploration with regard to the microbial assemblage, structural configuration as well as functional metabolism both in the substrate and plants subsequent to strain inoculation.

11.Limited exploration of variability among the five PGPR strains tested. Discuss limitations of the study in the conclusion, such as the exclusion of field trials or the potential for long-term ecological impacts. Give practical recommendations for scaling PGPR applications are underdeveloped.

Answer: We have modified the discussion part, and for those regions where the application of PGPR is not well-developed, we have offered practical and feasible suggestions. Additionally, we have supplemented with some referenced papers.

Data Review URL

Click here to download Data Review URL
http://cloudcenter.tianditu.gov.cn/dataSource
