# Supplementary material for: Effect of PGPR on growth and nutrient utilization of Elymus nutans Griseb at different temperatures
Source: PLoS One. 2025 May 12;20(5):e0323613. doi: 10.1371/journal.pone.0323613 (PMC12068589; doi:10.1371/journal.pone.0323613)
Supplement: Attachment — Submitted filename: Response to Reviewers - 2025-02-24T101919.976.docx [file pone.0323613.s005.docx]

**Response to Reviewers**

Manuscript Number: **PONE-D-24-56644**

Mechanisms of growth promotion and resistance of PGPR to *Elymus nutans* Griseb at different temperatures

Dear Editor and Reviewers,

Thank you very much for your comments and professional advice. These opinions help to improve academic rigor of our article. Based on your suggestion and request we have made corrected modifications on the revised manuscript. We hope that our work can be improved again. We would like to show the details as follows:

**Journal Requirements:**

Via meticulous inspection and comprehensive review, we have ascertained that our manuscript adheres to the stylistic requisites of PLOS ONE, encompassing the stipulations for file naming. We have successfully applied for an ORCID iD and have accomplished its verification within the Editorial Manager. In relation to the matter of our work permit, it should be noted that all our undertakings, spanning experimental procedures and the writing process, were carried out within the confines of the laboratory affiliated with our primary institution, thereby obviating the need for any additional permits. What distinguishes our work is the fact that the funder assumes the role of a co-collaborator in this study. The funder actively participated in the study design, data gathering and analytical processes, as well as in making the decision regarding publication and manuscript compilation. Finally, with respect to the concern surrounding Figure 1, the geographical information map, we hereby affirm that we have refrained from utilizing any copyrighted graphic or textual materials published by third parties. We confirm that all images included in our submission can be published under the CC BY 4.0 license as required by PLOS ONE. The base maps of our geographical information were exclusively derived from publicly accessible information disclosed on the website: <https://www.tianditu.gov.cn/>, the specific URL of the web page for retrieving this data is: <https://cloudcenter.tianditu.gov.cn/dataSource>. The map, base map, shape files, and map data in Figure 1 were obtained from the National Geographic Information Public Service Platform "TianDiTu"( <https://cloudcenter.tianditu.gov.cn/administrativeDivision/>). The data was collected in 2024, the map base layer has been reviewed and approved with the approval number GS (2024) 0650. The TianDiTu website offers a variety of map services and data resources. The base map data downloaded on this platform is in GeoJSON format. We promise that the base map has not been modified. We accessed and utilized the data in strict accordance with the platform's regulations (<https://www.tianditu.gov.cn/about/copyright>). The software used to create these maps is ArcGIS 10.8. If you need the source files of the map base layers we used, we will also upload them to your journal’s system in the form of attachments. Additionally, we collected rhizosphere soil from the high-altitude pastoral areas where Elymus nutans is well adapted for strain isolation and identification, and we also studied the plant-growth-promoting properties of the strains. The general information about the sampling site (geographical coordinates, vegetation type, and climatic characteristics) has been described in detail in the Materials and Methods section. If our modification of the location map does not meet your requirements and the journal's standards, we can omit the location map and provide an alternative figure that only retains the meteorological map. We truly appreciate your invaluable comments and extend our gratitude for your diligent efforts.

**Figure 1:**

**Alternative for Figure 1:**

**Reviewer #2**:

1.This is a valuable study as strains were obtained and characterized from the Tibetan Plateau, a unique and understudied ecosystem. The potential use of plant growth promoting bacteria in pasture production in this ecosystem is an interesting study area. However, it seems to me that the tested conditions (fertilizer scheme, substrate and temperature conditions) are not relevant to the application area, the Tibetan Plateau. Therefore, the results of this study are far less relevant and do not support the stated conclusions.

Answer: Thank you for the reviewer's comments. The fertilizer method of this study refers to the “3414” design, which determines the optimum dosage and ratio of N-P-K fertilizers at the “2” level of N, P and K fertilizers. Its purpose is to explore the impact of PGPR on the nutrient utilization of *Elymus nutans* Griseb. The substrate conditions for this study were formulated with reference to the soil characteristics of the sampling sites. The temperature conditions in the potting experiments in this study were designed based on the temperature gradient during the growing season of *Elymus nutans* Griseb on the Tibetan Plateau. To this we add in materials and methods. In addition, we have added a graph of the annual temperature on the Qinghai - Tibet Plateau to Figure 1, and the temperature range during the growing season of *Elymus nutans* Griseb (June, July, and August) is circled with a red box.

2. The results and discussion are very difficult to understand due to the use of many abbreviations, coded treatments and a lack of structure in the text. Therefore, I was also not able to verify the statistical analyses.

Answer: First of all, I would like to apologize for the use of abbreviations of words and the obscurity of the structure in the article. Perhaps this kind of writing may have a negative impact on the reading experience. During the writing of the article, as some professional terms are rather long, we noted the abbreviations after their full names when they first appeared. These abbreviations were also adopted in accordance with those used in currently published articles. Subsequently, we used these abbreviations throughout the rest of the article. We have also conducted checks on the language logic and structure. We sincerely apologize for any inconvenience caused to you. In addition, regarding the issue of statistical analysis, we have compiled and uploaded the original data for your reference. You can access it in Figshare (https://doi.org/10.6084/m9.figshare.27094078.v1). Thank you again for your valuable comments, and we hope you can forgive any inconvenience.

3. Important information is lacking in Material and methods, e.g. NFM-agar, brief descriptions of methods, the preparation of inoculum (CFU counts etc).

Answer: We supplemented the materials and methods and explained the NFM-agar as well as the concentration of bacterial agent (1×10^8^CFU/ml).

4. The manuscript does not disclose which five strains out of 273 strains were selected, and based on which criteria these were selected.

Answer: We supplemented materials regarding the origin of the 273 strains and screened for excellent strains by measuring their growth - promoting abilities. Through the determination of growth - promoting capabilities, we separately selected five strains with the highest nitrogen - fixing, phosphate - solubilizing, potassium - releasing, and IAA - secreting abilities for identification. The magnitudes of these abilities are shown in Table 1.

Reviewer #3:

1. There is no experiment of mechanism of growth promotion and resistance of PGPR (eg. ROS, PO, PPO, PAL activity; amount of MDA, GSH, IAA, antioxidants, etc.) in this study. Here, title of this paper does not represent the results. This research is mainly focused on nutrient uptake efficiency of PGPR in peat soil based substrate with different temperature. In another words, authors should mention what and where is the PGPR mechanism in this study?

Answer: I deleted the word “mechanism” in the title, and changed the title of the manuscript to“Effect of PGPR on growth and cold tolerance of *Elymus nutans* Griseb at different temperatures”.

Reviewer #4:

1. The abstract should be rewritten in a more systematic pattern to improve clarity and readability. Clarify the research aim, emphasizing the focus on temperature effects on PGPR-mediated plant growth and nutrient utilization. Specify the type of pot experiments conducted, including the temperature ranges used. Explain the significance of the treatments (T1, T2, T3, T4) briefly and consistently. Summarize the findings succinctly, focusing on the most significant results.

Answer: Thank you for your valuable comments. We have rewritten the abstract according to your suggestion. The purpose of the study was made clearer and the focus on the effect of temperature on PGPR-mediated plant growth and nutrient utilization was emphasized. The indoor pot experiment, the four treatments, and the temperature gradient settings are also clarified.

2. The introduction section needs to incorporate more recent literature to provide a comprehensive background. In introduction section ([12] revealed that specific PGPR strains can impart cold resistance to rice plants, boosting their viability, fertility, and yield related traits and accelerating the progression of their reproductive cycle. Furthermore, variation in temperature tolerance exists among PGPR strains, affecting efficacy and plant health [13].), needs to rewrite.

Answer: We have revised the introductory section and incorporated the most recent literature.

3. In methodology section the sub-heading “Identification of PGPR strains” is written well but its too lengthy, make it simple and short.

Answer: We have modified and adapted the methodology sections as appropriate and have simplified the identification of PGPR strains section as appropriate.

4. In the result sectionIt is mention that percentage increases in plant traits like PT (plant height) and DW (dry weight). Could these increases be contextualized in terms of their biological or agricultural significance? What do these percentages mean for the practical application of PGPR in farming or plant growth?

Answer: Yes, it is possible to contextualize these increases in terms of biological or agricultural significance. An increase in plant height (PT) often means that the plant can better access sunlight. Taller plants can spread their leaves more widely, increasing the surface area available for photosynthesis. This allows the plant to produce more organic matter and energy, which is essential for its growth and development. Dry weight (DW) is a measure of the total amount of organic matter in a plant, excluding water. An increase in dry weight indicates that the plant has accumulated more biomass, which means it has produced and stored more carbohydrates, proteins, and other essential macromolecules. This is a sign of healthy growth and development. The percentage increases in PT and DW can guide farmers in adjusting their fertilization practices. If PGPR application leads to significant increases in these traits, farmers may be able to reduce the use of chemical fertilizers, which not only saves costs but also reduces environmental pollution.

5. Why were the specific temperature ranges (10 °C, 15 °C, 20 °C) chosen? Could the authors explain the relevance of these temperatures to the natural habitat or agricultural practices of Elymus nutans Griseb (EnG)?

Answer: The temperature conditions in the potting experiments in this study were designed based on the temperature gradient during the growing season of *Elymus nutans* Griseb on the Tibetan Plateau. To this we add in materials and methods. In addition, we have added a graph of the annual temperature on the Qinghai - Tibet Plateau to Figure 1, and the temperature range during the growing season of *Elymus nutans* Griseb (June, July, and August) is circled with a red box.

6. There is a significant increase in Chl (chlorophyll content). What is the implication of increased chlorophyll content in terms of plant photosynthetic efficiency or overall health?

Answer: An increase in chlorophyll content is generally a positive sign for plants, contributing to improved photosynthetic efficiency and overall plant health, which are essential for the plant's growth, development, and survival in various environments.

7. The root traits like RSA (root surface area) and RL (root length) are significantly affected by PGPR. Could the authors discuss how these changes in root architecture influence the overall nutrient uptake and drought resistance of EnG?

Answer: The root surface area (RSA) is significantly influenced by PGPR. The increased RSA expands the nutrient absorption area of plants, thereby enhancing their nutrient absorption efficiency. We have also added and discussed this part in the discussion section.

8. The paper mentions that RL was significantly higher at 15 °C than at 20 °C. What could be the physiological reasons behind this temperature-specific root growth pattern?

Answer: After the addition of mixed bacterial solution to the culture substrate, the root length of *Elymus nutans* Griseb was significantly higher at 15°C than at 20°C. This is because PGPR have different levels of temperature tolerance, and 15°C may be the optimal temperature for this plant. We have also added and discussed this part in the discussion section.

9. Why is there a significant increase in SS at 10 °C compared to 20 °C under the T3 treatment? Can the authors provide a rationale for this temperature-dependent response?

Answer: In this study, both the T3 and T1 treatments were added with the mixed bacterial liquid of PGPR, and in both cases, the soluble sugar (SS) content at 10 °C was significantly higher than that at 20 °C. This is because low temperatures may induce changes in the activity of enzymes related to the synthesis of soluble sugars (SS). At 10 °C, these enzymes may be more inclined to break down and convert stored complex carbohydrates, such as starch, into soluble sugars. This is due to the fact that upon sensing the low - temperature environment, plants initiate a series of physiological adaptation mechanisms. They transform less mobile nutrient forms, like starch, into soluble sugars that are more easily transported and utilized, so as to maintain the osmotic balance and energy supply of cells

10. The increases in NAE (nitrogen agronomic efficiency), PAE (phosphorus agronomic efficiency), and KAE (potassium agronomic efficiency) are significant. Could the authors elaborate on the potential long-term benefits of these increases for sustainable agriculture?

Answer: In this study, the T3 treatment significantly increased the NAE, PAE, and KAE of *En*G at 15°C and 20°C. This indicates that the PGPR is capable of enhancing the nutrient uptake efficiency of *En*G, reducing the dependence on chemical fertilizers, and thereby promoting sustainable agricultural development. We have also added and discussed this part in the discussion section.

11. The interaction between temperature and PGPR significantly affects RL. Could the authors discuss the possible mechanisms or environmental factors that might drive this interaction?

Answer: The interaction between temperature and PGPR significantly affects RL. This can be attributed to the combined effects of temperature on root development and the ability of PGPR to modulate plant hormone levels and improve nutrient and water uptake. We have added the possible mechanisms driving this interaction in the discussion.

**Reviewer #5**:

1. Abstract is overly dense and uses unexplained abbreviations (e.g., "SS and SP") which may confuse non-expert readers.

Answer: We are very grateful for the reviewer's suggestions. We have revised the abstract. For soluble sugar (SS) and soluble protein (SP), we no longer use their abbreviated names in the abstract.

2. The broader ecological and economic implications of focusing on Elymus nutans in the introduction are not sufficiently emphasized.

Answer: We have supplemented the broader ecological and economic implications of Elymus nutans in the introduction.

3. Put clear distinction between biological and technical replicates.

Answer: Thank you for your valuable comments. We thoroughly recognize the critical importance of unambiguously discriminating between biological replicates and technical replicates throughout our research endeavor. Biological replicates, by definition, constitute independent samples that epitomize the inherent biological variability within the target population. In the context of our experimental protocol, we procured five disparate rhizosphere soil samples from diverse geographical loci. Concurrently, technical replicates were instituted with the objective of appraising the precision and reproducibility of our measurement modalities. Specifically, for each individual biological replicate-derived rhizosphere soil sample, we executed a minimum of three replicated measurements. We have already effected the requisite corrections within the “Materials and Methods” section. We are confident that these refinements will augment the clarity and stringency of our research and, optimistically, resolve your concerns.

4. There is a lack of details on how environmental factors like light intensity and humidity were controlled across treatments.

Answer: Thank you for your valuable comments. There is a lack of details on how environmental factors like light intensity and humidity were controlled across treatments. We have made revisions and supplements in the Materials and Methods section.

5. Statistical methods (e.g., SPSS) are mentioned but without sufficient elaboration on key assumptions (e.g., normality checks).

Answer: In the context of our research, prior to conducting parametric tests on each dataset, we initiated a preliminary assessment using the Shapiro-Wilk test to ascertain the conformity of the data to a normal distribution pattern. This methodological step was integral to ensuring the validity and reliability of our subsequent statistical inferences. Given that parametric tests hinge upon the assumption of normality, a meticulous verification process was imperative. Should the resultant p-value from the Shapiro-Wilk test surpass the 0.05 threshold, we proceeded with the parametric analysis under the presumption of a normally distributed dataset. Conversely, in instances where the p-value fell below this critical value, we explored alternative non-parametric methodologies or implemented data transformation techniques to approximate normality, followed by a re-evaluation of the distributional characteristics. This comprehensive approach safeguarded the robustness of our statistical undertakings and enhanced the overall integrity of our research findings. We have made revisions and supplements in the Materials and Methods section.

6. Limited explanation for unexpected trends, such as the decrease in physiological efficiency of nitrogen (NPE) at higher temperatures in T3 treatment.

Answer: A decrease in the nitrogen physiological efficiency (NPE) under higher temperatures was observed in the T3 treatment. This may be due to the fact that PGPR transform unavailable nutrient forms for plants into available forms, which reduces the plants’ need for fertilizer. We have made supplements and elaborations in the discussion section.

7. You mentioned p-values but could not discuss the biological significance of findings.

Answer: Thank you for pointing out this important aspect. We acknowledge that while we presented the p - values in our study, the discussion of the biological significance of the findings was insufficient, and we sincerely apologize for this oversight. For the statistically significant results indicated by the p-values, we delved into the underlying biological mechanisms. For example, if we found a significant difference in a certain physiological parameter between two experimental groups, we explored how this difference might be related to known biological pathways. We referred to existing literature to explain how the observed changes could affect physiological indicators, which in turn could influence nutrient uptake. A significant change in nutrient uptake efficiency might have an impact on plant growth.

8. Table 2 presents complex statistical results without adequate textual interpretation for non-specialists.

Answer: I deeply apologize that Table 2 showcases intricate statistical outcomes without furnishing sufficient textual elucidations for non - experts. Your constructive feedback is highly appreciated. We've implemented revisions at the relevant locations, and the modified sections are presented as follows.

9. For figures, legends are overly technical and could be simplified for clarity. The color coding in heatmaps (Figure 7a) is inconsistent, making interpretation less intuitive. Also, text is not legible. In Fig. 2, measuring scales are not legible. Better to use a common scale for all and that should be clearly visible.

Answer: Many thanks for your invaluable insights. First and foremost, I would like to offer my sincere apologies for the details and imperfections of the images in this manuscript. Concerning the overly technical nature of the image legends, we have made every effort to streamline them. In Figure 7, the inconsistent color - coding is deliberately designed to differentiate between the correlation in the heat - map section and the nutrient utilization status.

Moreover, in response to issues such as unclear fonts in the images, we have implemented measures to enhance the image quality. Finally, we deeply regret the problem of the measurement scale in Figure 2. This figure is composed of multiple sub - images. During the shooting process, due to a variety of factors, we were unable to preserve images with ultra - high standards and quality. Although the images we employed may not have optimal clarity, we can assure you with full confidence that the data are authentic and have been rigorously verified.

10. Improve discussion: there are repetitive points about the benefits of PGPR, leading to redundancy. Alternative mechanisms for PGPR effects (e.g., microbial community shifts) are not discussed.

Answer: We refined the discussion and explored alternative mechanisms for the effects of PGPR. In light of the issues concerning language redundancy within the discussion, we have effected amendments in accordance with the requisites put forward by the reviewers. Considering that the ensuing phase of this research endeavor remains unfinished, there is still ample scope for exploration with regard to the microbial assemblage, structural configuration as well as functional metabolism both in the substrate and plants subsequent to strain inoculation.

11.Limited exploration of variability among the five PGPR strains tested. Discuss limitations of the study in the conclusion, such as the exclusion of field trials or the potential for long-term ecological impacts. Give practical recommendations for scaling PGPR applications are underdeveloped.

Answer: We have modified the discussion part, and for those regions where the application of PGPR is not well-developed, we have offered practical and feasible suggestions. Additionally, we have supplemented with some referenced papers.
