# Supplementary material for: Effect of PGPR on growth and nutrient utilization of Elymus nutans Griseb at different temperatures
Source: PLoS One. 2025 May 12;20(5):e0323613. doi: 10.1371/journal.pone.0323613 (PMC12068589; doi:10.1371/journal.pone.0323613)
Supplement: Attachment — Submitted filename: Comments on PLOS.docx [file pone.0323613.s004.docx]

Comments on “ **Effect of PGPR on growth and nutrient utilization of Elymus nutans Griseb at different temperatures”**

1. It is essential to add phytogenic tree of those 5 strains of PGPR for finding the geographical variation, if any.

2. In Fig 3c : the amounts of ‘Chl’ in both T1 and T3 at 10 &15^0^C were obtained almost same. Again, Phosphorus is a key element that affects chlorophyll levels in plants. Here, it was not discussed- why ‘Chl’ amounts were obtained similar rather NPK was used in T3 ?

3. It is also needed to put the Siderophores test results that represent iron (Fe) accumulation. Iron is involved in the manufacturing process of Chlorophyll (Chl); helps plants growth and development via Photosynthesis and in many metabolic processes.

4. The shelf life and population of PGPR was not evaluated in that substrate. To validate this finding and farmers use, such data is needed.

The Reviewer.
