# Supplementary material for: Effect of PGPR on growth and nutrient utilization of Elymus nutans Griseb at different temperatures
Source: PLoS One. 2025 May 12;20(5):e0323613. doi: 10.1371/journal.pone.0323613 (PMC12068589; doi:10.1371/journal.pone.0323613)
Supplement: Attachment — Submitted filename: Response to Reviewers.docx [file pone.0323613.s007.docx]

**Response to Reviewers**

Manuscript Number: **PONE-D-24-56644**

Effect of PGPR on growth and nutrient utilization of *Elymus nutans* Griseb at different temperatures

Dear Editor and Reviewers,

Thank you very much for your comments and professional advice. These opinions help to improve academic rigor of our article. Based on your suggestion and request we have made corrected modifications on the revised manuscript. We hope that our work can be improved again. We would like to show the details as follows:

**Reviewer #2**:

1. Thank you for revising your manuscript thoroughly. Some comments remain, most importantly a comment raised by another reviewer concerning biological and technical replicates: In response to this comment, you have made adjustments in section 2.1, regarding the isolation of bacterial strains. However, it is in the experimental part of the manuscript i.e. in the incubation experiment (2.5) that biological and technical replicates are needed. Can you provide more detail here, i.e. how many replicates per condition, how many seeds per pot, how many measurements per parameter, did you repeat it at different timepoints, with freshly made microbial mixtures? Secondly, the plants were incubated in three incubators, were the conditions randomized between these incubators?

Answer: We are extremely grateful for the pertinent suggestions from the reviewers. In the incubation experiment (2.5), we carried out both biological and technical replications. For different treatments, we set up 3 replicates. Each pot contained 35 seeds, and each parameter was measured three times for each treatment. (Figure 2). Regarding the use of the mixed bacterial solution, we added it under the same time condition to minimize errors. Secondly, the plants were incubated in three incubators under the conditions of a light (16 h)/dark (8 h) cycle (with a light intensity of 350 μEm⁻² s⁻¹) and 60% humidity. Of course, the configuration and power of the incubators are also the same, and the three incubators are mainly used for three temperature culture experiments. The incubators have automatic temperature, humidity and light control devices, which can precisely control the temperature, humidity and light. In order to ensure that the plants receive the same light in the incubator during the cultivation process, as well as the same temperature and humidity conditions, the position of the samples in the incubator is randomly changed again every 2 days.

2. The study started at an earlier stage then the demonstration of growth promoting effects. I suggest including more context: i.e. rhizosphere bacteria were isolated (at this stage it is still unknown if these are PGPR), selected based on known PGPR traits which were assessed in vitro, and finally tested in plants. This context underlines the novelty of the research: novel PGPR were selected specifically for Eng on Tibetan plateau.

Answer: Thank you very much for the valuable comments provided by the reviewers. We have added more background information in the abstract part: rhizosphere bacteria were isolated and selected based on the known traits of PGPR. These traits were evaluated in vitro and finally tested in plants. Moreover, this background highlights that the PGPR of this study were specifically selected for *En*G in the Qinghai-Tibet Plateau.

3.  2.6.3: briefly explain the methods used to determine these plant nutrient use efficiency, instead of including a reference.

Answer: Thank you for the reviewers' comments. We have briefly described the methods used to determine the nutrient use efficiency of these plants in Section 2.6.3.

4. Change “excellent” strains to e.g. “selected” strains, to remain objective.

Answer: Thank you for your suggestion. We have already changed the description of "excellent" strains to "selected" strains in the article.

5. Table 1: change in caption > 5 strains. Please include identification of the strains, preferably also in abstract and discussion. Are these species known for their PGPR effects?

Answer: Thank you very much for the reviewers' suggestions. We have already included the identification results of the strains in Table 1. Meanwhile, we have supplemented the background conditions for the isolation, screening and identification of the strains in the abstract.

6. readability of the results and discussion remains difficult. Below a few recommendations to improve:
 describe the different treatments instead of uniquely referring to T1, T2 etc.

Answer: Thank you for your suggestion. We have modified the descriptions regarding the treatment methods in the Results and Discussion section, instead of referring to T1, T2, etc. individually.

7. guide the reader to better understand figure 7; i.e. how was this figure made, what does it show, and how should it be interpreted? explain the differences between physiological efficiency (PE) and apparent recovery efficiency (RE) and how this is relevant for this research.

Answer: Thank you for the insightful comments. Below is the elaboration:

Figure 7 visually integrates Mantel’s test and Pearson’s correlation analysis to illustrate relationships between plant physiological parameters and nutrient use efficiency indices. Appropriate additions and supplements have been made for the better understanding of the reader.

The physiological efficiency (PE) of N (NPE), P (PPE), and K (KPE) was calculated as the kg plant yield per kg N, P, and K uptake. The agronomic efficiencies (AE) of N (NAE), P (PAE), and K (KAE) were calculated as the yield increase per kg N, P, and K applied. The apparent recovery efficiency (RE) of N (NRE), P (PRE), and K (KRE) was calculated as the plant N, P, and K uptake (kg ha^-1^) per kg of N, P, and K applied. PE: Highlights PGPR’s role in optimizing nutrient assimilation under temperature stress (e.g., enhanced Chl synthesis via Fe mobilization). RE: Demonstrates PGPR’s ability to reduce fertilizer dependency by improving nutrient recovery (e.g., T3 vs. T4 treatments in Table 1). PE and RE collectively reveal PGPR’s dual impact on nutrient use strategies: PE reflects metabolic optimization, while RE quantifies practical fertilizer savings. For the better understanding of the readers, we have also added and supplemented the corresponding concepts and references in the methods section as appropriate.

8. Discussion: This phrase is misleading and should be nuanced: “Consistent with these findings, the culture substrate containing PGPR elicited IAA production (Table 1)” > The PGPR produced IAA on certain media, but PGPR eliciting IAA production in plants was not studied. Secondly, it is not sure that IAA was also produced in the conditions of the incubation experiment.

Answer: We are very grateful to you, the reviewer, for your valuable comments. We have made revisions to this discussion section.

**Reviewer #3**:

1. It is essential to add phytogenic tree of those 5 strains of PGPR for finding the geographical variation, if any.

Answer: Thank you for the reviewers' comments. I am very sorry that we did not conduct a study of phytogenic tree of those 5 strains of PGPR for finding the geographical variation. In this study, the sampling area is located in Nagqu Town, Seni District, Nagqu City, Tibet Autonomous Region, with the geographical coordinates of 31°26′N latitude and 92°07′E longitude, and an average elevation of 4,450 meters. Three sampling sites with well-grown and disease-free *Elymus nutans* Griseb (*En*G) were randomly selected. The rhizosphere soil of *Elymus nutans* Griseb was collected by the root shaking method, and five mixed replicate samples were randomly collected at each sampling site (Figure 1). In conclusion, the strains were collected from natural grasslands on the northern Tibetan Plateau with little geographic differentiation.

2. In Fig 3c: the amounts of ‘Chl’ in both T1 and T3 at 10 &15^0^C were obtained almost same. Again, Phosphorus is a key element that affects chlorophyll levels in plants. Here, it was not discussed- why ‘Chl’ amounts were obtained similar rather NPK was used in T3?

Answer: Thank you for the reviewers' comments. In this study, the amounts of “Chl” in T1 and T3 were almost the same at 10 °C and 15 °C. The possible reason is that the PGPR bacterial liquid was added in both T1 and T3 treatments. PGPR can promote the growth and development of plant roots by secreting plant hormones such as IAA, thus improving the nutrient absorption efficiency of plants. In the T1 treatment, although nitrogen, phosphorus and potassium fertilizers were not added, PGPR might decompose the insoluble nutrients in the culture medium through its growth-promoting characteristics, enabling plants to utilize the nutrients in the culture medium, including phosphorus, more effectively and thus maintain a relatively high chlorophyll content. In response to this, we have also added the relevant discussions of this part in the article.

3. It is also needed to put the Siderophores test results that represent iron (Fe) accumulation. Iron is involved in the manufacturing process of Chlorophyll (Chl); helps plants growth and development via Photosynthesis and in many metabolic processes.

Answer: We extend our heartfelt gratitude for your meticulous review of our article and the insightful suggestions you have offered. Concerning your recommendation to include the test results of siderophores associated with iron (Fe) accumulation, we have given this proposal serious consideration and are now providing the following clarifications.

Our study aimed to investigate the growth-promoting and resistance mechanisms exhibited by plant-growth-promoting rhizobacteria (PGPR) in *Elymus nutans* Griseb under various temperatures. Our primary focus was on the impact of PGPR on the growth and nutrient uptake efficiency of *Elymus nutans* Griseb, as well as its responsiveness to differing temperature conditions. While iron is vital for plant development, particularly in the synthesis of chlorophyll, photosynthesis, and metabolic processes, it was not the primary focus of our investigation.

We acknowledge the significance of iron accumulation and the pivotal role of siderophores in the interactions between plants and microorganisms. We further recognize the considerable potential for further exploration in this research area. Through active brainstorming, we are currently focusing on the relationship between iron (Fe) and photosynthesis in our ongoing field application experiments of mycorrhizal fertilizers, with the aim of obtaining valuable data. We intend to conduct more in-depth research in future studies, focusing specifically on the relationship between iron and chlorophyll, as well as the influence of PGPR on the iron uptake and utilization by plants. This comprehensive investigation will not only enhance our understanding of the growth-promoting mechanisms of PGPR but also contribute valuable data to the field.

We reiterate our appreciation for your valuable feedback. We trust that you appreciate our current circumstances. We are committed to actively pursuing this crucial topic in our future research endeavors and look forward to contributing more significant findings to the advancement of this field.

4. The shelf life and population of PGPR was not evaluated in that substrate. To validate this finding and farmers use, such data is needed.

Answer: First of all, thank you very much for your valuable suggestion, it is indeed very important to supplement the data on shelf life and colony counts of PGPR in substrates to validate the research findings and practical applications. Of course, we still apologize to you that we neglected to record the shelf life and colony number cycle of PGPR due to the time cycle and subsequent development and utilization of the bacterial fertilizer, because we have been carrying out the improvement and configuration of the bacterial fertilizer, so our strains have been in the state of regular activation. However, in order to ensure the rigor and scientificity of this potting experiment, we took out the samples kept in -80℃ for determining the microbial metabolism of the substrate and verified the activation, and the specific results are attached in the following figure, because considering the structure of the overall article, we hope that we can be allowed to use this result only for answering your questions, after all, it is the shelf-life of the PGPR which has not been systematically and completely recorded. By our activation and counting assay, based on the substrates at the end of the potting experiment (substrates kept in an ultra-low temperature refrigerator), the number of colonies in the substrates with added bacterial solution was significantly higher than in the control. Thank you for your valuable comments, and of course we hope to document its activity and shelf-life in bacterial fertilizers in a complete manner during the subsequent development of the bacterial fertilizer.
