# Supplementary material for: Effect of PGPR on growth and nutrient utilization of Elymus nutans Griseb at different temperatures
Source: PLoS One. 2025 May 12;20(5):e0323613. doi: 10.1371/journal.pone.0323613 (PMC12068589; doi:10.1371/journal.pone.0323613)
Supplement: Attachment — Submitted filename: Comments on PLOS.docx [file pone.0323613.s006.docx]

Comments on “ **Effect of PGPR on growth and nutrient utilization of Elymus nutans Griseb at different temperatures”**

In Abstract: it is mentioned that ‘our results showed that this PGPR can promote the growth of Elymus nutans Griseb on Tibetan plateau and improve its nutrient utilization efficiency’

Explain- what is 'this PGPR'?

Either all the tested five (5) PGPR or specific strain?

**The Reviewer.**
