# Supplementary material for: Effect of PGPR on growth and nutrient utilization of Elymus nutans Griseb at different temperatures
Source: PLoS One. 2025 May 12;20(5):e0323613. doi: 10.1371/journal.pone.0323613 (PMC12068589; doi:10.1371/journal.pone.0323613)
Supplement: Attachment — Submitted filename: Response_to_Reviewers_auresp_3.docx [file pone.0323613.s008.docx]

**Response to Reviewers and Editor**

Manuscript Number: **PONE-D-24-56644**

Effect of PGPR on growth and nutrient utilization of *Elymus nutans* Griseb at different temperatures

Dear Editor and Reviewers,

Thank you very much for your comments and professional advice. These opinions help to improve academic rigor of our article. Based on your suggestion and request we have made corrected modifications on the revised manuscript. We hope that our work can be improved again. We would like to show the details as follows:

**To Reviewer #3**:

1.In Abstract: it is mentioned that ‘our results showed that this PGPR can promote the growth of Elymus nutans Griseb on Tibetan plateau and improve its nutrient utilization efficiency’. Explain- what is 'this PGPR'? Either all the tested five (5) PGPR or specific strain?

Answer: Thank you for the reviewer's comments. We have elaborated on this PGPR in the manuscript, and it refers to all the tested five (5) PGPR.

**To Editor：**

1."In order to show the growth-promoting effects of selected PGPR isolated from the rhizosphere soil of Elymus nutans Griseb on the Tibetan Plateau." cannot be a stand-alone sentence: main clause is missed.

Answer: Thank you for reviewing our manuscript and providing valuable comments. We have carefully revised the sentence structure issue you pointed out. The original sentence, “In order to show the growth-promoting effects of selected PGPR isolated from the rhizosphere soil of *Elymus nutans* Griseb on the Tibetan Plateau,” was indeed missing a main clause and couldn't stand alone. We have modified it to:“to demonstrate the growth-promoting effects of the selected PGPR, we conducted a study.” We place great importance on your feedback and have ensured that all sentences are structurally complete and grammatically correct. Thank you again for your valuable comments and support.

2. "By isolating and purifying the rhizosphere bacteria from the rhizosphere soil of Elymus nutans Griseb (EnG) in the Qinghai-Tibet Plateau, use the selective culture medium to determine whether the strains have plant growth-promoting ability and measure the magnitude of their plant growth-promoting ability". This sound as a protocol where someone is giving you instructions.

Answer: Thank you for reviewing our manuscript and providing valuable comments. We have carefully revised the sentence structure issue you pointed out. The original sentence, “By isolating and purifying the rhizosphere bacteria from the rhizosphere soil of *Elymus nutans* Griseb (*En*G) in the Qinghai-Tibet Plateau, use the selective culture medium to determine whether the strains have plant growth-promoting ability and measure the magnitude of their plant growth-promoting ability.” had an imperative tone and was less natural. We have modified it.

3.  "Five selected strains (S1, S2, S3, S4 and S5) with high growth promoting ability were screened by the determination of growth-promoting ability". Really confusing and repetitive.

Answer: Thank you for reviewing our manuscript and providing valuable comments. We have carefully revised the sentence structure issue you pointed out. The original sentence, “Five selected strains (S1, S2, S3, S4 and S5) with high growth promoting ability were screened by the determination of growth-promoting ability,” was indeed confusing and repetitive. We have modified it to: “Five strains (S1, S2, S3, S4, and S5) with high growth-promoting ability were identified through growth-promoting assays.”

4.  "Inoculate the purified strains onto Nitrogen free medium (NFM)..." This sound as a protocol where someone is instructing you to inoculate the strain on different media. In the article, you must write "Strains were inculated onto NFM..." etc.

Answer: Thank you for reviewing our manuscript and providing valuable comments. We have carefully revised the sentence structure issue you pointed out. The original sentence, “Inoculate the purified strains onto Nitrogen free medium (NFM)...” We have modified it to: “Strains were inculated onto NFM...”

5. Therefore, it is necessary that the manuscript is revised by a native english speaker before it can be accepted and published.

Answer: Thank you for reviewing our manuscript and providing valuable comments. We attach great importance to your suggestion that the manuscript should be revised by a native English speaker before it can be accepted and published. To ensure that the language quality of the manuscript meets the standards of international academic journals, we have invited a native English speaker to conduct a comprehensive language polishing and proofreading.

6. Another important aspect to be clarified concerns the statistical analysis: it is stated that both least significant difference (LSD) and Tukey test (HSD) were used. However, in the graphs where post-hoc letters are reported, it is not indicated which post-hoc method was used. Considering that these two post-hoc tests are very different (LSD the most permissive, HSD the most stringent one), it is really necessary that this is clearly indicated: significant differences assessed by HSD have a much greater value that those assessed by LSD!!

Answer: Thank you for reviewing our manuscript and providing valuable comments. We used the Tukey test (HSD) in the significance test. In this regard, we have made revisions in the Statistical analyses section.
